# Genome-wide meta-analysis reveals common splice site acceptor variant in *CHRNA4* associated with nicotine dependence

**DOI:** 10.1038/tp.2015.149

**Published:** 2015-10-06

**Authors:** D B Hancock, G W Reginsson, N C Gaddis, X Chen, N L Saccone, S M Lutz, B Qaiser, R Sherva, S Steinberg, F Zink, S N Stacey, C Glasheen, J Chen, F Gu, B N Frederiksen, A Loukola, D F Gudbjartsson, I Brüske, M T Landi, H Bickeböller, P Madden, L Farrer, J Kaprio, H R Kranzler, J Gelernter, T B Baker, P Kraft, C I Amos, N E Caporaso, J E Hokanson, L J Bierut, T E Thorgeirsson, E O Johnson, K Stefansson

**Affiliations:** 1 Behavioral and Urban Health Program, Behavioral Health and Criminal Justice Research Division, Research Triangle Institute International, Research Triangle Park, NC, USA; 2 deCODE Genetics/Amgen, Reykjavik, Iceland; 3 Research Computing Division, Research Triangle Institute International, Research Triangle Park, NC, USA; 4 Virginia Institute for Psychiatric and Behavioral Genetics, Department of Psychiatry, Virginia Commonwealth University, Richmond, VA, USA; 5 Nevada Institute of Personalized Medicine and Department of Psychology, University of Nevada, Las Vegas, NV, USA; 6 Department of Genetics, Washington University in St. Louis, St. Louis, MO, USA; 7 Department of Biostatistics and Informatics, University of Colorado Anschutz Medical Campus, Aurora, CO, USA; 8 Department of Public Health, Faculty of Medicine, University of Helsinki, Helsinki, Finland; 9 Department of Medicine (Biomedical Genetics), Boston University School of Medicine, Boston, MA, USA; 10 Genetic Epidemiology Branch, Division of Cancer Epidemiology and Genetics, National Cancer Institute, National Institutes of Health, United States Department of Health and Human Services, Bethesda, MD, USA; 11 Iowa Department of Public Health, Des Moines, IA, USA; 12 Institute of Epidemiology I, German Research Center for Environmental Health, Neuherberg, Germany; 13 Department of Genetic Epidemiology, University of Göttingen—Georg-August University Göttingen, Göttingen, Germany; 14 Department of Psychiatry, Washington University School of Medicine, St. Louis, MO, USA; 15 Department of Neurology, Boston University School of Medicine, Boston, MA, USA; 16 Department of Ophthalmology, Boston University School of Medicine, Boston, MA, USA; 17 Department of Genetics and Genomics, Boston University School of Medicine, Boston, MA, USA; 18 Department of Epidemiology, Boston University School of Public Health, Boston, MA, USA; 19 Department of Biostatistics, Boston University School of Public Health, Boston, MA, USA; 20 National Institute for Health and Welfare, Helsinki, Finland; 21 Institute for Molecular Medicine, University of Helsinki, Helsinki, Finland; 22 Department of Psychiatry, University of Pennsylvania Perelman School of Medicine, Philadelphia, PA, USA; 23 VISN 4 Mental Illness Research, Education and Clinical Center, Philadelphia VA Medical Center, Philadelphia, PA, USA; 24 Department of Psychiatry, Yale University School of Medicine, New Haven, CT, USA; 25 Department of Genetics, Yale University School of Medicine, New Haven, CT, USA; 26 Department of Neurobiology, Yale University School of Medicine, New Haven, CT, USA; 27 VA CT Healthcare Center, Department of Psychiatry, West Haven, CT, USA; 28 Center for Tobacco Research and Intervention, University of Wisconsin, Madison, WI, USA; 29 Department of Epidemiology, Harvard University School of Public Health, Boston, MA, USA; 30 Department of Biostatistics, Harvard University School of Public Health, Boston, MA, USA; 31 Department of Community and Family Medicine, Geisel School of Medicine at Dartmouth, Hanover, NH, USA; 32 Department of Genetics, Geisel School of Medicine at Dartmouth, Hanover, NH, USA; 33 Department of Biomedical Data Science, Geisel School of Medicine at Dartmouth, Hanoven, NH, USA; 34 Department of Epidemiology, University of Colorado Anschutz Medical Campus, Aurora, CO, USA; 35 Fellow Program and Behavioral Health and Criminal Justice Research Division, Research Triangle Institute International, Research Triangle Park, NC, USA; 36 Faculty of Medicine, University of Iceland, Reykjavik, Iceland

## Abstract

We conducted a 1000 Genomes–imputed genome-wide association study (GWAS) meta-analysis for nicotine dependence, defined by the Fagerström Test for Nicotine Dependence in 17 074 ever smokers from five European-ancestry samples. We followed up novel variants in 7469 ever smokers from five independent European-ancestry samples. We identified genome-wide significant association in the alpha-4 nicotinic receptor subunit (*CHRNA4)* gene on chromosome 20q13: lowest *P*=8.0 × 10^−9^ across all the samples for rs2273500-C (frequency=0.15; odds ratio=1.12 and 95% confidence interval=1.08–1.17 for severe vs mild dependence). rs2273500-C, a splice site acceptor variant resulting in an alternate *CHRNA4* transcript predicted to be targeted for nonsense-mediated decay, was associated with decreased *CHRNA4* expression in physiologically normal human brains (lowest *P*=7.3 × 10^−4^). Importantly, rs2273500-C was associated with increased lung cancer risk (*N*=28 998, odds ratio=1.06 and 95% confidence interval=1.00–1.12), likely through its effect on smoking, as rs2273500-C was no longer associated with lung cancer after adjustment for smoking. Using criteria for smoking behavior that encompass more than the single ‘cigarettes per day' item, we identified a common *CHRNA4* variant with important regulatory properties that contributes to nicotine dependence and smoking-related consequences.

## Introduction

Cigarette smoking is a major contributor to cancer, vascular disease and lung disease, and the leading cause of preventable mortality worldwide.^1^ Nicotine dependence is heritable,^2^ and genome-wide association study (GWAS) analyses of smoking behaviors and nicotine dependence have unequivocally identified single nucleotide polymorphism (SNP) associations within nicotinic acetylcholine receptor gene clusters on chromosomes 15q25 (*CHRNA5-CHRNA3-CHRNB4*) and 8p11 (*CHRNB3-CHRNA6*).^3, 4, 5, 6, 7, 8, 9^ The largest prior GWAS of smoking behavior was conducted using very large sample sizes (*N* up to 74 053) and phenotypes such as smoking history (ever vs never), age of onset, smoking cessation (former vs current) and cigarettes per day (CPD).^5, 6, 7^

To identify additional genetic loci, we conducted the largest GWAS meta-analysis for nicotine dependence to date. We used the Fagerström Test for Nicotine Dependence (FTND), a six-item questionnaire with scores that range from 0 to 10 and indicate the level of physiological dependence on nicotine.^10, 11^ The FTND focuses on the core criteria for dependence, including heavy use/tolerance (for example, CPD) and withdrawal (for example, time to first cigarette in the morning), and although it does not capture some factors such as craving, the FTND remains the strongest predictor of smoking cessation among the primary measures of nicotine dependence.^12^ It has also been shown to provide a robust measure of nicotine dependence across different settings and populations.^13^

In our study, we categorized nicotine dependence as mild (FTND score 0–3 or low-level smoking), moderate (FTND score 4–6) or severe (FTND score 7–10) among study participants, all of European-ancestry, who reported smoking more than 100 cigarettes in their lifetime. We conducted a 1000 Genomes–imputed GWAS meta-analysis of nicotine dependence across five study samples (total *N*=17 074), identified the alpha-4 nicotinic receptor subunit (*CHRNA4*) gene as a novel genome-wide significant locus, tested top *CHRNA4* variants for replication in five independent study samples (total *N*=7469) and conducted follow-up association testing with lung cancer using six study samples (total *N*=12 160 cases and 16 838 controls). Our results revealed that rs2273500, a splice site acceptor SNP with important regulatory effects for *CHRNA4*, was associated with risk of developing both nicotine dependence and lung cancer.

## Materials and Methods

All protocols used in this study received institutional review board approval at their respective sites, and all the study participants or their legal representatives provided informed consent.

### Discovery study samples, the FTND and quality control

Five samples were used to conduct our GWAS meta-analysis of nicotine dependence: deCODE Genetics,^4^ Environment and Genetics in Lung Cancer Etiology Study (EAGLE),^14, 15^ Chronic Obstructive Pulmonary Disease Gene (COPDGene) Study,^16^ Collaborative Genetic Study of Nicotine Dependence (COGEND)^3^ and Study of Addiction: Genetics and Environment (SAGE*).^9^ These study participants, all of European-ancestry, had genome-wide SNP genotypes and FTND^10, 11^ scores to define nicotine dependence among participants who reported smoking more than 100 cigarettes in their lifetime. Quality control (QC) was conducted on genotyped participants and SNPs in each sample using PLINK^17^ unless otherwise stated.

We used the FTND range of scores to categorize participants' nicotine dependence as mild (FTND score 0–3), moderate (FTND score 4–6) or severe (FTND score 7–10). In the deCODE Genetics sample only, 4313 low-intensity smokers (10 or fewer CPD) with no FTND data available were added into the mild category. Among participants with both CPD and FTND data available in two samples (deCODE and COGEND), we found high concordance rates (~90%) between a report of 10 or fewer CPD and FTND scores of 3 or less, meaning that the inclusion of low-intensity smokers would enable us to increase sample size with little phenotype misclassification. As presented in Table 1, our final analysis data sets totaled 17 074 participants of European-ancestry: 9137 with mild, 4881 with moderate and 3056 with severe nicotine dependence.

deCODE Genetics represents a large population-based Icelandic sample. The Icelandic cigarette smoking data have been described elsewhere.^4^ All Icelandic subjects in the study of smoking-related phenotypes, including Icelandic population controls, were originally recruited for different genetic studies, conducted over 18 years (1996–2014) at deCODE Genetics. Questionnaire data were used to gather information on CPD and the FTND score. The deCODE Genetics studies were approved by the Data Protection Commission of Iceland and the National Bioethics Committee of Iceland. Personal identifiers associated with phenotypic information and blood samples were encrypted using a third-party encryption system.^18^ Altogether, we included data for 9090 smokers who were genotyped using SNP arrays in one of several GWAS conducted by deCODE Genetics. Genotyping was carried out using Illumina (San Diego, CA, USA) chips. QC was carried out as previously described.^19^

EAGLE is a population-based study of newly diagnosed lung cancer cases and matched controls from the Italian region of Lombardy, as described elsewhere.^15^ EAGLE participants, aged 35 to 79 years old, were genotyped on the Illumina HumanHap550v3 BeadChip array, as part of the GENEVA (Gene Environment Association Studies Initiative).^20^ We obtained their genome-wide SNP genotypes, overall FTND score and other phenotype data via the database of Genotypes and Phenotypes (dbGaP; accession number phs000093.v2.p2). Additional analyses were conducted using the specific FTND item scores by the original study investigators. We began by applying all participant-level and SNP-level QC procedures that were recommended as part of the dbGaP release and then applied our own standard set of QC procedures (Supplementary Information). There were 3006 EAGLE participants included in our study.

COPDGene is a multicenter observational sample primarily focused on identifying genetic risk factors for COPD, as previously described.^16^ Recruited participants were non-Hispanic white or African American and aged 45 to 80 years old, who reported a history of smoking (currently or past) and 10 or more cigarette pack-years. FTND was assessed in current smokers only. We used the non-Hispanic white current smoking participants for this study. Among the COPD cases, disease severity was staged according to the Global Initiative for Chronic Obstructive Lung Disease criteria, which are based on post-bronchodilator pulmonary function measures. COPD controls had pulmonary function measures in the normal range for their age and height, separately by sex. Exclusion criteria for acute and chronic respiratory disease, cancer and other conditions were used. COPDGene participants were genotyped on the Illumina HumanOmni1-Quad BeadChip array. After applying our standard QC procedures (Supplementary Information), there were 2211 COPDGene participants for analysis.

COGEND, a community-based case–control study of nicotine-dependent smokers vs smokers who never developed nicotine-dependence symptoms, began recruiting participants in 2001 from St. Louis and Detroit through telephone screening to identify current smokers aged 25 to 44 years old.^3^ The FTND was administered to determine study eligibility. Current smokers with an FTND score of ⩾4 were recruited as nicotine-dependent cases, and smokers who reported >100 cigarettes during their lifetime but an FTND score of 0 or 1 were recruited as controls. COGEND participants were genotyped on either the Illumina Human1M-Duo BeadChip array, as part of SAGE,^9^ or the Illumina HumanOmni2.5 BeadChip array as part of GENEVA.^20^ In each subset, genotyped SNPs with a call rate >98% and HWE *P*⩾1 × 10^−4^ were retained. We combined the subsets and removed duplicated participants and first-degree relatives. To circumvent bias that may arise from conducting imputation on subjects genotyped on different arrays, we carried forward only the SNPs genotyped at the intersection of the different arrays.^21^ After applying our standard QC procedures (Supplementary Information) on the combined COGEND sample, there remained 1935 participants for our study.

The final GWAS sample consisted of the remaining SAGE study participants. The full SAGE sample included participants from COGEND, the Collaborative Study on the Genetics of Alcoholism^22^ and the Family Study of Cocaine Dependence.^23^ For our study, we excluded the COGEND participants to avoid redundancy. Because the remaining Collaborative Study on the Genetics of Alcoholism and Family Study of Cocaine Dependence participants were ascertained as part of case–control studies of addictive disorders and all were ascertained from sites in the United States, we analyzed them together as done in previous GWAS analyses.^9^ We henceforth refer to this sample as SAGE*. We obtained their Illumina Human1M-Duo BeadChip genotypes and phenotype data via dbGaP accession number phs000092.v1.p1. After applying our standard QC procedures (Supplementary Information), there remained 832 participants for analysis.

For the four samples of non-isolated populations (EAGLE, COPDGene, COGEND and SAGE*), we used the STRUCTURE program^24^ to compute the ancestral proportions of all study participants using, based on comparison to the HapMap reference populations of Chinese (denoted CHB), European Americans (denoted CEU) and African Americans (denoted ASW) using 10 000 SNPs randomly distributed across the genome. We excluded outlying participants with ⩾25% Asian and/or African American proportions.

### 1000 Genomes imputation

Genotype imputation was conducted in each non-isolated sample using IMPUTE2 (ref. 25) with reference to the 1000 Genomes ALL phase I integrated variant set.^25,26^ Additional details are provided in the Supplementary Information. Following imputation and removal of SNPs and insertions/deletions (indels) with minor allele frequency <0.01 in the 1000 Genomes EUR panel (collection of five European-ancestry populations), we tested 8 548 225 SNPs and 1 395 199 indels for association with nicotine dependence across the samples. We used the info metric to evaluate the SNP/indel imputation quality rather than imposing an imputation quality filter and possibly missing truly associated SNPs/indels.^27^ The SNP and indel genotype probabilities were converted to dosages and used in the regression model for association testing with nicotine dependence to account for any imputation uncertainty.^28^

For deCODE, genotype imputation was conducted by long-range phasing of all chip-genotyped individuals with methods described previously.^29^ Sequence variants were imputed from the deCODE whole-genome sequencing effort into 104 220 chip-genotyped Icelanders, who had been phased with long-range phasing, using the same model as used by IMPUTE.^28^

### Statistical analyses: testing genome-wide SNP and indel associations with nicotine dependence

The genotyped and 1000 Genomes–imputed SNPs and indels were tested for association with categorical nicotine dependence (mild, moderate and severe) using ProbAbel software^30^ in each sample with linear regression models that included age, sex and sample-specific covariates (if applicable, see Supplementary Information). The four European American and Italian samples also included principal component eigenvectors to minimize bias owing to population stratification; for each sample, we selected the number of eigenvectors needed to account for >75% of the variability in nicotine dependence.

The sample-specific GWAS results were combined in METAL^31^ using inverse variance-weighted meta-analysis. The standard GWAS threshold (*P*<5 × 10^−8^) was used to declare statistically significant results. The *I*^2^ index was used to assess heterogeneity across samples.^32^

### Follow-up SNP/indel association testing with nicotine dependence in independent samples

For any novel region having genome-wide significant association with nicotine dependence, we selected SNPs and indels associated at meta-analysis *P*<5 × 10^−5^ for follow-up testing across five independent European-ancestry samples: Yale-Penn study,^33, 34, 35^ Finnish Twin Cohort Study (FTC),^36, 37^ University of Wisconsin-Transdisciplinary Tobacco Use Research Center (UW-TTURC),^12^ Genetic Association Information Network (GAIN) GWAS of Schizophrenia and Molecular Genetics of Schizophrenia—nonGAIN Sample.

Participants in the Yale-Penn study were from small nuclear families and unrelated individuals recruited in the eastern United States in the course of studies of the genetics of alcohol, cocaine or opioid dependence. Nicotine dependence had no role in subject selection. Yale-Penn participants were administered the Semi-Structured Assessment for Drug Dependence and Alcoholism and were genotyped on the Illumina HumanOmni1-Quad v1.0 microarray. Participants with missing rate >2% were excluded. SNPs with missing rate >2%, HWE *P*<1 × 10^−4^ and with significantly different minor allele frequency across genotyping labs (Yale or CIDR) were set to missing before imputation. SNP genotype imputation was performed with IMPUTE2 (ref. 25) using genotyped SNPs and the March 2012 1000 Genomes ALL reference panel. Genetic relationships were examined by calculating pairwise identity-by-state estimates using PLINK.^17^ Sample duplicates (identity-by-state >90%) were removed, pairs of individuals whose identity-by-state proportions did not match their reported genetic relationship were assigned to two different families and pairs of individuals who shared >25% of their alleles identity-by-state were assigned to the same family. Participants with gender discordance (*F*_ST_<0.2 for chromosome X SNPs to confirm females and F_ST_ >0.8 to confirm males) were also removed, unless their true identity could be determined. To verify and correct potential misclassification of self-reported race, we compared the GWAS data from all participants with HapMap phase III reference genotypes. Association tests were performed on *N*=2116 using linear regression models adjusted for age, sex and the first three principal component eigenvectors computed using Eigensoft and embedded in generalized estimating equations to correct for correlations among relatives.

Altogether, 2374 participants from FTC were included for replication testing. These participants originated from the following cohorts: the Nicotine Addiction Genetics study of adult twins born in 1938–1957 and concordant for ever smoking, and their family members (mainly siblings); a population-based longitudinal study of five consecutive birth cohorts (1983–1987) of Finnish twins (FinnTwin12 sample); and a population-based longitudinal study of five consecutive birth cohorts (1975–1979) of Finnish twins (FinnTwin16 sample).^36, 37, 38^ Genotyping was done with the Illumina Human670-QuadCustom BeadChip (at the Wellcome Trust Sanger Institute) and the Illumina HumanCoreExome BeadChip (at the Wellcome Trust Sanger Institute and at the Broad Institute of MIT and Harvard). FTC samples were imputed with a large number of population samples, separately by genotyping array, with reference to 1000 Genomes (Phase I integrated variant set release [SHAPEIT2] in National Center for Biotechnology Information (NCBI) build 37 [hg19] coordinates) at the Institute for Molecular Medicine Finland. After imputation, FTC samples were merged together. Standardized residuals of the categorical FTND phenotype, regressed against SNP dosages, age, sex, birth cohort and the 10 first principal components (calculated from genome-wide genotype data), were used in QFAM association test in PLINK.^17^ The resulting regression coefficients are mathematically equivalent to regression coefficients when using raw phenotypes that are linearly regressed on SNP genotypes and covariates, enabling us to combine the FTC results with the others in meta-analysis as done elsewhere.^39^

UW-TTURC participants were recruited for nicotine dependence and smoking cessation treatment clinical trials in Madison and Milwaukee, Wisconsin beginning in 2001.^12^ We obtained their Illumina HumanOmni2.5 genotypes, FTND scores and other phenotypic data via dbGaP accession number phs000404.v1.p1. After applying our standard QC procedures (Supplementary Information), there were 1534 UW-TTURC participants included in our study.

The GAIN and nonGAIN samples originated from the same Molecular Genetics of Schizophrenia study. These companion samples were genotyped separately, using the same platform (Affymetrix 6.0). Data from half of the Molecular Genetics of Schizophrenia study participants genotyped under the auspices of GAIN were obtained via dbGaP accession number phs000021.v3.p2, and data from the other half of the Molecular Genetics of Schizophrenia study participants (nonGAIN) were obtained via dbGaP accession number phs000167.v1.p1. We used only schizophrenia controls from GAIN (*N*=774) and nonGAIN (*N*=671), for which we applied our standard set of QC procedures (Supplementary Information).

Across the replication samples, the 1000 Genomes–imputed additive genotype dosages for selected SNPs and indels were tested for association with FTND-defined nicotine dependence (mild, moderate and severe) using linear regression models unless otherwise stated. Adjustments were made for age, sex and eigenvectors (first three for Yale-Penn and the number needed to account for >75% of the phenotypic variability for UW-TTURC, GAIN and nonGAIN). Sample-specific results were combined, first across the replication samples and then across all GWAS and replication samples, using inverse variance-weighted meta-analysis. Odds ratio estimates, computed as e^β^ for moderate vs mild dependence and e^2β^ for severe vs mild dependence, were compared across all the samples using the Forest Plot Viewer.^40^

### Bioinformatics analyses

Linkage disequilibrium structure was discerned using LocusZoom^41^ or Haploview^42^ with reference to the 1000 Genomes populations of European-ancestry (denoted EUR). SNP annotations were taken from the Ensembl genome browser.^43^

### Splicing QTL analyses

To evaluate the regulatory potential of novel intronic SNPs in *CHRNA4* associated with nicotine dependence, we first used RNA-seq and genotype data from the pilot phase of the Genotype-Tissue Expression (GTEx) project to investigate SNP effects on splicing. GTEx captures a wide range of tissues collected post-mortem from donors of any age from 21 to 70 years old, sex and racial/ethnic group, although the data set is comprised mostly of European-ancestry participants.^44, 45^ Exclusions were made for HIV infection or high-risk behaviors, viral hepatitis, metastatic cancer, recent chemotherapy or radiation therapy, recent whole-blood transfusion or body mass index at the extremes. We focused on the liver tissue, which had the highest *CHRNA4* expression levels among the GTEx tissues, and all brain tissues combined, which had lower *CHRNA4* expression levels but high relevance for nicotine dependence.

An alternate *CHRNA4* transcript (uc010 gke.1) encodes an additional exon (designated 4.1 in Supplementary Figure 1) located between exons 4 and 5 of the major transcript (uc002.yes.2). We used split read counts (reads crossing an exon:exon boundary) and a chi-square test to evaluate the relative efficiency of splicing events, which utilize the exon 4 splice donor and to query whether the efficiency varied by SNP genotype.

### Expression QTL analyses

To further evaluate the regulatory potential of novel nicotine dependence-associated SNPs, we conducted *in silico* testing of SNP associations with transcript-level *CHRNA4* mRNA expression using the Brain expression quantitative trait loci (eQTL) Almanac.^46^ This resource contains genome-wide *cis*-eQTL results, which were generated using Illumina Omni1-Quad and Immunochip SNP genotypes, followed by 1000 Genomes imputation and mRNA expression levels from Affymetrix Human Exon 1.0 ST arrays on 134 European-ancestry participants in the UK Brain Expression Consortium data set.^46^ The participants, mostly adults, were free of neurodegenerative disorders and were collected irrespective of nicotine dependence or any other substance phenotype. Transcript-level and exon-level expression measurements were made across 10 regions of the post-mortem brain samples: cerebellar cortex, frontal cortex, hippocampus, inferior olivary nucleus (sub-dissected from the medulla), occipital cortex, putamen (at the level of the anterior commissure), substantia nigra, temporal cortex, thalamus (at the level of the lateral geniculate nucleus) and intralobular white matter. In addition to the single *CHRNA4* transcript probe, probes were available in each of the exons for the transcripts that provide templates for a full-length CHRNA4 protein; no probe was available for the ancillary exon 4.1 of the alternative transcript that results in a truncated protein.

### Follow-up SNP/indel association testing with lung cancer

For top nicotine dependence-associated SNPs, we performed *in silico* testing of their associations with lung cancer using 1000 Genomes–imputed GWAS meta-analysis results generated elsewhere.^47^ *CHRNA4* SNP results were provided using 12 160 cases and 16 838 controls from six samples of European-ancestry: the International Agency for Research on Cancer,^48^ Institute of Cancer Research,^49^ MD Anderson Cancer Center,^50^ National Cancer Institute (comprising EAGLE and the Prostate, Lung, Colon and Ovary Study Cancer Screening Trial),^51^ Samuel Lunenfeld Research Institute in Toronto^51^ and Helmholtz-Gemeinschaft Deutscher Forschungszentren in Germany.^51^ SNP associations, adjusted for age, sex and the first two principal component eigenvectors, were evaluated using all lung cancer cases and controls and using subsets of 3718 adenocarcinoma and 3422 squamous cell carcinoma cases. SNP associations with lung cancer were also tested with adjustment for smoking history (ever vs never) and pack-years of smoking among ever smokers (0 for never smokers) in three of the case–control samples, which together comprised 16% never smokers and 84% ever smokers.

## Results

Our GWAS meta-analysis included 17 074 ever smokers from five European-ancestry samples (Table 1). We used the FTND to categorize participants' dependence as mild (*N*=9137 with FTND score 0–3 or low-level smoking), moderate (*N*=4881 with FTND score 4–6) or severe (*N*=3056 with FTND score 7–10). We tested genotyped and 1000 Genomes–imputed SNPs and indels for association with categorical nicotine dependence in each sample and then combined the GWAS results using inverse variance-weighted meta-analysis with genomic control correction^52^ applied to each sample. Meta-analysis results for the 9.9 million tested SNPs/indels had no indication of bias (*λ*_gc_=0.97, Supplementary Figure 2).

**Figure 1:**
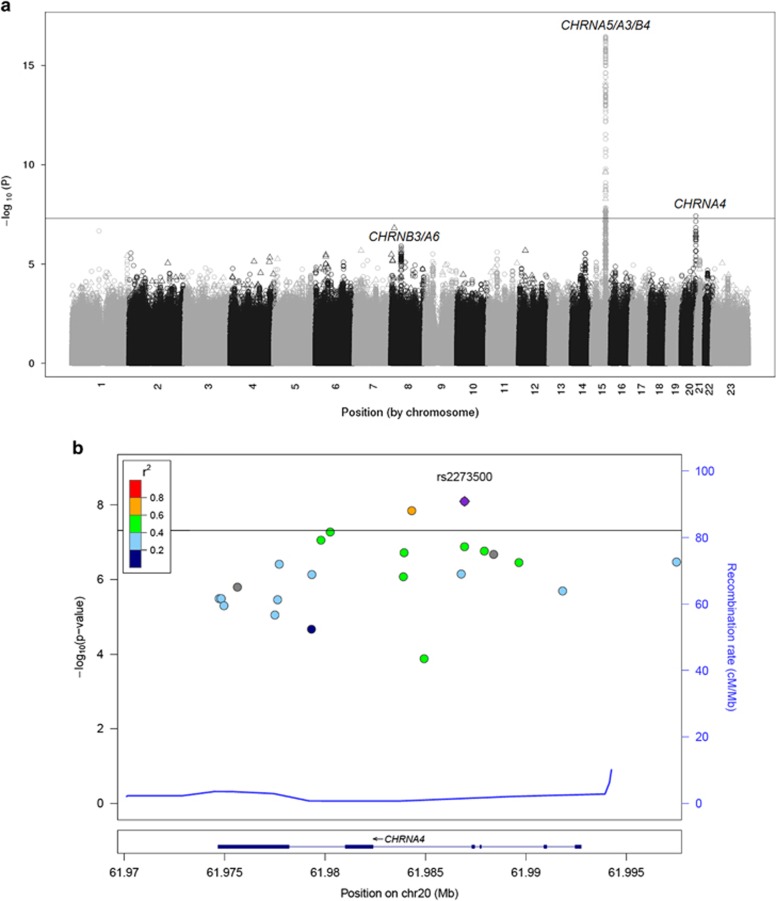
SNP and indel associations with nicotine dependence from meta-analyses of discovery and independent replication samples, all of European-ancestry. The −log_10_ (meta-analysis *P*) results are plotted by chromosomal position: (**a**) genome-wide meta-analysis results across the five discovery samples (total *N*=17 074) for 9.9 million genotyped and imputed SNPs and indels tested for association with nicotine dependence (SNPs shown as circles and indels shown as triangles) with minor allele frequency >0.01 and (**b**) regional meta-analysis results across all 10 discovery and replication samples (total *N*=24 543) in the *CHRNA4* gene on chromosome 20q13. For the regional plot, the *r*^2^ values between the top SNP (rs2273500, shown in purple) and other SNPs in the flanking region were based on the 1000 Genomes European reference panel (denoted EUR); the indels are shown in gray. The solid black lines mark the genome-wide statistical significance threshold (meta-analysis *P*<5 × 10^−^^8^). indel, insertion/deletion; SNP, single nucleotide polymorphism.

A novel genome-wide significant association was observed on chromosome 20q13 (lowest *P*=3.8 × 10^−8^ for rs4809294, Figure 1a). In total, 23 SNPs/indels on chromosome 20q13 were associated at *P*<5 × 10^−5^ (Supplementary Table 1). We also observed associations within the known gene clusters on chromosomes 15q25 (*CHRNA5-CHRNA3-CHRNB4*, lowest *P*=3.5 × 10^−17^) and 8p11 (*CHRNB3-CHRNA6*, lowest *P*=1.2 × 10^−6^) (Figure 1a and Supplementary Tables 2 and 3). None of the 23 top-associated *CHRNA4* SNPs interacted with previously established SNPs in the known regions (Supplementary Table 4).

**Figure 2:**
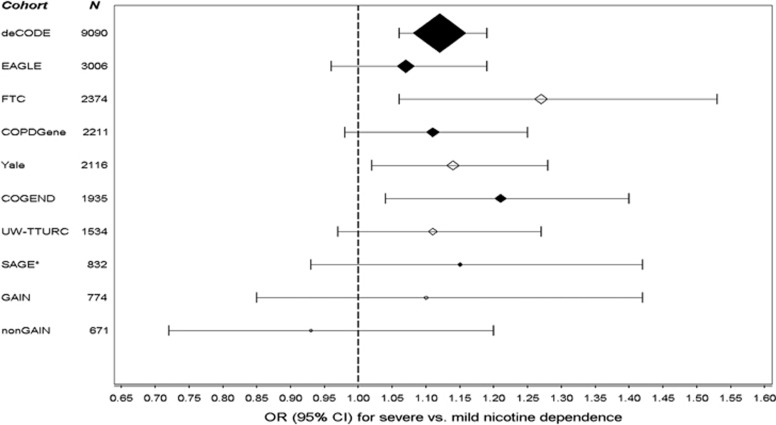
Association of the rs2273500 minor allele (C) with severe vs mild nicotine dependence across all the samples. rs2273500 had the lowest *P*-value for its association with nicotine dependence in meta-analysis across all the samples, which are sorted by their size. Black-filled diamonds indicate the discovery samples, and open diamonds indicate the replication samples. The odds ratio (OR) estimates are shown proportional to the sample size. CI, confidence interval; COGEND, Collaborative Genetic Study of Nicotine Dependence; COPDGene, Chronic Obstructive Pulmonary Disease Gene Study; EAGLE, Environment and Genetics in Lung Cancer Etiology Study; FTC, Finnish Twin Cohort Study; GAIN, Genetic Association Information Network GWAS of schizophrenia; nonGAIN, Molecular Genetics of Schizophrenia—nonGAIN sample; SAGE*, Study of Addiction: Genetics and Environment (*indicates that overlapping COGEND participants were excluded); UW-TTURC, University of Wisconsin-Transdisciplinary Tobacco Use Research Center.

We tested the 23 *CHRNA4* SNPs/indels associated with nicotine dependence at *P*<5 × 10^−5^ for independent replication using 7469 ever smokers from five European-ancestry samples (Table 1 and Supplementary Table 5). Two SNPs had genome-wide significant associations with nicotine dependence across all the samples (Table 2): rs2273500 and rs6011779, which are in strong linkage disequilibrium in European-ancestry individuals (D′=1.00 and *r*^2^=0.70, Figure 1b and Supplementary Figure 3). Their minor alleles (frequency=0.15 and 0.20) were associated with greater nicotine dependence risk, as demonstrated in Figure 2 for the top SNP rs2273500: meta-analysis odds ratio=1.06 (95% confidence interval 1.04–1.08) for moderate vs mild nicotine dependence and odds ratio=1.12 (95% confidence interval 1.08–1.17) for severe vs mild nicotine dependence. None of the tested SNPs/indels showed significant evidence for between-sample heterogeneity (Supplementary Table 6).

The top SNP from the overall meta-analysis, rs2273500, was imputed well across our discovery and replication samples, with info values ranging from 0.8 to 1.0 (Supplementary Tables 1 and 5). See the Supplementary Information for an evaluation of the agreement between rs2273500 imputed dosages and directly observed genotypes. rs2273500 is in some linkage disequilibrium with rs4809294, the top GWAS-identified SNP (D′=0.97 and *r*^2^=0.37, Supplementary Figure 3). rs4809294 (replication meta-analysis *P*=0.36) may tag an underlying causal variant with varying linkage disequilibrium across the different populations, and its effect size may have been overestimated in the discovery GWAS meta-analysis.^53^

Prior genome-wide studies of CPD did not identify the *CHRNA4* region at genome-wide significance, likely owing to differences in phenotype definition and lower SNP coverages.^3, 4, 5, 6, 7, 8, 9, 38, 54, 55^ Regarding phenotype definition, we found that our top SNP rs2273500 was most significantly associated with time to first cigarette in the morning (*P*=2.3 × 10^−8^, Supplementary Table 7). In contrast, for the missense *CHRNA5* SNP rs16969968 that has reproducible associations with both nicotine dependence (Supplementary Table 1) and CPD,^5, 6, 7^ the lowest *P*-value, by far, was observed for CPD (*P*=9.4 × 10^−24^, Supplementary Table 8). rs16969968 has previously also shown strong association with cotinine levels, which reflect recent nicotine intake.^56, 57^

Regarding SNP coverage, our top nicotine dependence-associated *CHRNA4* variants are 1000 Genomes–imputed SNPs not present on genotyping arrays or imputable from HapMap phase II in prior GWAS. Our most significantly associated SNP available in HapMap phase II (rs4809539, *r*^2^=0.19 and D′=0.67 with rs2273500 in EUR) had meta-analysis *P*=3.5 × 10^−4^. We evaluated the *CHRNA4* region further using meta-analysis results from the largest prior GWAS of CPD by the Tobacco and Genetics Consortium.^7^ These results are presented in Supplementary Table 9. Among 57 HapMap phase II SNPs in the region (*r*^2^ ranging from 0 to 0.61 with rs2273500 in EUR), we found that rs4809539 was also the SNP that was most significantly associated with CPD (meta-analysis *P*=4.8 × 10^−3^); its T allele (frequency=0.06) was associated with higher CPD, consistent with an increased risk of nicotine dependence in our data. These results suggest that use of the FTND phenotype together with 1000 Genomes imputation enabled us to detect *CHRNA4* variant associations with nicotine dependence that were genome-wide significant.

Our top SNP rs2273500 is a splice acceptor variant that changes the sequence at the 3′ end of the intron between exon 4 and an ancillary exon, designated as exon 4.1 in Supplementary Figure 1; the resulting alternate transcript is truncated and predicted to be targeted for nonsense-mediated decay.^43^ To investigate the rs2273500 effect on splicing, we obtained RNA-seq and genotype data from the GTEx project.^44^ Liver (*N*=32) showed the highest *CHRNA4* expression among the GTEx tissues, and transcripts containing exon 4.1 were detected. Using split read counts (reads crossing an exon:exon boundary) to test the efficiency of splicing events that utilize the exon 4 splice donor, we found that rs2273500-C resulted in significant reduction of splicing to exon 4.1 in favor of increased splicing to exon 4.2 and to a cryptic splice acceptor in exon 4.1 (*P*=5.4 × 10^−58^, Supplementary Table 10). These results provide strong evidence that rs2773500 functions as a splicing QTL.

**Figure 3:**
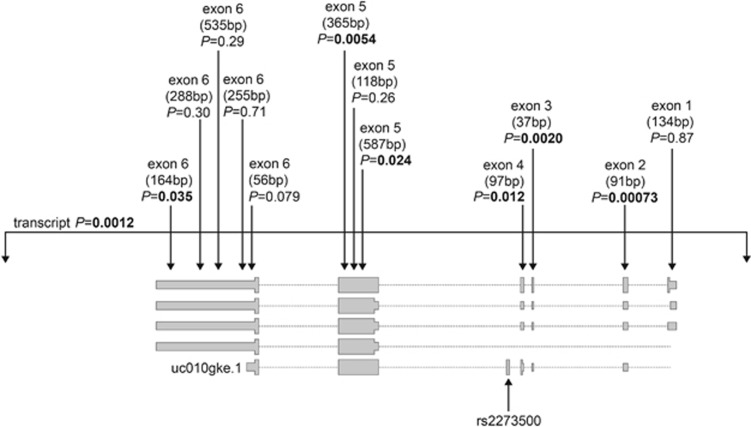
Associations of the top nicotine dependence-associated SNP rs2273500 with transcript-level and exon-level *CHRNA4* mRNA expression in intralobular white matter from 134 European-ancestry participants in the UK Brain Expression Consortium. The probe size (in base pairs) is indicated, and *P*-values <0.05 are shown in bold. SNP, single nucleotide polymorphism.

Transcripts containing exon 4.1 were also observed in GTEx brain tissue samples, but relative to the liver samples, their frequencies were low (Supplementary Table 10). We observed the same pattern, whereby rs2273500-C carriers had reduced splicing to exon 4.1 and increased splicing to exon 4.2, but these differences were not statistically significant (*P*=0.30) likely owing to the limited statistical power with the lower split read counts. However, we were able to evaluate rs2273500 as an eQTL SNP reliably using *CHRNA4* mRNA expression levels measured across 10 regions of physiologically normal human brains from 134 European-ancestry participants in the Brain eQTL Almanac.^46, 58^ At the transcript level (Supplementary Figure 4), we observed that rs2273500-C was associated with decreased *CHRNA4* expression in intralobular white matter. In further evaluation of this brain region (Figure 3), we found that rs2273500-C was associated with decreased expression at *CHRNA4* exons 2–6 (lowest *P*=0.00073).

rs2273500 was next evaluated for its association with lung cancer in 28 998 participants from six European-ancestry case–control samples (Supplementary Table 11).^47^ rs2273500-C was associated with increased lung cancer risk: meta-analysis odds ratio=1.06 (95% confidence interval 1.00–1.12), particularly for squamous cell carcinoma of the lung. However, the association between rs2273500-C and lung cancer was likely mediated by cigarette smoking, as it was no longer associated with lung cancer when adjusted for smoking (Supplementary Table 12).

## Discussion

*CHRNA4* has strong biological plausibility for influencing nicotine dependence and consequently its adverse health effects. Nicotinic acetylcholine receptor genes, including *CHRNA4*, encode subunits that assemble together to form ligand-gated ion channels that respond to the neurotransmitter acetylcholine. Nicotine exposure from cigarette smoking also activates the receptors, triggering dopamine release and influencing the reinforcing effect of nicotine.^59, 60, 61^ The subunits encoded by *CHRNA4* and *CHRNB2* comprise α4β2 receptors, the most abundantly expressed nicotine acetylcholine receptors in the brain. They have a high affinity for nicotine and serve critical roles in nicotine self-administration and its positive reinforcement.^62, 63^ Knock-out mouse models^64, 65^ and knock-in mice with a hypersensitive receptor^66, 67^ have demonstrated that *CHRNA4* is a necessary and sufficient factor for many characteristic behaviors of nicotine dependence, including nicotine-induced reward, tolerance and anxiety relief. A knockdown rat model suggested that α4-containing receptors have a role in nicotine-mediated analgesia, showing that reduced *CHRNA4* expression in brain significantly attenuated sensitivity to nicotine agonist.^68^ This is consistent with our finding that rs2273500-C decreases *CHRNA4* expression and, by lowering sensitivity to nicotine's effects, confers risk for nicotine dependence. Further supporting the relevance of *CHRNA4,* highly effective treatments for smoking cessation, varenicline^69, 70^ and cytisine,^71^ are partial agonists of α4β2 receptors. Interestingly, the item with the strongest rs2273500 association (latency to smoke in the morning) is the most robust of the FTND items in predicting smoking cessation^12^ and has been associated with both lung cancer^72^ and COPD.^73^

Prior studies in humans have supported *CHRNA4* as a susceptibility gene for nicotine dependence and other smoking behaviors. Genome-wide significant linkage signals have been observed in the chromosome 20q13 region containing *CHRNA4* for maximum number of cigarettes smoked in a 24-h period^74^ and in the nearby chromosome 20q11 region for DSM-IV-defined nicotine dependence.^75^ Several candidate gene association studies have focused on *CHRNA4*.^76, 77, 78, 79, 80, 81, 82, 83, 84, 85, 86, 87, 88, 89^ Among the common SNPs with reported associations, only rs2236196 was associated in our study (*P*=0.027, Table 3): its minor allele (frequency=0.28) conferring increased risk for nicotine dependence, consistent with prior reports.^76, 82, 83, 85, 86, 87^ rs2236196 shows some linkage disequilibrium with rs2273500 (D′=0.86 and *r*^2^=0.50, Supplementary Figure 3). However, in a model including both SNPs, the association remained for rs2273500 (*P*=2.4 × 10^−5^) but not rs2236196 (*P*=0.79) in meta-analysis across our GWAS samples, indicating that our signal is distinct from the previously reported SNP association. rs2273500 was presented in one of our prior reports as having a suggestive, but nonsignificant, association with nicotine dependence (*N*=1929, *P*=0.081).^82^ By using the multidimensional FTND measure of physiological dependence paired with 1000 Genomes imputation in a large sample, the current study provides the first genome-wide level of significant evidence supporting specific *CHRNA4* variant associations with nicotine dependence, which were not observed in prior studies of CPD with larger sample sizes. Such phenotype differences have similarly been observed for the established *CHRNB3* region, whereby genome-wide significant variants were identified when using FTND-defined nicotine dependence but not when using CPD.^9^

Rare variants in *CHRNA4* have also been implicated as contributing to nicotine dependence risk. A rare missense variant allele in exon 5, R336C (rs56175056), which lowers the sensitivity of the α4 receptor to nicotine exposure,^90^ has been associated with increased risks of nicotine dependence and smoking-related diseases, including lung cancer, chronic obstructive pulmonary disease, peripheral artery disease and abdominal aortic aneurysms.^91^ Another rare variant in exon 5, P451L (rs55915440), was nominated for its association with decreased risk of nicotine dependence^92^ but not independently corroborated.^91^ rs2273500 is located 5.2 kb from R336C and 5.5 kb from P451L; neither rare variant was captured in our study owing to their frequencies.

Our study of common variants identified a splice site acceptor variant allele (rs2273500-C) as being associated with (1) increased risk of nicotine dependence at genome-wide significance, (2) decreased *CHRNA4* expression in human brain and (3) increased lung cancer risk likely through its effect on smoking. Future studies with a large sample of brain-specific *CHRNA4* exon-specific sequences and FTND measurements are needed to validate and elucidate the splicing mechanism involving rs2273500 and its effect on nicotine dependence risk. Nonetheless, our new evidence revealing a common SNP with important regulatory features, along with a newly discovered functional rare variant,^91^ firmly establish *CHRNA4* as an important susceptibility gene for nicotine dependence and its adverse health consequences.

## Figures and Tables

**Table 1:** Participant characteristics from 10 study samples used for the genome-wide association study (GWAS) meta-analysis or independent replication of follow-up GWAS variants

*Study sample*	*Total N*	*No. (%)*^a^			*No. (%), male*	*Mean age (s.d.)*
		*Mild nicotine dependence*	*Moderate nicotine dependence*	*Severe nicotine dependence*		
*GWAS meta-analysis samples*						
deCODE	9090	5871 (64.6)	2074 (22.8)	1145 (12.6)	4253 (46.8)	54.2 (16.7)
EAGLE	3006	1416 (47.1)	1027 (34.2)	563 (18.7)	2528 (84.1)	Not available^b^
COPDGene	2211	666 (30.1)	964 (43.6)	581 (26.3)	1214 (54.9)	57.7 (7.9)
COGEND	1935	941 (48.6)	521 (26.9)	473 (24.4)	750 (38.8)	36.5 (5.5)
SAGE*	832	243 (29.2)	295 (35.5)	294 (35.3)	465 (55.9)	39.4 (11.3)

*Independent replication samples*						
FTC	2374	1345 (56.7)	793 (33.4)	236 (9.9)	1314 (55.3)	45.9 (15.6)
Yale-Penn	2116	381 (18.0)	1014 (47.9)	721 (34.1)	1247 (58.9)	37.7 (10.3)
UW-TTURC	1534	311 (20.2)	723 (47.1)	500 (32.6)	658 (42.9)	43.1 (11.5)
GAIN	774	327 (42.3)	280 (36.2)	167 (21.6)	389 (50.3)	53.8 (16.4)
nonGAIN	671	298 (44.4)	234 (34.9)	139 (20.7)	349 (52.0)	52.9 (15.5)

Abbreviations: COGEND, Collaborative Genetic Study of Nicotine Dependence; COPDGene, Chronic Obstructive Pulmonary Disease Gene Study; EAGLE, Environment and Genetics in Lung Cancer Etiology Study; FTC, Finnish Twin Cohort Study; GAIN, Genetic Association Information Network GWAS of schizophrenia; nonGAIN, Molecular Genetics of Schizophrenia—nonGAIN sample; SAGE*, Study of Addiction: Genetics and Environment (* indicates that overlapping COGEND participants were excluded); UW-TTURC, University of Wisconsin-Transdisciplinary Tobacco Use Research Center.

a Scores on the Fagerström Test for Nicotine Dependence (FTND) were used to categorize nicotine dependence as mild (FTND score 0–3), moderate (FTND score 4–6) or severe (FTND score 7–10). For deCODE only, the mild category included 1558 participants with FTND score 0–3 and an additional set of 4313 low-intensity smokers with no FTND data available but with less than 10 cigarettes per day reported.

b For EAGLE, age was only available as a categorical variable, so average age could not be calculated. The categorical age distributions were as follows: 23.2% aged 59 or less, 18.2% aged 60–64, 22.4% aged 65–69, 21.4% aged 70–74 and 14.8% aged 75–79.

**Table 2:** *CHRNA4* SNPs and indels associated with nicotine dependence at genome-wide association study (GWAS) meta-analysis *P*<5 × 10^−5^ and followed up for independent replication testing

*SNP/indel*	*Minor allele*	*Base pair position* *(NCBI build 37)*	*SNP type*	*MAF*^a^	*GWAS sample meta-analysis* *(*N*=17 074)*		*Replication sample meta-analysis* *(*N*=7469)*		*All sample* meta-analysis (N*=24 543)*	
					β	P	β	P	β	P
rs2273500	C	61 986 949	Intron	0.15	0.057	2.3 × 10^−6^	0.061	9.2 × 10^−4^	0.058	**8.0 × 10**^**−9**^
rs6011779	C	61 984 317	Intron	0.20	0.049	6.0 × 10^−^^6^	0.059	5.5 × 10^−4^	0.052	**1.4 × 10**^**−8**^
rs6062901	G	61 980 261	Intron	0.18	0.050	7.0 × 10^−^^6^	0.055	2.1 × 10^−3^	0.051	5.2 × 10^−8^
rs6062899	G	61 979 793	Intron	0.19	0.049	9.8 × 10^−^^6^	0.054	2.4 × 10^−3^	0.050	8.6 × 10^−8^
rs4809543	A	61 986 950	Intron	0.074	0.086	3.0 × 10^−7^	0.046	0.070	0.074	1.3 × 10^−7^
rs45449494	G	61 987 930	Intron	0.080	0.079	5.7 × 10^−7^	0.047	0.059	0.070	1.7 × 10^−7^
rs45577732	G	61 983 934	Intron	0.078	0.082	4.6 × 10^−7^	0.045	0.070	0.071	1.9 × 10^−7^
rs201806007	AT	61 988 398	Intron	0.15	0.054	1.2 × 10^−6^	0.054	5.3 × 10^−3^	0.054	2.1 × 10^−7^
rs151176846	C	61 997 500	Intron	0.076	0.083	4.4 × 10^−7^	0.040	0.12	0.071	3.4 × 10^−7^
rs45623037	C	61 989 658	Intron	0.080	0.078	7.2 × 10^−7^	0.043	0.092	0.068	3.5 × 10^−7^
rs4809294	A	61 977 723	3′-UTR	0.055	0.11	**3.8 × 10**^**−8**^	0.027	0.38	0.085	3.8 × 10^−7^
rs4809542	G	61 986 787	Intron	0.067	0.088	3.9 × 10^−7^	0.035	0.19	0.072	7.1 × 10^−7^
rs45618935	A	61 979 347	Intron	0.060	0.099	1.5 × 10^−7^	0.028	0.35	0.079	7.3 × 10^−7^
rs45461993	A	61 983 901	Intron	0.080	0.076	2.1 × 10^−6^	0.044	0.088	0.067	8.4 × 10^−7^
rs4809292	G	61 977 506	3′-UTR	0.061	0.096	2.2 × 10^−7^	0.029	0.35	0.078	8.9 × 10^−7^
rs199666656	T	61 975 634	3′-UTR	0.057	0.094	3.3 × 10^−7^	0.026	0.39	0.075	1.6 × 10^−6^
rs45497800	T	61 991 833	Intron	0.082	0.075	2.2 × 10^−6^	0.035	0.16	0.064	2.0 × 10^−6^
rs45456294	G	61 974 832	3′-UTR	0.062	0.092	6.2 × 10^−7^	0.024	0.43	0.073	3.2 × 10^−6^
rs45508092	G	61 974 731	3′-UTR	0.059	0.096	5.8 × 10^−7^	0.025	0.40	0.076	3.2 × 10^−6^
rs4809293	A	61 977 640	3′-UTR	0.048	0.12	7.2 × 10^−8^	0.010	0.75	0.084	3.4 × 10^−6^
rs45612034	A	61 974 970	3′-UTR	0.062	0.090	1.2 × 10^−6^	0.024	0.42	0.072	5.0 × 10^−6^
rs45470098	A	61 979 328	Intron	0.036	0.14	2.4 × 10^−7^	0.0079	0.82	0.090	2.1 × 10^−5^
rs144298540	T	61 984 931	Intron	0.049	0.10	4.1 × 10^−6^	0.0045	0.88	0.068	1.3 × 10^−^^4^

Abbreviations: indel, insertion/deletion; MAF, minor allele frequency; SNP, single nucleotide polymorphism; UTR, untranslated region.

a MAF was weighted by sample size across all 10 samples.

SNPs and indels are sorted by meta-analysis *P*-values across all the samples. *P*-values surpassing genome-wide significance threshold (*P*<5 × 10^−8^) are in bold.

**Table 3:** Results of previously reported *CHRNA4* SNPs in our genome-wide association study meta-analysis of nicotine dependence

*SNP*	*Base pair position* *(NCBI build 37)*	*Minor allele*	*MAF*^a^	*deCODE (*N*=9090)*			*EAGLE (*N*=3006)*			*COPDGene (*N*=2211)*			*COGEND (*N*=1935)*			*SAGE (*N*=832)*			*Meta-analysis P*
				*Info*	β	P	*Info*	β	P	*Info*	β	P	*Info*	β	P	*Info*	β	P	
rs2236196	61 977 556	G	0.28	0.99	0.0075	0.53	0.87	0.014	0.52	0.94	0.029	0.25	0.92	0.089	3.4 × 10^−3^	0.95	0.065	0.12	0.027
rs2229959	61 981 554	C	0.11	0.99	0.010	0.58	0.96	0.0032	0.91	0.98	−0.00097	0.98	0.92	0.11	6.9 × 10^−3^	0.98	0.052	0.33	0.16
rs2273504	61 988 061	A	0.17	0.98	−0.019	0.17	0.81	0.037	0.21	0.92	0.025	0.42	0.89	−0.062	0.079	0.90	−0.15	7.0 × 10^−3^	0.17
rs2273505	61 990 878	T	0.066	0.99	0.012	0.61	0.89	0.0066	0.85	0.96	0.018	0.68	0.87	0.11	0.031	0.94	0.025	0.73	0.19
rs1044396	61 981 134	G	0.46	0.99	−0.0035	0.74	0.88	0.032	0.12	1	0.038	0.088	1	0.029	0.26	1	−0.028	0.46	0.29
rs1044397	61 981 104	C	0.46	0.99	−0.0054	0.61	0.88	0.037	0.068	0.99	0.035	0.11	0.99	0.028	0.28	0.99	−0.023	0.55	0.32
rs3787137	61 979 100	G	0.45	0.99	−0.0074	0.48	0.89	0.035	0.084	0.98	0.036	0.11	0.98	0.032	0.23	0.98	−0.026	0.50	0.40
rs6122429	61 993 206	T	0.13	0.99	−0.00083	0.96	0.91	0.0056	0.83	0.96	−0.0029	0.93	0.92	0.048	0.21	0.94	−0.018	0.75	0.69
rs1044394	61 982 085	A	0.063	0.99	0.0020	0.93	0.84	0.0071	0.86	1	−0.029	0.52	1	0.052	0.35	1	0.041	0.56	0.77

Abbreviations: COGEND, Collaborative Genetic Study of Nicotine Dependence; COPDGene, Chronic Obstructive Pulmonary Disease Gene Study; EAGLE, Environment and Genetics in Lung Cancer Etiology Study; MAF, minor allele frequency; SAGE, Study of Addiction: Genetics and Environment; SNP, single nucleotide polymorphism.

a MAF was weighted by sample size across the five samples.

Among studies testing for *CHRNA4* SNP associations with nicotine dependence or other smoking-related behaviors, these SNPs were reported in at least one of the cited reference studies as having a nominally or statistically significant association. SNPs are sorted by the meta-analysis *P*-value from our study.

## Supplementary Material

Supplementyary Information
