# Supplementary material for: Genome-wide meta-analysis reveals common splice site acceptor variant in CHRNA4 associated with nicotine dependence
Source: Transl Psychiatry. 2015 Oct 6;5(10):e651–. doi: 10.1038/tp.2015.149 (PMC4930126; doi:10.1038/tp.2015.149)
Supplement: Supplementyary Information [file tp2015149x1.docx]

**Supplementary Information**

**Standard quality control (QC) procedures.** For all studies obtained from the database of Genotypes and Phenotypes (dbGaP), we began by applying all participant-level and SNP-level QC procedures that were recommended as part of the dbGaP release. We then applied our own standard set of QC procedures to remove participants with a missing rate >3%, sample duplication (identity-by-state >90%), first-degree relatedness (identity-by-descent >40%), gender discordance (FST <0.2 for chromosome X SNPs to confirm females and FST >0.8 to confirm males), excessive homozygosity (FST >0.5 or FST <-0.2), or chromosomal anomalies and to remove any SNPs with a missing rate >3% or Hardy-Weinberg equilibrium (HWE) P<1×10-4.

**1000 Genomes imputation.** Genotype imputation was conducted in each U.S.-based sample using IMPUTE2[^25^](#_ENREF_25) with reference to the 1000 Genomes ALL phase I integrated variant set (released on December 9, 2013 at https://mathgen.stats.ox.ac.uk/impute/data_download_1000G_phase1_integrated_SHAPEIT2_9-12-13.html). This panel contains 36,820,992 SNPs and 1,384,273 short bi-allelic insertions/deletions (indels) available across all 1000 Genomes populations. Genotype imputation was preceded by pre-phasing the study genotypes using SHAPEIT2[^26^](#_ENREF_26) with default settings applied, except for the following: 500 conditioning states to improve the accuracy of phasing; an effective population size of 11,418, which is recommended for populations of European-ancestry; 10 burn-in iterations; 10 pruning iterations; and 50 iterations for the algorithm to compute transition probabilities in the haplotype graphs. The resulting haplotype estimates were used as the basis for imputation on 5 MB chromosomal chunks and 1 MB flanking buffers with default IMPUTE2 settings applied, except for specifying the expected number of most useful reference haplotypes as 170 (number of CEU haplotypes).

**Assessing imputation quality for rs2273500.** To assess the fidelity of imputation for our lead *CHRNA4* SNP rs2273500, we compared its observed genotypes and imputed dosages using a subset of the COGEND discovery sample. Rs2273500 was genotyped on the Illumina 2.5M array in this subset (N=920); the remainder of the COGEND sample (N=1,015) was genotyped on the Illumina 1M array. To circumvent bias that may arise from conducting imputation on subjects genotyped on different arrays, we carried forward only the SNPs genotyped across both arrays. In the 920 COGEND subjects with Illumina 2.5M array genotyping, we observed high agreement (R^2^=0.83) between the rs2273500 genotypes and its imputation dosages that were based on the overlapping 1M-2.5M SNP genotype set with rs2273500 blinded.

Our categorical FTND regression models were repeated in this COGEND subset (N=920) to compare the nicotine dependence association results when using the observed genotypes vs. imputed dosages. Although neither model was significantly associated likely due to the reduced sample size and reduced statistical power, the magnitude for rs2273500 association with nicotine dependence was stronger when using observed genotypes (β=0.047, standard error=0.040, and *P*=0.24) compared to using imputed dosages (β=0.028, standard error=0.042, and *P*=0.50), suggesting that the rs2273500 association was underestimated in the genome-wide association study of COGEND and possibly the other samples that relied on rs2273500 imputation.

**Selection of sample-specific covariates for nicotine dependence association testing**. Besides age, sex, and principal component eigenvectors, other sample-specific covariates were included due to the original study’s ascertainment scheme. The SAGE* sample, which was ascertained based on addictions other than nicotine, included two additional covariates to adjust for DSM-IV-defined alcohol dependence and cocaine dependence. The EAGLE and COPDGene samples were ascertained based on smoking-related clinical outcomes. EAGLE was ascertained for lung cancer, but FTND data were collected based on lifetime smoking habits among current and former smokers. Adjustments for lung cancer case/control status and current/former smoking were not included in the primary analyses in EAGLE, but *post hoc* analyses with adjustment for these statuses showed that the direction and magnitude of our most significant SNP association with nicotine dependence were consistent with the primary result (results not shown). In contrast, COPDGene had FTND data collected based on current smoking status among COPD cases and controls. Since their current cigarette smoking behavior may be influenced by the onset and progression of lung disease, the regression models used to conduct GWAS analyses in COPDGene were additionally adjusted for severity of COPD, coded as three dummy variables corresponding to GOLD stages of 0, 1-2, and 3-4.

**Supplementary Table 1.** Chromosome 20q13 SNP and indel associations with nicotine dependence at meta-analysis *P*<5×10^-5^. Results were taken from a linear regression model in each sample with categorical nicotine dependence (mild, moderate, and severe) as the outcome, SNP/indel genotype dosage as the predictor, and age, sex, principal component eigenvectors (if applicable), and other sample-specific variables (if applicable) as covariates. The sample-specific results following correction for genomic control are shown. SNPs/indels are sorted by the meta-analysis *P* values, and the genome-wide significant *P* value (*P*<5**×**10^-8^) is bolded.

| **SNP / indel** | **Base pair position (NCBI build 37)** | **Minor allele** | **MAF^1^** | **deCODE (N=9,090)** | | | **EAGLE (N=3,006)** | | | **COPDGene (N=2,211)** | | | **COGEND (N=1,935)** | | | **SAGE (N=832)** | | | **Meta-analysis *P*** |
| --- | --- | --- | --- | --- | --- | --- | --- | --- | --- | --- | --- | --- | --- | --- | --- | --- | --- | --- | --- |
|  |  |  |  | **info** | **β** | ***P*** | **info** | **β** | ***P*** | **info** | **β** | ***P*** | **info** | **β** | ***P*** | **info** | **β** | ***P*** |  |
| rs4809294 | 61977723 | A | 0.049 | 0.99 | 0.12 | 3.5×10^-6^ | 0.78 | 0.051 | 0.31 | 0.87 | 0.13 | 0.026 | 0.82 | 0.10 | 0.16 | 0.85 | 0.18 | 0.083 | **3.8×10^-8^** |
| rs4809293 | 61977640 | A | 0.043 | 0.99 | 0.12 | 3.2×10^-5^ | 0.76 | 0.052 | 0.33 | 0.85 | 0.15 | 0.013 | 0.80 | 0.13 | 0.070 | 0.84 | 0.21 | 0.046 | 7.2×10^-8^ |
| rs45618935 | 61979347 | A | 0.057 | 0.97 | 0.10 | 1.9×10^-5^ | 0.78 | 0.046 | 0.36 | 0.87 | 0.14 | 0.016 | 0.82 | 0.09 | 0.21 | 0.86 | 0.19 | 0.058 | 1.5×10^-7^ |
| rs4809292 | 61977506 | G | 0.058 | 0.99 | 0.096 | 2.7×10^-5^ | 0.78 | 0.045 | 0.36 | 0.87 | 0.14 | 0.015 | 0.82 | 0.08 | 0.22 | 0.86 | 0.19 | 0.060 | 2.2×10^-7^ |
| rs45470098 | 61979328 | A | 0.028 | 0.99 | 0.14 | 2.7×10^-4^ | 0.79 | 0.067 | 0.24 | 0.86 | 0.16 | 0.017 | 0.83 | 0.19 | 0.019 | 0.86 | 0.21 | 0.065 | 2.4×10^-7^ |
| rs4809543 | 61986950 | A | 0.071 | 0.99 | 0.093 | 1.8×10^-5^ | 0.77 | 0.071 | 0.11 | 0.90 | 0.075 | 0.11 | 0.84 | 0.07 | 0.17 | 0.87 | 0.088 | 0.28 | 3.0×10^-7^ |
| rs199666656 | 61975634 | T | 0.059 | 0.99 | 0.097 | 3.2×10^-5^ | 0.78 | 0.038 | 0.42 | 0.87 | 0.14 | 0.012 | 0.82 | 0.08 | 0.22 | 0.86 | 0.17 | 0.085 | 3.3×10^-7^ |
| rs4809542 | 61986787 | G | 0.065 | 0.99 | 0.093 | 2.6×10^-5^ | 0.76 | 0.063 | 0.19 | 0.89 | 0.089 | 0.073 | 0.83 | 0.07 | 0.20 | 0.86 | 0.12 | 0.15 | 3.9×10^-7^ |
| rs151176846 | 61997500 | C | 0.073 | 0.98 | 0.090 | 2.1×10^-5^ | 0.79 | 0.063 | 0.16 | 0.88 | 0.087 | 0.065 | 0.83 | 0.05 | 0.33 | 0.86 | 0.11 | 0.18 | 4.4×10^-7^ |
| rs45577732 | 61983934 | G | 0.074 | 0.99 | 0.091 | 1.6×10^-5^ | 0.77 | 0.064 | 0.14 | 0.90 | 0.081 | 0.079 | 0.84 | 0.06 | 0.24 | 0.86 | 0.073 | 0.37 | 4.6×10^-7^ |
| rs45449494 | 61987930 | G | 0.079 | 0.98 | 0.082 | 4.4×10^-5^ | 0.77 | 0.067 | 0.12 | 0.90 | 0.081 | 0.076 | 0.85 | 0.07 | 0.21 | 0.87 | 0.095 | 0.23 | 5.7×10^-7^ |
| rs45508092 | 61974731 | G | 0.054 | 0.98 | 0.10 | 3.3×10^-5^ | 0.78 | 0.032 | 0.51 | 0.88 | 0.13 | 0.018 | 0.84 | 0.09 | 0.19 | 0.87 | 0.16 | 0.10 | 5.8×10^-7^ |
| rs45456294 | 61974832 | G | 0.058 | 0.98 | 0.095 | 4.3×10^-5^ | 0.78 | 0.037 | 0.44 | 0.88 | 0.14 | 0.014 | 0.84 | 0.08 | 0.25 | 0.87 | 0.16 | 0.10 | 6.2×10^-7^ |
| rs45623037 | 61989658 | C | 0.080 | 0.98 | 0.081 | 5.9×10^-5^ | 0.77 | 0.069 | 0.12 | 0.90 | 0.081 | 0.078 | 0.84 | 0.07 | 0.21 | 0.87 | 0.096 | 0.23 | 7.2×10^-7^ |
| rs45612034 | 61974970 | A | 0.058 | 0.98 | 0.091 | 8.4×10^-5^ | 0.78 | 0.037 | 0.44 | 0.88 | 0.14 | 0.014 | 0.83 | 0.08 | 0.24 | 0.86 | 0.16 | 0.10 | 1.2×10^-6^ |
| rs45461993 | 61983901 | A | 0.078 | 0.98 | 0.077 | 1.8×10^-4^ | 0.78 | 0.065 | 0.14 | 0.91 | 0.082 | 0.072 | 0.85 | 0.07 | 0.21 | 0.88 | 0.097 | 0.22 | 2.1×10^-6^ |
| rs45497800 | 61991833 | T | 0.080 | 0.98 | 0.074 | 2.1×10^-4^ | 0.78 | 0.069 | 0.12 | 0.88 | 0.088 | 0.053 | 0.84 | 0.06 | 0.26 | 0.85 | 0.096 | 0.23 | 2.2×10^-6^ |
| rs2273500 | 61986949 | C | 0.15 | 0.99 | 0.058 | 3.4×10^-4^ | 0.85 | 0.035 | 0.24 | 0.94 | 0.050 | 0.12 | 0.90 | 0.09 | 0.016 | 0.92 | 0.069 | 0.23 | 2.3×10^-6^ |
| rs144298540 | 61984931 | T | 0.044 | 0.83 | 0.095 | 5.9×10^-4^ | 0.71 | 0.10 | 0.088 | 0.82 | 0.16 | 0.017 | 0.75 | 0.06 | 0.45 | 0.79 | 0.13 | 0.26 | 4.1×10^-6^ |
| rs6011779 | 61984317 | C | 0.19 | 0.98 | 0.049 | 8.6×10^-4^ | 0.90 | 0.024 | 0.35 | 0.96 | 0.035 | 0.22 | 0.91 | 0.10 | 0.0038 | 0.94 | 0.075 | 0.12 | 6.0×10^-6^ |
| rs6062901 | 61980261 | G | 0.17 | 0.99 | 0.049 | 0.0012 | 0.91 | 0.020 | 0.46 | 0.95 | 0.036 | 0.24 | 0.90 | 0.12 | 8.5×10^-4^ | 0.95 | 0.082 | 0.11 | 7.0×10^-6^ |
| rs6062899 | 61979793 | G | 0.18 | 0.99 | 0.046 | 0.0017 | 0.92 | 0.019 | 0.46 | 0.94 | 0.036 | 0.24 | 0.90 | 0.13 | 8.5×10^-4^ | 0.95 | 0.082 | 0.11 | 9.8×10^-6^ |
| rs201806007 | 61988398 | AT | 0.15 | 0.88 | 0.050 | 0.0030 | 0.80 | 0.028 | 0.33 | 0.89 | 0.045 | 0.16 | 0.86 | 0.11 | 0.0047 | 0.87 | 0.096 | 0.090 | 1.2×10^-5^ |

MAF, minor allele frequency.

^1^MAF was weighted by sample size across the five samples.

**Supplementary Table 2.** Chromosome 15q25 SNP and indel associations with nicotine dependence at meta-analysis *P*<5×10^-5^. Results were taken from a linear regression model in each sample with categorical nicotine dependence (mild, moderate, and severe) as the outcome, SNP/indel genotype dosage as the predictor, and age, sex, principal component eigenvectors (if applicable), and other sample-specific variables (if applicable) as covariates. The sample-specific results following correction for genomic control are shown. SNPs/indels are sorted by the meta-analysis *P* values, and the genome-wide significant *P* values (*P*<5**×**10^-8^) are bolded.

| **SNP /**  **indel** | **Base pair position (NCBI build 37)** | **Minor allele** | **MAF^1^** | **deCODE (N=9,090)** | | | **EAGLE (N=3,006)** | | | **COPDGene (N=2,211)** | | | **COGEND (N=1,935)** | | | **SAGE (N=832)** | | | **Meta-analysis P** |
| --- | --- | --- | --- | --- | --- | --- | --- | --- | --- | --- | --- | --- | --- | --- | --- | --- | --- | --- | --- |
|  |  |  |  | **info** | **β** | **P** | **info** | **β** | **P** | **info** | **Β** | **P** | **info** | **β** | **P** | **info** | **Β** | **P** |  |
| rs34684276 | 78813155 | A | 0.35 | 1.00 | 0.067 | 1.3×10^-8^ | 0.97 | 0.088 | 4.6×10^-5^ | 0.98 | 0.078 | 0.0018 | 0.98 | 0.11 | 3.3×10^-4^ | 0.98 | 0.052 | 0.21 | **3.5×10^-17^** |
| rs114205691 | 78901113 | T | 0.36 | 1.00 | 0.068 | 8.5×10^-9^ | 1.00 | 0.073 | 6.1×10^-4^ | 1.00 | 0.072 | 0.0037 | 1.00 | 0.13 | 1.2×10^-5^ | 1.00 | 0.057 | 0.16 | **3.8×10^-17^** |
| rs8192482 | 78886198 | T | 0.36 | 1.00 | 0.069 | 7.1×10^-9^ | 1.00 | 0.072 | 7.3×10^-4^ | 1.00 | 0.072 | 0.0037 | 1.00 | 0.12 | 1.9×10^-5^ | 1.00 | 0.061 | 0.13 | **4.1×10^-17^** |
| rs4887067 | 78886947 | A | 0.36 | 1.00 | 0.068 | 7.3×10^-9^ | 1.00 | 0.072 | 7.2×10^-4^ | 1.00 | 0.072 | 0.0037 | 1.00 | 0.12 | 2.0×10^-5^ | 1.00 | 0.061 | 0.13 | **4.2×10^-17^** |
| rs1051730 | 78894339 | A | 0.36 | 1.00 | 0.068 | 8.4×10^-9^ | 1.00 | 0.074 | 4.8×10^-4^ | 1.00 | 0.069 | 0.0050 | 1.00 | 0.12 | 1.8×10^-5^ | 1.00 | 0.061 | 0.13 | **4.3×10^-17^** |
| rs55676755 | 78898932 | G | 0.36 | 1.00 | 0.068 | 9.1×10^-9^ | 1.00 | 0.073 | 5.9×10^-4^ | 1.00 | 0.071 | 0.0039 | 1.00 | 0.12 | 1.3×10^-5^ | 1.00 | 0.056 | 0.16 | **4.6×10^-17^** |
| rs16969968 | 78882925 | A | 0.36 | 1.00 | 0.068 | 8.9×10^-9^ | 0.99 | 0.072 | 7.5×10^-4^ | 1.00 | 0.072 | 0.0039 | 1.00 | 0.12 | 1.6×10^-5^ | 1.00 | 0.061 | 0.13 | **5.1×10^-17^** |
| rs138544659 | 78900701 | G | 0.35 | 1.00 | 0.068 | 9.8×10^-9^ | 0.95 | 0.080 | 2.7×10^-4^ | 0.96 | 0.071 | 0.0067 | 0.96 | 0.13 | 1.7×10^-5^ | 0.95 | 0.057 | 0.18 | **5.9×10^-17^** |
| rs147144681 | 78900908 | T | 0.35 | 1.00 | 0.068 | 9.8×10^-9^ | 0.95 | 0.080 | 2.6×10^-4^ | 0.96 | 0.070 | 0.0073 | 0.96 | 0.13 | 1.6×10^-5^ | 0.96 | 0.054 | 0.19 | **6.7×10^-17^** |
| rs141518190 | 78900647 | G | 0.35 | 1.00 | 0.068 | 9.6×10^-9^ | 0.94 | 0.077 | 5.1×10^-4^ | 0.95 | 0.074 | 0.0048 | 0.95 | 0.13 | 1.5×10^-5^ | 0.95 | 0.052 | 0.22 | **9.4×10^-17^** |
| rs147499554 | 78900650 | T | 0.35 | 1.00 | 0.068 | 9.7×10^-9^ | 0.94 | 0.077 | 5.1×10^-4^ | 0.95 | 0.074 | 0.0048 | 0.95 | 0.13 | 1.5×10^-5^ | 0.95 | 0.052 | 0.22 | **9.5×10^-17^** |
| rs146009840 | 78906177 | T | 0.36 | 1.00 | 0.067 | 1.4×10^-8^ | 1.00 | 0.072 | 7.1×10^-4^ | 1.00 | 0.069 | 0.0055 | 0.99 | 0.12 | 3.2×10^-5^ | 0.99 | 0.065 | 0.11 | **1.3×10^-16^** |
| rs951266 | 78878541 | A | 0.36 | 1.00 | 0.067 | 2.0×10^-8^ | 0.99 | 0.076 | 3.7×10^-4^ | 1.00 | 0.067 | 0.0071 | 1.00 | 0.12 | 1.8×10^-5^ | 1.00 | 0.058 | 0.15 | **1.4×10^-16^** |
| rs7180002 | 78873993 | T | 0.36 | 1.00 | 0.067 | 1.8×10^-8^ | 0.99 | 0.076 | 4.0×10^-4^ | 1.00 | 0.066 | 0.0079 | 1.00 | 0.12 | 1.8×10^-5^ | 1.00 | 0.057 | 0.16 | **1.6×10^-16^** |
| rs56390833 | 78877381 | A | 0.36 | 1.00 | 0.067 | 2.0×10^-8^ | 0.99 | 0.076 | 3.8×10^-4^ | 1.00 | 0.066 | 0.0079 | 1.00 | 0.12 | 1.8×10^-5^ | 1.00 | 0.058 | 0.15 | **1.6×10^-16^** |
| rs11633958 | 78862064 | T | 0.36 | 1.00 | 0.067 | 1.4×10^-8^ | 0.99 | 0.073 | 6.9×10^-4^ | 1.00 | 0.068 | 0.0060 | 1.00 | 0.12 | 1.6×10^-5^ | 1.00 | 0.053 | 0.19 | **1.7×10^-16^** |
| rs17486195 | 78865197 | G | 0.36 | 1.00 | 0.067 | 1.8×10^-8^ | 0.99 | 0.075 | 4.1×10^-4^ | 1.00 | 0.066 | 0.0079 | 1.00 | 0.12 | 1.8×10^-5^ | 1.00 | 0.055 | 0.17 | **1.7×10^-16^** |
| rs140330585 | 78866445 | A | 0.36 | 1.00 | 0.067 | 1.9×10^-8^ | 0.99 | 0.075 | 4.1×10^-4^ | 1.00 | 0.066 | 0.0079 | 1.00 | 0.12 | 1.8×10^-5^ | 1.00 | 0.055 | 0.17 | **1.8×10^-16^** |
| rs1317286 | 78896129 | G | 0.36 | 1.00 | 0.066 | 2.0×10^-8^ | 1.00 | 0.074 | 5.2×10^-4^ | 1.00 | 0.070 | 0.0048 | 1.00 | 0.12 | 3.1×10^-5^ | 1.00 | 0.056 | 0.16 | **1.9×10^-16^** |
| rs56077333 | 78899003 | A | 0.35 | 1.00 | 0.069 | 5.2×10^-9^ | 0.95 | 0.077 | 5.1×10^-4^ | 0.96 | 0.061 | 0.018 | 0.96 | 0.13 | 9.7×10^-6^ | 0.96 | 0.043 | 0.30 | **1.9×10^-16^** |
| rs72740964 | 78868636 | A | 0.36 | 1.00 | 0.067 | 1.8×10^-8^ | 0.99 | 0.072 | 7.0×10^-4^ | 1.00 | 0.068 | 0.0060 | 1.00 | 0.12 | 1.6×10^-5^ | 1.00 | 0.056 | 0.17 | **2.0×10^-16^** |
| rs7172118 | 78862453 | A | 0.36 | 1.00 | 0.067 | 2.0×10^-8^ | 0.99 | 0.076 | 4.0×10^-4^ | 1.00 | 0.066 | 0.0079 | 1.00 | 0.12 | 1.8×10^-5^ | 1.00 | 0.054 | 0.18 | **2.0×10^-16^** |
| rs17486278 | 78867482 | C | 0.36 | 1.00 | 0.066 | 3.2×10^-8^ | 0.99 | 0.074 | 5.0×10^-4^ | 1.00 | 0.068 | 0.0059 | 1.00 | 0.12 | 1.7×10^-5^ | 1.00 | 0.056 | 0.16 | **2.5×10^-16^** |
| rs8034191 | 78806023 | C | 0.36 | 1.00 | 0.066 | 2.7×10^-8^ | 1.00 | 0.082 | 1.0×10^-4^ | 1.00 | 0.064 | 0.0089 | 1.00 | 0.11 | 7.6×10^-5^ | 1.00 | 0.046 | 0.26 | **3.2×10^-16^** |
| rs55853698 | 78857939 | G | 0.36 | 0.99 | 0.065 | 5.1×10^-8^ | 0.98 | 0.079 | 2.4×10^-4^ | 0.99 | 0.070 | 0.0051 | 0.99 | 0.12 | 2.3×10^-5^ | 0.99 | 0.048 | 0.24 | **3.4×10^-16^** |
| rs72740955 | 78849779 | T | 0.36 | 1.00 | 0.065 | 4.2×10^-8^ | 0.98 | 0.081 | 1.5×10^-4^ | 1.00 | 0.067 | 0.0067 | 1.00 | 0.12 | 3.7×10^-5^ | 1.00 | 0.043 | 0.29 | **4.0×10^-16^** |
| rs2036527 | 78851615 | A | 0.36 | 1.00 | 0.065 | 4.3×10^-8^ | 0.98 | 0.081 | 1.5×10^-4^ | 1.00 | 0.066 | 0.0078 | 1.00 | 0.12 | 3.5×10^-5^ | 1.00 | 0.043 | 0.29 | **4.7×10^-16^** |
| rs9788721 | 78802869 | C | 0.37 | 1.00 | 0.065 | 3.0×10^-8^ | 0.99 | 0.083 | 8.3×10^-5^ | 0.99 | 0.064 | 0.0091 | 1.00 | 0.11 | 2.0×10^-4^ | 0.99 | 0.047 | 0.24 | **5.2×10^-16^** |
| rs11852372 | 78801394 | C | 0.36 | 0.99 | 0.067 | 1.9×10^-8^ | 0.97 | 0.082 | 1.5×10^-4^ | 0.97 | 0.064 | 0.011 | 0.97 | 0.11 | 1.8×10^-4^ | 0.97 | 0.047 | 0.26 | **6.8×10^-16^** |
| rs8031948 | 78816057 | T | 0.36 | 1.00 | 0.065 | 4.6×10^-8^ | 0.99 | 0.081 | 1.3×10^-4^ | 1.00 | 0.067 | 0.0062 | 1.00 | 0.11 | 9.9×10^-5^ | 1.00 | 0.041 | 0.31 | **7.0×10^-16^** |
| rs4243084 | 78911672 | C | 0.37 | 1.00 | 0.064 | 6.1×10^-8^ | 0.99 | 0.074 | 4.7×10^-4^ | 1.00 | 0.067 | 0.0066 | 1.00 | 0.12 | 2.8×10^-5^ | 1.00 | 0.058 | 0.15 | **7.4×10^-16^** |
| rs55781567 | 78857986 | G | 0.36 | 0.99 | 0.063 | 1.2×10^-7^ | 0.98 | 0.080 | 1.7×10^-4^ | 0.99 | 0.067 | 0.0070 | 0.99 | 0.12 | 2.5×10^-5^ | 0.99 | 0.048 | 0.24 | **9.7×10^-16^** |
| rs72738786 | 78828086 | T | 0.36 | 1.00 | 0.065 | 3.9×10^-8^ | 1.00 | 0.080 | 1.7×10^-4^ | 1.00 | 0.067 | 0.0061 | 1.00 | 0.11 | 1.5×10^-4^ | 1.00 | 0.039 | 0.33 | **1.0×10^-15^** |
| rs58365910 | 78849034 | C | 0.36 | 1.00 | 0.063 | 9.9×10^-8^ | 0.98 | 0.083 | 1.2×10^-4^ | 1.00 | 0.066 | 0.0073 | 1.00 | 0.12 | 4.6×10^-5^ | 1.00 | 0.043 | 0.29 | **1.0×10^-15^** |
| rs931794 | 78826180 | G | 0.37 | 1.00 | 0.064 | 6.9×10^-8^ | 1.00 | 0.083 | 9.9×10^-5^ | 1.00 | 0.064 | 0.0086 | 1.00 | 0.10 | 3.5×10^-4^ | 1.00 | 0.043 | 0.28 | **2.4×10^-15^** |
| rs10519203 | 78814046 | G | 0.37 | 1.00 | 0.064 | 7.9×10^-8^ | 0.99 | 0.081 | 1.5×10^-4^ | 1.00 | 0.066 | 0.0072 | 1.00 | 0.11 | 1.7×10^-4^ | 1.00 | 0.039 | 0.33 | **2.4×10^-15^** |
| rs8042849 | 78817929 | C | 0.37 | 1.00 | 0.063 | 8.4×10^-8^ | 0.98 | 0.087 | 4.7×10^-5^ | 0.99 | 0.062 | 0.011 | 0.99 | 0.10 | 4.7×10^-4^ | 0.98 | 0.033 | 0.41 | **4.0×10^-15^** |
| rs139043337 | 78734588 | A | 0.36 | 1.00 | 0.065 | 3.7×10^-8^ | 1.00 | 0.072 | 4.1×10^-4^ | 1.00 | 0.052 | 0.027 | 1.00 | 0.10 | 5.2×10^-4^ | 1.00 | 0.063 | 0.11 | **7.3×10^-15^** |
| rs201844830 | 78751682 | GT | 0.36 | 1.00 | 0.064 | 4.2×10^-8^ | 0.99 | 0.072 | 4.6×10^-4^ | 1.00 | 0.052 | 0.026 | 0.99 | 0.10 | 3.4×10^-4^ | 1.00 | 0.054 | 0.18 | **1.0×10^-14^** |
| rs199642525 | 78901173 | G | 0.25 | 0.99 | 0.068 | 6.7×10^-9^ | 0.71 | 0.14 | 5.7×10^-5^ | 0.76 | 0.097 | 0.0084 | 0.75 | 0.094 | 0.041 | 0.76 | 0.11 | 0.080 | **1.1×10^-14^** |
| rs2009746 | 78754102 | G | 0.36 | 1.00 | 0.066 | 4.0×10^-8^ | 0.99 | 0.074 | 5.6×10^-4^ | 0.99 | 0.055 | 0.028 | 0.99 | 0.10 | 8.5×10^-4^ | 1.00 | 0.062 | 0.14 | **1.7×10^-14^** |
| rs72738736 | 78765122 | T | 0.36 | 1.00 | 0.065 | 4.2×10^-8^ | 0.99 | 0.074 | 5.9×10^-4^ | 0.99 | 0.055 | 0.027 | 0.99 | 0.10 | 8.6×10^-4^ | 1.00 | 0.062 | 0.14 | **1.9×10^-14^** |
| rs1504550 | 78766250 | G | 0.36 | 1.00 | 0.065 | 5.9×10^-8^ | 0.98 | 0.074 | 6.4×10^-4^ | 0.99 | 0.055 | 0.026 | 0.99 | 0.11 | 3.0×10^-4^ | 1.00 | 0.054 | 0.20 | **2.0×10^-14^** |
| rs55958997 | 78915872 | A | 0.40 | 1.00 | 0.058 | 4.3×10^-7^ | 0.99 | 0.071 | 8.6×10^-4^ | 0.99 | 0.072 | 0.0030 | 0.99 | 0.11 | 1.7×10^-4^ | 0.99 | 0.049 | 0.22 | **2.2×10^-14^** |
| rs17483686 | 78733390 | T | 0.36 | 1.00 | 0.065 | 4.8×10^-8^ | 1.00 | 0.072 | 8.0×10^-4^ | 1.00 | 0.052 | 0.036 | 1.00 | 0.10 | 6.6×10^-4^ | 1.00 | 0.063 | 0.13 | **3.0×10^-14^** |
| rs55983731 | 78735269 | T | 0.36 | 1.00 | 0.065 | 4.9×10^-8^ | 1.00 | 0.072 | 7.9×10^-4^ | 1.00 | 0.052 | 0.037 | 1.00 | 0.10 | 7.1×10^-4^ | 1.00 | 0.063 | 0.13 | **3.2×10^-14^** |
| rs72738718 | 78735438 | C | 0.36 | 1.00 | 0.065 | 4.9×10^-8^ | 1.00 | 0.072 | 7.9×10^-4^ | 1.00 | 0.052 | 0.037 | 1.00 | 0.10 | 7.1×10^-4^ | 1.00 | 0.063 | 0.13 | **3.2×10^-14^** |
| rs17483721 | 78733731 | C | 0.36 | 1.00 | 0.065 | 5.6×10^-8^ | 1.00 | 0.072 | 8.0×10^-4^ | 1.00 | 0.052 | 0.036 | 1.00 | 0.10 | 6.6×10^-4^ | 1.00 | 0.063 | 0.13 | **3.5×10^-14^** |
| rs8039449 | 78914534 | T | 0.40 | 1.00 | 0.058 | 3.8×10^-7^ | 0.99 | 0.070 | 9.4×10^-4^ | 0.99 | 0.069 | 0.0047 | 0.99 | 0.10 | 2.4×10^-4^ | 0.99 | 0.050 | 0.21 | **3.8×10^-14^** |
| rs56219465 | 78742579 | G | 0.36 | 1.00 | 0.065 | 4.7×10^-8^ | 1.00 | 0.072 | 7.4×10^-4^ | 1.00 | 0.052 | 0.037 | 1.00 | 0.10 | 9.2×10^-4^ | 1.00 | 0.061 | 0.14 | **3.8×10^-14^** |
| rs7181486 | 78741618 | C | 0.36 | 1.00 | 0.065 | 4.8×10^-8^ | 1.00 | 0.072 | 7.5×10^-4^ | 1.00 | 0.052 | 0.037 | 1.00 | 0.10 | 9.2×10^-4^ | 1.00 | 0.061 | 0.14 | **3.9×10^-14^** |
| rs17484524 | 78772676 | G | 0.36 | 1.00 | 0.064 | 8.6×10^-8^ | 0.98 | 0.073 | 8.0×10^-4^ | 0.99 | 0.056 | 0.026 | 0.99 | 0.10 | 3.7×10^-4^ | 1.00 | 0.052 | 0.21 | **4.1×10^-14^** |
| rs17483929 | 78742376 | A | 0.36 | 1.00 | 0.065 | 4.8×10^-8^ | 1.00 | 0.072 | 7.4×10^-4^ | 1.00 | 0.050 | 0.041 | 1.00 | 0.10 | 9.2×10^-4^ | 1.00 | 0.061 | 0.14 | **4.3×10^-14^** |
| rs72738732 | 78752188 | G | 0.36 | 1.00 | 0.064 | 6.9×10^-8^ | 0.99 | 0.071 | 9.8×10^-4^ | 1.00 | 0.052 | 0.035 | 1.00 | 0.10 | 3.8×10^-4^ | 1.00 | 0.052 | 0.21 | **5.6×10^-14^** |
| rs11858836 | 78783277 | A | 0.36 | 1.00 | 0.063 | 1.4×10^-7^ | 0.98 | 0.073 | 7.8×10^-4^ | 0.99 | 0.056 | 0.024 | 0.99 | 0.10 | 3.6×10^-4^ | 1.00 | 0.052 | 0.21 | **6.5×10^-14^** |
| rs2089162 | 78739763 | G | 0.36 | 1.00 | 0.064 | 8.3×10^-8^ | 0.99 | 0.070 | 0.0012 | 1.00 | 0.053 | 0.034 | 1.00 | 0.10 | 3.2×10^-4^ | 1.00 | 0.053 | 0.20 | **6.6×10^-14^** |
| rs17483548 | 78730313 | A | 0.36 | 1.00 | 0.064 | 7.0×10^-8^ | 0.99 | 0.069 | 0.0015 | 1.00 | 0.053 | 0.034 | 0.99 | 0.10 | 4.0×10^-4^ | 1.00 | 0.054 | 0.19 | **7.5×10^-14^** |
| rs2656065 | 78750549 | A | 0.36 | 1.00 | 0.065 | 4.0×10^-8^ | 1.00 | 0.074 | 5.7×10^-4^ | 1.00 | 0.047 | 0.055 | 1.00 | 0.086 | 0.0028 | 1.00 | 0.059 | 0.15 | **9.1×10^-14^** |
| rs951985 | 78720923 | G | 0.36 | 0.99 | 0.063 | 1.2×10^-7^ | 0.97 | 0.073 | 8.8×10^-4^ | 0.98 | 0.054 | 0.035 | 0.98 | 0.11 | 1.8×10^-4^ | 0.98 | 0.040 | 0.34 | **9.3×10^-14^** |
| rs2656052 | 78740932 | C | 0.36 | 1.00 | 0.065 | 4.7×10^-8^ | 1.00 | 0.072 | 7.8×10^-4^ | 1.00 | 0.049 | 0.046 | 1.00 | 0.089 | 0.0020 | 1.00 | 0.057 | 0.17 | **1.0×10^-13^** |
| rs2938670 | 78740688 | G | 0.36 | 1.00 | 0.065 | 4.8×10^-8^ | 1.00 | 0.072 | 7.8×10^-4^ | 1.00 | 0.049 | 0.046 | 1.00 | 0.089 | 0.0020 | 1.00 | 0.057 | 0.17 | **1.0×10^-13^** |
| rs2568494 | 78740964 | A | 0.36 | 1.00 | 0.065 | 4.8×10^-8^ | 1.00 | 0.072 | 7.8×10^-4^ | 1.00 | 0.049 | 0.045 | 1.00 | 0.088 | 0.0023 | 1.00 | 0.059 | 0.15 | **1.0×10^-13^** |
| rs12914385 | 78898723 | T | 0.40 | 1.00 | 0.060 | 2.4×10^-7^ | 1.00 | 0.061 | 0.0036 | 1.00 | 0.070 | 0.0035 | 1.00 | 0.095 | 7.9×10^-4^ | 1.00 | 0.051 | 0.19 | **1.1×10^-13^** |
| rs17405217 | 78731149 | T | 0.36 | 1.00 | 0.063 | 1.6×10^-7^ | 0.99 | 0.069 | 0.0014 | 1.00 | 0.053 | 0.034 | 0.99 | 0.10 | 4.0×10^-4^ | 1.00 | 0.054 | 0.19 | **1.7×10^-13^** |
| rs72743158 | 78926445 | C | 0.39 | 1.00 | 0.057 | 7.1×10^-7^ | 0.96 | 0.065 | 0.0024 | 0.97 | 0.061 | 0.013 | 0.97 | 0.11 | 1.5×10^-4^ | 0.97 | 0.060 | 0.14 | **2.3×10^-13^** |
| rs17487223 | 78923987 | T | 0.38 | 1.00 | 0.055 | 1.9×10^-6^ | 0.98 | 0.066 | 0.0020 | 0.98 | 0.063 | 0.010 | 0.98 | 0.11 | 7.6×10^-5^ | 0.98 | 0.052 | 0.20 | **4.3×10^-13^** |
| rs2869548 | 78922638 | A | 0.38 | 1.00 | 0.055 | 2.0×10^-6^ | 0.98 | 0.067 | 0.0019 | 0.98 | 0.061 | 0.013 | 0.98 | 0.11 | 9.1×10^-5^ | 0.98 | 0.052 | 0.20 | **6.1×10^-13^** |
| rs72738704 | 78719832 | C | 0.36 | 1.00 | 0.061 | 3.3×10^-7^ | 0.98 | 0.067 | 0.0022 | 0.99 | 0.058 | 0.021 | 0.98 | 0.094 | 0.0013 | 0.99 | 0.053 | 0.20 | **7.0×10^-13^** |
| rs72736802 | 78719501 | T | 0.37 | 0.99 | 0.062 | 1.3×10^-7^ | 0.98 | 0.068 | 0.0017 | 0.99 | 0.046 | 0.060 | 0.98 | 0.072 | 0.013 | 0.98 | 0.055 | 0.18 | **3.0×10^-12^** |
| rs8040868 | 78911181 | C | 0.42 | 1.00 | 0.053 | 2.9×10^-6^ | 0.99 | 0.061 | 0.0037 | 1.00 | 0.059 | 0.013 | 1.00 | 0.092 | 0.0011 | 1.00 | 0.056 | 0.15 | **5.1×10^-12^** |
| rs10851907 | 78915864 | A | 0.44 | 1.00 | 0.052 | 5.0×10^-6^ | 0.99 | 0.063 | 0.0031 | 0.99 | 0.066 | 0.0055 | 0.99 | 0.079 | 0.0049 | 0.99 | 0.045 | 0.25 | **1.7×10^-11^** |
| rs55988292 | 78936168 | G | 0.41 | 1.00 | 0.053 | 3.5×10^-6^ | 0.97 | 0.058 | 0.0062 | 0.99 | 0.049 | 0.044 | 0.98 | 0.10 | 7.1×10^-4^ | 0.98 | 0.061 | 0.13 | **2.3×10^-11^** |
| rs67426328 | 78934318 | G | 0.41 | 1.00 | 0.053 | 3.8×10^-6^ | 0.98 | 0.057 | 0.0070 | 0.99 | 0.047 | 0.056 | 0.98 | 0.093 | 9.4×10^-4^ | 0.98 | 0.063 | 0.12 | **3.8×10^-11^** |
| rs56117933 | 78832349 | C | 0.23 | 1.00 | 0.070 | 4.0×10^-7^ | 0.94 | 0.030 | 0.21 | 0.95 | 0.085 | 0.0032 | 0.95 | 0.10 | 0.0039 | 0.95 | 0.038 | 0.43 | **1.6×10^-10^** |
| rs200776369 | 78874842 | A | 0.38 | NA | NA | NA | 0.99 | 0.076 | 1.7×10^-4^ | 1.00 | 0.066 | 0.0050 | 1.00 | 0.12 | 1.1×10^-5^ | 1.00 | 0.057 | 0.14 | **2.2×10^-10^** |
| rs149959208 | 78912710 | TGCGCGGGGCAGGGCGACGGGCA | 0.28 | 0.84 | 0.41 | 0.39 | 0.96 | 0.075 | 2.9×10^-4^ | 0.96 | 0.070 | 0.0039 | 0.96 | 0.12 | 1.9×10^-5^ | 0.96 | 0.065 | 0.10 | **2.4×10^-10^** |
| rs190065944 | 78859610 | A | 0.39 | NA | NA | NA | 0.98 | 0.081 | 1.7×10^-4^ | 0.99 | 0.067 | 0.0070 | 0.99 | 0.12 | 2.9×10^-5^ | 0.99 | 0.050 | 0.22 | **8.2×10^-10^** |
| rs1394371 | 78724469 | T | 0.30 | 1.00 | 0.059 | 2.7×10^-6^ | 1.00 | 0.047 | 0.035 | 1.00 | 0.040 | 0.12 | 1.00 | 0.10 | 9.7×10^-4^ | 1.00 | 0.027 | 0.52 | **1.2×10^-9^** |
| rs951984 | 78720915 | A | 0.30 | 1.00 | 0.057 | 4.2×10^-6^ | 0.97 | 0.048 | 0.037 | 0.98 | 0.049 | 0.061 | 0.99 | 0.10 | 6.2×10^-4^ | 0.98 | 0.008 | 0.85 | **1.3×10^-9^** |
| rs518425 | 78883813 | G | 0.28 | 1.00 | -0.042 | 6.1×10^-4^ | 0.99 | -0.061 | 0.012 | 1.00 | -0.061 | 0.020 | 1.00 | -0.13 | 4.1×10^-5^ | 1.00 | -0.067 | 0.11 | **1.8×10^-9^** |
| rs200776054 | 78801393 | AC | 0.37 | NA | NA | NA | 0.96 | 0.081 | 8.3×10^-5^ | 0.96 | 0.061 | 0.011 | 0.97 | 0.11 | 8.8×10^-5^ | 0.97 | 0.046 | 0.25 | **1.9×10^-9^** |
| rs578776 | 78888400 | A | 0.27 | 1.00 | -0.043 | 5.9×10^-4^ | 1.00 | -0.056 | 0.021 | 1.00 | -0.058 | 0.025 | 1.00 | -0.13 | 2.0×10^-5^ | 1.00 | -0.068 | 0.11 | **2.4×10^-9^** |
| rs564585 | 78886227 | G | 0.27 | 1.00 | -0.042 | 6.5×10^-4^ | 1.00 | -0.055 | 0.025 | 1.00 | -0.056 | 0.032 | 1.00 | -0.13 | 2.0×10^-5^ | 1.00 | -0.071 | 0.094 | **3.6×10^-9^** |
| rs71852607 | 78799060 | ACT | 0.38 | 1.00 | -0.039 | 5.6×10^-4^ | 0.96 | -0.057 | 0.0072 | 0.97 | -0.062 | 0.0059 | 0.97 | -0.072 | 0.011 | 0.97 | -0.069 | 0.082 | **5.1×10^-9^** |
| rs12903285 | 78778953 | A | 0.38 | 0.99 | -0.043 | 2.5×10^-4^ | 1.00 | -0.053 | 0.016 | 1.00 | -0.060 | 0.011 | 1.00 | -0.067 | 0.018 | 1.00 | -0.054 | 0.18 | **1.5×10^-8^** |
| rs8192477 | 78910463 | C | 0.26 | 1.00 | -0.040 | 0.0014 | 0.97 | -0.054 | 0.029 | 0.97 | -0.065 | 0.015 | 0.97 | -0.14 | 7.6×10^-6^ | 0.97 | -0.032 | 0.46 | **1.5×10^-8^** |
| rs8192478 | 78910462 | G | 0.26 | 1.00 | -0.040 | 0.0014 | 0.97 | -0.054 | 0.029 | 0.97 | -0.065 | 0.015 | 0.97 | -0.14 | 7.6×10^-6^ | 0.97 | -0.032 | 0.46 | **1.5×10^-8^** |
| rs72366880 | 78868963 | A | 0.22 | 1.00 | -0.041 | 0.0017 | 0.99 | -0.052 | 0.032 | 1.00 | -0.067 | 0.011 | 1.00 | -0.12 | 2.6×10^-4^ | 1.00 | -0.075 | 0.087 | **1.8×10^-8^** |
| rs4299116 | 78766194 | T | 0.38 | 1.00 | -0.041 | 3.5×10^-4^ | 1.00 | -0.051 | 0.021 | 1.00 | -0.062 | 0.0086 | 1.00 | -0.070 | 0.014 | 1.00 | -0.054 | 0.18 | **1.9×10^-8^** |
| rs138110943 | 78785544 | C | 0.21 | 1.00 | -0.046 | 5.6×10^-4^ | 1.00 | -0.045 | 0.075 | 1.00 | -0.085 | 0.0014 | 1.00 | -0.068 | 0.041 | 1.00 | -0.10 | 0.022 | **2.0×10^-8^** |
| rs3832992 | 78739233 | C | 0.21 | 1.00 | -0.046 | 5.0×10^-4^ | 0.99 | -0.045 | 0.081 | 1.00 | -0.085 | 0.0016 | 1.00 | -0.067 | 0.043 | 1.00 | -0.10 | 0.022 | **2.1×10^-8^** |
| rs8042238 | 78774271 | C | 0.38 | 1.00 | -0.041 | 3.9×10^-4^ | 1.00 | -0.051 | 0.021 | 1.00 | -0.063 | 0.0080 | 1.00 | -0.068 | 0.017 | 1.00 | -0.057 | 0.16 | **2.2×10^-8^** |
| rs1504549 | 78766629 | C | 0.38 | 1.00 | -0.041 | 3.9×10^-4^ | 1.00 | -0.051 | 0.021 | 1.00 | -0.062 | 0.0085 | 1.00 | -0.069 | 0.015 | 1.00 | -0.054 | 0.18 | **2.3×10^-8^** |
| rs12910910 | 78767850 | C | 0.38 | 1.00 | -0.041 | 3.9×10^-4^ | 1.00 | -0.051 | 0.021 | 1.00 | -0.062 | 0.0085 | 1.00 | -0.069 | 0.015 | 1.00 | -0.054 | 0.18 | **2.3×10^-8^** |
| rs8042260 | 78774374 | A | 0.38 | 1.00 | -0.041 | 4.2×10^-4^ | 1.00 | -0.051 | 0.021 | 1.00 | -0.063 | 0.0080 | 1.00 | -0.068 | 0.017 | 1.00 | -0.055 | 0.18 | **2.5×10^-8^** |
| rs11072766 | 78771546 | T | 0.21 | 1.00 | -0.048 | 3.5×10^-4^ | 1.00 | -0.046 | 0.084 | 1.00 | -0.086 | 0.0023 | 1.00 | -0.068 | 0.047 | 1.00 | -0.10 | 0.033 | **2.5×10^-8^** |
| rs12592111 | 78767346 | G | 0.38 | 1.00 | -0.041 | 4.6×10^-4^ | 1.00 | -0.051 | 0.021 | 1.00 | -0.062 | 0.0085 | 1.00 | -0.070 | 0.014 | 1.00 | -0.054 | 0.18 | **2.6×10^-8^** |
| rs28602670 | 78768167 | G | 0.21 | 1.00 | -0.048 | 3.7×10^-4^ | 1.00 | -0.047 | 0.081 | 1.00 | -0.086 | 0.0023 | 1.00 | -0.068 | 0.047 | 1.00 | -0.10 | 0.033 | **2.7×10^-8^** |
| rs2568485 | 78752114 | C | 0.21 | 1.00 | -0.049 | 3.4×10^-4^ | 1.00 | -0.046 | 0.088 | 1.00 | -0.084 | 0.0029 | 1.00 | -0.068 | 0.046 | 1.00 | -0.10 | 0.028 | **2.8×10^-8^** |
| rs11637656 | 78751961 | C | 0.38 | 1.00 | -0.041 | 3.9×10^-4^ | 1.00 | -0.050 | 0.023 | 1.00 | -0.061 | 0.010 | 1.00 | -0.069 | 0.015 | 1.00 | -0.054 | 0.18 | **2.9×10^-8^** |
| rs2938674 | 78757913 | A | 0.21 | 1.00 | -0.048 | 3.8×10^-4^ | 1.00 | -0.046 | 0.089 | 1.00 | -0.086 | 0.0023 | 1.00 | -0.068 | 0.044 | 1.00 | -0.099 | 0.033 | **2.9×10^-8^** |
| rs958025 | 78759348 | T | 0.21 | 1.00 | -0.048 | 4.3×10^-4^ | 1.00 | -0.046 | 0.084 | 1.00 | -0.086 | 0.0023 | 1.00 | -0.068 | 0.047 | 1.00 | -0.10 | 0.027 | **3.0×10^-8^** |
| rs28480606 | 78762313 | G | 0.21 | 1.00 | -0.048 | 4.3×10^-4^ | 1.00 | -0.046 | 0.084 | 1.00 | -0.086 | 0.0023 | 1.00 | -0.068 | 0.047 | 1.00 | -0.10 | 0.027 | **3.0×10^-8^** |
| rs7174190 | 78763617 | T | 0.21 | 1.00 | -0.048 | 4.3×10^-4^ | 1.00 | -0.046 | 0.084 | 1.00 | -0.086 | 0.0023 | 1.00 | -0.068 | 0.047 | 1.00 | -0.10 | 0.027 | **3.0×10^-8^** |
| rs2456020 | 78868398 | T | 0.23 | 1.00 | -0.042 | 0.0013 | 0.99 | -0.051 | 0.047 | 1.00 | -0.064 | 0.020 | 1.00 | -0.12 | 2.1×10^-4^ | 1.00 | -0.075 | 0.098 | **3.1×10^-8^** |
| rs2568483 | 78752343 | G | 0.21 | 1.00 | -0.048 | 3.8×10^-4^ | 1.00 | -0.046 | 0.089 | 1.00 | -0.084 | 0.0029 | 1.00 | -0.068 | 0.046 | 1.00 | -0.10 | 0.028 | **3.1×10^-8^** |
| rs8043227 | 78768871 | C | 0.38 | 1.00 | -0.040 | 5.2×10^-4^ | 1.00 | -0.051 | 0.021 | 1.00 | -0.062 | 0.0085 | 1.00 | -0.069 | 0.015 | 1.00 | -0.054 | 0.18 | **3.2×10^-8^** |
| rs4362358 | 78796104 | C | 0.38 | 1.00 | -0.040 | 6.5×10^-4^ | 0.99 | -0.052 | 0.019 | 0.99 | -0.063 | 0.0080 | 0.99 | -0.069 | 0.016 | 0.99 | -0.058 | 0.15 | **3.3×10^-8^** |
| rs2656069 | 78745707 | C | 0.21 | 1.00 | -0.048 | 3.8×10^-4^ | 1.00 | -0.046 | 0.090 | 1.00 | -0.084 | 0.0031 | 1.00 | -0.068 | 0.046 | 1.00 | -0.10 | 0.028 | **3.3×10^-8^** |
| rs2656071 | 78745343 | T | 0.21 | 1.00 | -0.048 | 3.9×10^-4^ | 1.00 | -0.046 | 0.089 | 1.00 | -0.084 | 0.0031 | 1.00 | -0.068 | 0.046 | 1.00 | -0.10 | 0.028 | **3.4×10^-8^** |
| rs36146269 | 78779510 | T | 0.38 | 1.00 | -0.040 | 5.9×10^-4^ | 1.00 | -0.051 | 0.021 | 1.00 | -0.063 | 0.0079 | 1.00 | -0.069 | 0.015 | 1.00 | -0.055 | 0.18 | **3.4×10^-8^** |
| rs12899351 | 78792398 | T | 0.38 | 1.00 | -0.040 | 6.0×10^-4^ | 1.00 | -0.051 | 0.021 | 1.00 | -0.063 | 0.0080 | 1.00 | -0.068 | 0.016 | 1.00 | -0.057 | 0.16 | **3.5×10^-8^** |
| rs2958719 | 78743029 | G | 0.21 | 1.00 | -0.048 | 4.1×10^-4^ | 1.00 | -0.046 | 0.086 | 1.00 | -0.084 | 0.0031 | 1.00 | -0.068 | 0.046 | 1.00 | -0.10 | 0.028 | **3.6×10^-8^** |
| rs2036529 | 78726272 | A | 0.21 | 1.00 | -0.048 | 4.6×10^-4^ | 1.00 | -0.043 | 0.11 | 0.99 | -0.088 | 0.0018 | 0.99 | -0.067 | 0.049 | 1.00 | -0.10 | 0.027 | **3.6×10^-8^** |
| rs12594711 | 78793921 | C | 0.38 | 1.00 | -0.040 | 6.2×10^-4^ | 1.00 | -0.051 | 0.021 | 1.00 | -0.063 | 0.0080 | 1.00 | -0.068 | 0.016 | 1.00 | -0.057 | 0.16 | **3.6×10^-8^** |
| rs77438389 | 78726271 | G | 0.21 | 1.00 | -0.048 | 4.6×10^-4^ | 1.00 | -0.043 | 0.11 | 0.99 | -0.088 | 0.0018 | 0.99 | -0.067 | 0.049 | 1.00 | -0.10 | 0.027 | **3.7×10^-8^** |
| rs4887059 | 78782095 | C | 0.38 | 1.00 | -0.040 | 6.4×10^-4^ | 1.00 | -0.051 | 0.021 | 1.00 | -0.063 | 0.0079 | 1.00 | -0.069 | 0.015 | 1.00 | -0.055 | 0.18 | **3.8×10^-8^** |
| rs12593229 | 78765290 | T | 0.38 | 1.00 | -0.040 | 6.0×10^-4^ | 1.00 | -0.051 | 0.021 | 1.00 | -0.062 | 0.0085 | 1.00 | -0.069 | 0.015 | 1.00 | -0.054 | 0.18 | **3.8×10^-8^** |
| rs2568488 | 78736593 | T | 0.21 | 1.00 | -0.048 | 4.7×10^-4^ | 1.00 | -0.046 | 0.089 | 1.00 | -0.084 | 0.0031 | 1.00 | -0.069 | 0.044 | 1.00 | -0.10 | 0.026 | **3.8×10^-8^** |
| rs2656072 | 78744292 | A | 0.21 | 1.00 | -0.048 | 4.4×10^-4^ | 1.00 | -0.046 | 0.088 | 1.00 | -0.084 | 0.0031 | 1.00 | -0.068 | 0.046 | 1.00 | -0.10 | 0.028 | **3.9×10^-8^** |
| rs2656070 | 78730252 | A | 0.21 | 1.00 | -0.047 | 4.9×10^-4^ | 1.00 | -0.044 | 0.10 | 0.99 | -0.087 | 0.0021 | 0.99 | -0.068 | 0.048 | 1.00 | -0.10 | 0.026 | **4.0×10^-8^** |
| rs9672608 | 78797463 | A | 0.21 | 1.00 | -0.045 | 7.4×10^-4^ | 0.97 | -0.047 | 0.082 | 0.98 | -0.087 | 0.0025 | 0.98 | -0.073 | 0.034 | 0.97 | -0.11 | 0.019 | **4.1×10^-8^** |
| rs12903295 | 78778972 | A | 0.38 | 1.00 | -0.040 | 5.9×10^-4^ | 1.00 | -0.053 | 0.016 | 1.00 | -0.061 | 0.010 | 1.00 | -0.066 | 0.021 | 1.00 | -0.055 | 0.18 | **4.1×10^-8^** |
| rs924840 | 78731808 | T | 0.21 | 1.00 | -0.047 | 5.2×10^-4^ | 1.00 | -0.044 | 0.10 | 0.99 | -0.087 | 0.0022 | 0.99 | -0.068 | 0.047 | 1.00 | -0.10 | 0.026 | **4.3×10^-8^** |
| rs12916801 | 78769130 | A | 0.38 | 1.00 | -0.040 | 5.2×10^-4^ | 1.00 | -0.049 | 0.025 | 1.00 | -0.061 | 0.0095 | 1.00 | -0.069 | 0.016 | 1.00 | -0.052 | 0.20 | **4.4×10^-8^** |
| rs7164594 | 78803057 | T | 0.22 | 1.00 | -0.044 | 7.2×10^-4^ | 0.97 | -0.037 | 0.16 | 0.99 | -0.068 | 0.017 | 0.98 | -0.12 | 5.5×10^-4^ | 0.99 | -0.10 | 0.030 | **4.4×10^-8^** |
| rs1964678 | 78754000 | A | 0.38 | 1.00 | -0.039 | 7.6×10^-4^ | 1.00 | -0.051 | 0.021 | 1.00 | -0.064 | 0.0072 | 1.00 | -0.069 | 0.015 | 1.00 | -0.055 | 0.18 | **4.4×10^-8^** |
| rs12904234 | 78779384 | C | 0.38 | 1.00 | -0.040 | 5.9×10^-4^ | 1.00 | -0.053 | 0.016 | 1.00 | -0.061 | 0.010 | 1.00 | -0.064 | 0.024 | 1.00 | -0.056 | 0.17 | **4.4×10^-8^** |
| rs2656073 | 78742276 | T | 0.21 | 1.00 | -0.047 | 5.1×10^-4^ | 1.00 | -0.046 | 0.086 | 1.00 | -0.084 | 0.0032 | 1.00 | -0.068 | 0.046 | 1.00 | -0.10 | 0.028 | **4.6×10^-8^** |
| rs569207 | 78873119 | T | 0.22 | 1.00 | -0.041 | 0.0018 | 0.99 | -0.053 | 0.040 | 1.00 | -0.066 | 0.017 | 1.00 | -0.12 | 3.7×10^-4^ | 1.00 | -0.076 | 0.094 | **4.6×10^-8^** |
| rs5019044 | 78796282 | A | 0.21 | 0.99 | -0.046 | 7.0×10^-4^ | 0.98 | -0.046 | 0.093 | 0.98 | -0.086 | 0.0029 | 0.99 | -0.077 | 0.024 | 0.98 | -0.10 | 0.031 | **4.7×10^-8^** |
| rs2568490 | 78738370 | T | 0.21 | 1.00 | -0.047 | 5.5×10^-4^ | 1.00 | -0.046 | 0.088 | 1.00 | -0.083 | 0.0032 | 1.00 | -0.068 | 0.045 | 1.00 | -0.10 | 0.027 | **4.9×10^-8^** |
| rs2656074 | 78741384 | T | 0.21 | 1.00 | -0.047 | 5.4×10^-4^ | 1.00 | -0.046 | 0.087 | 1.00 | -0.084 | 0.0032 | 1.00 | -0.068 | 0.046 | 1.00 | -0.10 | 0.028 | **4.9×10^-8^** |
| rs13180 | 78789488 | C | 0.38 | 1.00 | -0.039 | 8.1×10^-4^ | 1.00 | -0.050 | 0.022 | 1.00 | -0.063 | 0.0079 | 1.00 | -0.070 | 0.014 | 1.00 | -0.055 | 0.18 | 5.1×10^-8^ |
| rs667282 | 78863472 | C | 0.22 | 1.00 | -0.041 | 0.0019 | 0.99 | -0.053 | 0.040 | 1.00 | -0.066 | 0.018 | 1.00 | -0.12 | 3.3×10^-4^ | 1.00 | -0.074 | 0.10 | 5.1×10^-8^ |
| rs576982 | 78870803 | T | 0.22 | 1.00 | -0.041 | 0.0020 | 0.99 | -0.053 | 0.040 | 1.00 | -0.066 | 0.018 | 1.00 | -0.12 | 3.6×10^-4^ | 1.00 | -0.075 | 0.096 | 5.2×10^-8^ |
| rs637137 | 78873976 | A | 0.22 | 1.00 | -0.041 | 0.0020 | 0.99 | -0.053 | 0.040 | 1.00 | -0.066 | 0.017 | 1.00 | -0.12 | 3.8×10^-4^ | 1.00 | -0.076 | 0.094 | 5.2×10^-8^ |
| rs4887057 | 78760918 | A | 0.38 | 1.00 | -0.040 | 6.2×10^-4^ | 1.00 | -0.051 | 0.021 | 1.00 | -0.060 | 0.011 | 1.00 | -0.067 | 0.018 | 1.00 | -0.054 | 0.18 | 5.3×10^-8^ |
| rs2915695 | 78739471 | T | 0.21 | 1.00 | -0.047 | 6.0×10^-4^ | 1.00 | -0.046 | 0.088 | 1.00 | -0.084 | 0.0031 | 1.00 | -0.068 | 0.046 | 1.00 | -0.10 | 0.027 | 5.4×10^-8^ |
| rs2568497 | 78721397 | G | 0.21 | 0.99 | -0.047 | 5.1×10^-4^ | 0.99 | -0.042 | 0.12 | 0.99 | -0.086 | 0.0023 | 1.00 | -0.067 | 0.051 | 1.00 | -0.10 | 0.027 | 5.5×10^-8^ |
| rs2568499 | 78722359 | T | 0.21 | 1.00 | -0.047 | 5.3×10^-4^ | 0.99 | -0.042 | 0.11 | 1.00 | -0.086 | 0.0023 | 1.00 | -0.066 | 0.052 | 1.00 | -0.10 | 0.026 | 5.5×10^-8^ |
| rs74925218 | 78796732 | T | 0.21 | 0.99 | -0.045 | 8.8×10^-4^ | 0.98 | -0.047 | 0.084 | 0.98 | -0.087 | 0.0024 | 0.99 | -0.071 | 0.038 | 0.98 | -0.11 | 0.021 | 5.6×10^-8^ |
| rs11072763 | 78724256 | A | 0.21 | 1.00 | -0.046 | 7.8×10^-4^ | 0.99 | -0.036 | 0.18 | 0.99 | -0.090 | 0.0015 | 0.99 | -0.084 | 0.013 | 0.99 | -0.093 | 0.046 | 5.7×10^-8^ |
| rs2656056 | 78722519 | T | 0.21 | 1.00 | -0.047 | 5.4×10^-4^ | 0.99 | -0.041 | 0.13 | 0.99 | -0.085 | 0.0027 | 0.99 | -0.070 | 0.041 | 0.99 | -0.10 | 0.026 | 5.9×10^-8^ |
| rs2568493 | 78740233 | G | 0.21 | 1.00 | -0.047 | 6.0×10^-4^ | 1.00 | -0.046 | 0.087 | 1.00 | -0.083 | 0.0032 | 1.00 | -0.068 | 0.046 | 1.00 | -0.10 | 0.032 | 5.9×10^-8^ |
| rs28511883 | 78783683 | T | 0.21 | 1.00 | -0.045 | 7.5×10^-4^ | 1.00 | -0.046 | 0.083 | 1.00 | -0.086 | 0.0022 | 1.00 | -0.067 | 0.047 | 1.00 | -0.10 | 0.032 | 6.0×10^-8^ |
| rs7359276 | 78892661 | C | 0.23 | 1.00 | -0.042 | 0.0014 | 1.00 | -0.048 | 0.057 | 1.00 | -0.064 | 0.020 | 1.00 | -0.12 | 4.7×10^-4^ | 1.00 | -0.072 | 0.11 | 6.0×10^-8^ |
| chr15_78835915 | 78835915 | TG | 0.22 | 1.00 | -0.042 | 0.0012 | 0.97 | -0.033 | 0.19 | 0.98 | -0.074 | 0.0070 | 0.98 | -0.12 | 3.7×10^-4^ | 0.98 | -0.086 | 0.059 | 6.2×10^-8^ |
| rs2938671 | 78732754 | A | 0.21 | 1.00 | -0.047 | 5.6×10^-4^ | 0.99 | -0.046 | 0.089 | 0.99 | -0.085 | 0.0026 | 0.99 | -0.065 | 0.055 | 0.99 | -0.094 | 0.043 | 6.2×10^-8^ |
| rs11418931 | 78907997 | AT | 0.23 | 1.00 | -0.040 | 0.0020 | 1.00 | -0.047 | 0.051 | 1.00 | -0.065 | 0.014 | 1.00 | -0.11 | 6.4×10^-4^ | 1.00 | -0.073 | 0.093 | 6.5×10^-8^ |
| rs35031105 | 78772806 | T | 0.21 | 1.00 | -0.045 | 8.2×10^-4^ | 1.00 | -0.046 | 0.084 | 1.00 | -0.086 | 0.0023 | 1.00 | -0.068 | 0.047 | 1.00 | -0.10 | 0.032 | 6.8×10^-8^ |
| rs7174348 | 78792439 | A | 0.21 | 1.00 | -0.045 | 7.3×10^-4^ | 0.99 | -0.046 | 0.092 | 0.99 | -0.086 | 0.0026 | 0.99 | -0.071 | 0.038 | 0.99 | -0.097 | 0.039 | 6.9×10^-8^ |
| rs10851906 | 78774676 | G | 0.21 | 1.00 | -0.045 | 8.5×10^-4^ | 1.00 | -0.047 | 0.083 | 1.00 | -0.086 | 0.0022 | 1.00 | -0.067 | 0.047 | 1.00 | -0.10 | 0.032 | 6.9×10^-8^ |
| rs7183604 | 78899213 | T | 0.23 | 1.00 | -0.041 | 0.0019 | 1.00 | -0.048 | 0.058 | 1.00 | -0.070 | 0.012 | 1.00 | -0.12 | 5.6×10^-4^ | 1.00 | -0.072 | 0.11 | 7.1×10^-8^ |
| rs11637630 | 78899719 | G | 0.23 | 1.00 | -0.041 | 0.0019 | 1.00 | -0.048 | 0.058 | 1.00 | -0.070 | 0.013 | 1.00 | -0.11 | 5.7×10^-4^ | 1.00 | -0.072 | 0.11 | 7.2×10^-8^ |
| rs2656055 | 78720194 | C | 0.21 | 1.00 | -0.047 | 4.6×10^-4^ | 0.99 | -0.039 | 0.15 | 0.99 | -0.087 | 0.0022 | 0.99 | -0.068 | 0.047 | 1.00 | -0.094 | 0.045 | 7.3×10^-8^ |
| rs2036533 | 78781687 | A | 0.21 | 1.00 | -0.045 | 9.0×10^-4^ | 1.00 | -0.047 | 0.083 | 1.00 | -0.086 | 0.0022 | 1.00 | -0.067 | 0.047 | 1.00 | -0.10 | 0.032 | 7.4×10^-8^ |
| rs1062980 | 78792527 | C | 0.38 | 1.00 | -0.040 | 6.0×10^-4^ | 1.00 | -0.053 | 0.016 | 1.00 | -0.058 | 0.014 | 1.00 | -0.061 | 0.033 | 1.00 | -0.053 | 0.19 | 7.5×10^-8^ |
| rs16969894 | 78776456 | T | 0.21 | 1.00 | -0.044 | 9.9×10^-4^ | 1.00 | -0.047 | 0.078 | 1.00 | -0.085 | 0.0024 | 1.00 | -0.070 | 0.040 | 1.00 | -0.098 | 0.035 | 7.6×10^-8^ |
| rs12101809 | 78779801 | T | 0.21 | 1.00 | -0.045 | 9.3×10^-4^ | 1.00 | -0.046 | 0.083 | 1.00 | -0.086 | 0.0022 | 1.00 | -0.067 | 0.047 | 1.00 | -0.10 | 0.032 | 7.7×10^-8^ |
| rs9788682 | 78802586 | A | 0.21 | 1.00 | -0.044 | 0.0010 | 0.96 | -0.035 | 0.20 | 0.98 | -0.077 | 0.0086 | 0.97 | -0.11 | 0.0015 | 0.98 | -0.11 | 0.024 | 7.7×10^-8^ |
| rs664172 | 78862762 | A | 0.22 | 1.00 | -0.041 | 0.0018 | 0.97 | -0.046 | 0.088 | 0.99 | -0.073 | 0.0095 | 0.99 | -0.12 | 6.4×10^-4^ | 0.99 | -0.080 | 0.082 | 7.8×10^-8^ |
| rs3743078 | 78894759 | C | 0.23 | 1.00 | -0.042 | 0.0014 | 1.00 | -0.045 | 0.076 | 1.00 | -0.065 | 0.019 | 1.00 | -0.12 | 5.0×10^-4^ | 1.00 | -0.072 | 0.11 | 8.0×10^-8^ |
| rs12910984 | 78891627 | G | 0.23 | 1.00 | -0.041 | 0.0019 | 1.00 | -0.051 | 0.047 | 1.00 | -0.067 | 0.016 | 1.00 | -0.11 | 8.3×10^-4^ | 1.00 | -0.072 | 0.11 | 8.3×10^-8^ |
| rs17484235 | 78761414 | G | 0.37 | NA | NA | NA | 0.98 | 0.073 | 8.1×10^-4^ | 0.99 | 0.055 | 0.026 | 0.99 | 0.10 | 3.7×10^-4^ | 1.00 | 0.052 | 0.21 | 8.9×10^-8^ |
| rs965604 | 78789223 | G | 0.38 | 1.00 | -0.037 | 0.0013 | 1.00 | -0.051 | 0.021 | 1.00 | -0.062 | 0.0084 | 1.00 | -0.069 | 0.015 | 1.00 | -0.055 | 0.18 | 8.9×10^-8^ |
| rs57064725 | 78833036 | A | 0.22 | 1.00 | -0.043 | 9.3×10^-4^ | 0.99 | -0.030 | 0.24 | 1.00 | -0.070 | 0.014 | 1.00 | -0.12 | 4.0×10^-4^ | 1.00 | -0.086 | 0.062 | 1.1×10^-7^ |
| rs938682 | 78896547 | G | 0.23 | 1.00 | -0.040 | 0.0023 | 1.00 | -0.048 | 0.059 | 1.00 | -0.070 | 0.012 | 1.00 | -0.11 | 7.1×10^-4^ | 1.00 | -0.068 | 0.13 | 1.1×10^-7^ |
| rs2036534 | 78826948 | C | 0.22 | 1.00 | -0.042 | 0.0012 | 1.00 | -0.035 | 0.18 | 1.00 | -0.069 | 0.016 | 1.00 | -0.12 | 6.3×10^-4^ | 1.00 | -0.091 | 0.048 | 1.1×10^-7^ |
| rs905739 | 78845110 | G | 0.22 | 1.00 | -0.042 | 0.0015 | 0.96 | -0.033 | 0.20 | 0.99 | -0.071 | 0.013 | 0.99 | -0.12 | 2.8×10^-4^ | 0.99 | -0.084 | 0.067 | 1.2×10^-7^ |
| rs28498264 | 78837673 | T | 0.22 | 1.00 | -0.042 | 0.0013 | 0.96 | -0.030 | 0.27 | 0.98 | -0.074 | 0.010 | 0.98 | -0.12 | 4.2×10^-4^ | 0.98 | -0.098 | 0.037 | 1.2×10^-7^ |
| rs201696144 | 78961263 | A | 0.48 | 1.00 | 0.039 | 3.6×10^-4^ | 0.98 | -0.036 | 0.070 | 0.99 | -0.041 | 0.065 | 0.98 | -0.068 | 0.012 | 0.98 | -0.069 | 0.061 | 1.4×10^-7^ |
| rs8042374 | 78908032 | G | 0.23 | 1.00 | -0.040 | 0.0023 | 1.00 | -0.048 | 0.059 | 1.00 | -0.065 | 0.020 | 1.00 | -0.11 | 8.7×10^-4^ | 1.00 | -0.076 | 0.090 | 1.4×10^-7^ |
| rs8042494 | 78908010 | T | 0.23 | 1.00 | -0.040 | 0.0023 | 1.00 | -0.047 | 0.067 | 1.00 | -0.066 | 0.018 | 1.00 | -0.11 | 7.6×10^-4^ | 1.00 | -0.073 | 0.10 | 1.5×10^-7^ |
| rs12438181 | 78812098 | A | 0.22 | 1.00 | -0.041 | 0.0016 | 0.98 | -0.036 | 0.17 | 0.99 | -0.071 | 0.013 | 0.98 | -0.12 | 7.9×10^-4^ | 0.99 | -0.090 | 0.052 | 1.5×10^-7^ |
| rs7177514 | 78907406 | G | 0.23 | 1.00 | -0.040 | 0.0023 | 1.00 | -0.047 | 0.064 | 1.00 | -0.065 | 0.020 | 1.00 | -0.11 | 8.7×10^-4^ | 1.00 | -0.074 | 0.10 | 1.6×10^-7^ |
| rs12441354 | 78821016 | A | 0.22 | 1.00 | -0.041 | 0.0017 | 0.99 | -0.037 | 0.16 | 1.00 | -0.068 | 0.017 | 0.99 | -0.12 | 6.0×10^-4^ | 1.00 | -0.091 | 0.051 | 1.6×10^-7^ |
| rs28437878 | 78807872 | T | 0.22 | 1.00 | -0.041 | 0.0016 | 0.98 | -0.039 | 0.14 | 0.99 | -0.066 | 0.020 | 0.98 | -0.11 | 8.5×10^-4^ | 0.99 | -0.093 | 0.044 | 1.6×10^-7^ |
| rs7163730 | 78814681 | G | 0.22 | 1.00 | -0.041 | 0.0016 | 0.98 | -0.038 | 0.14 | 1.00 | -0.067 | 0.019 | 0.99 | -0.12 | 6.9×10^-4^ | 1.00 | -0.090 | 0.053 | 1.6×10^-7^ |
| rs8042059 | 78907859 | C | 0.23 | 1.00 | -0.040 | 0.0023 | 1.00 | -0.047 | 0.064 | 1.00 | -0.065 | 0.020 | 1.00 | -0.11 | 8.7×10^-4^ | 1.00 | -0.073 | 0.10 | 1.7×10^-7^ |
| rs7181245 | 78814389 | T | 0.22 | 1.00 | -0.042 | 0.0015 | 0.98 | -0.034 | 0.20 | 1.00 | -0.069 | 0.015 | 0.99 | -0.12 | 6.2×10^-4^ | 1.00 | -0.089 | 0.053 | 1.7×10^-7^ |
| rs8043009 | 78908154 | C | 0.23 | 1.00 | -0.040 | 0.0023 | 1.00 | -0.047 | 0.065 | 1.00 | -0.065 | 0.020 | 1.00 | -0.11 | 8.7×10^-4^ | 1.00 | -0.072 | 0.11 | 1.7×10^-7^ |
| rs59133824 | 78833450 | A | 0.22 | 1.00 | -0.042 | 0.0014 | 0.99 | -0.031 | 0.24 | 0.99 | -0.071 | 0.013 | 0.99 | -0.12 | 4.2×10^-4^ | 0.99 | -0.085 | 0.066 | 1.7×10^-7^ |
| rs6495308 | 78907656 | C | 0.23 | 1.00 | -0.040 | 0.0024 | 1.00 | -0.047 | 0.064 | 1.00 | -0.065 | 0.020 | 1.00 | -0.11 | 8.7×10^-4^ | 1.00 | -0.073 | 0.10 | 1.7×10^-7^ |
| rs59683676 | 78833453 | C | 0.22 | 1.00 | -0.042 | 0.0014 | 0.99 | -0.031 | 0.24 | 0.99 | -0.071 | 0.013 | 0.99 | -0.12 | 4.3×10^-4^ | 0.99 | -0.085 | 0.066 | 1.7×10^-7^ |
| rs12441426 | 78812329 | C | 0.22 | 1.00 | -0.041 | 0.0016 | 0.98 | -0.039 | 0.14 | 1.00 | -0.065 | 0.022 | 0.98 | -0.12 | 7.7×10^-4^ | 0.99 | -0.090 | 0.052 | 1.8×10^-7^ |
| rs35212593 | 78831826 | A | 0.22 | 1.00 | -0.042 | 0.0014 | 1.00 | -0.032 | 0.22 | 1.00 | -0.071 | 0.013 | 1.00 | -0.12 | 6.2×10^-4^ | 1.00 | -0.086 | 0.062 | 1.8×10^-7^ |
| rs61204066 | 78815298 | A | 0.22 | 1.00 | -0.041 | 0.0018 | 0.98 | -0.038 | 0.15 | 1.00 | -0.067 | 0.019 | 0.99 | -0.12 | 6.9×10^-4^ | 1.00 | -0.090 | 0.052 | 1.8×10^-7^ |
| rs3813570 | 78832832 | C | 0.22 | 1.00 | -0.042 | 0.0014 | 0.99 | -0.032 | 0.22 | 1.00 | -0.071 | 0.013 | 1.00 | -0.12 | 5.8×10^-4^ | 1.00 | -0.082 | 0.075 | 2.0×10^-7^ |
| rs7169584 | 78822660 | T | 0.22 | 1.00 | -0.040 | 0.0020 | 1.00 | -0.036 | 0.17 | 1.00 | -0.069 | 0.016 | 1.00 | -0.12 | 6.1×10^-4^ | 1.00 | -0.091 | 0.050 | 2.0×10^-7^ |
| rs7181447 | 78814567 | G | 0.22 | 1.00 | -0.041 | 0.0017 | 0.98 | -0.034 | 0.20 | 1.00 | -0.069 | 0.015 | 0.99 | -0.12 | 6.1×10^-4^ | 1.00 | -0.089 | 0.053 | 2.0×10^-7^ |
| rs7171869 | 78900909 | A | 0.24 | 1.00 | -0.041 | 0.0018 | 0.94 | -0.050 | 0.050 | 0.95 | -0.059 | 0.034 | 0.95 | -0.11 | 0.0012 | 0.95 | -0.064 | 0.16 | 2.1×10^-7^ |
| rs12438659 | 78824924 | A | 0.22 | 1.00 | -0.040 | 0.0021 | 1.00 | -0.036 | 0.17 | 1.00 | -0.069 | 0.016 | 1.00 | -0.12 | 6.0×10^-4^ | 1.00 | -0.091 | 0.049 | 2.1×10^-7^ |
| rs4461039 | 78817447 | T | 0.22 | 1.00 | -0.041 | 0.0019 | 0.99 | -0.033 | 0.21 | 1.00 | -0.070 | 0.014 | 0.99 | -0.12 | 5.8×10^-4^ | 1.00 | -0.091 | 0.050 | 2.2×10^-7^ |
| rs12439240 | 78829091 | C | 0.22 | 1.00 | -0.041 | 0.0017 | 1.00 | -0.032 | 0.22 | 1.00 | -0.071 | 0.013 | 1.00 | -0.12 | 6.0×10^-4^ | 1.00 | -0.086 | 0.062 | 2.3×10^-7^ |
| rs1700006 | 78875623 | G | 0.21 | 1.00 | -0.041 | 0.0020 | 0.95 | -0.043 | 0.12 | 0.99 | -0.071 | 0.014 | 0.97 | -0.10 | 0.0030 | 0.98 | -0.097 | 0.040 | 2.3×10^-7^ |
| rs34138960 | 78831668 | G | 0.22 | 1.00 | -0.041 | 0.0018 | 1.00 | -0.032 | 0.22 | 1.00 | -0.071 | 0.013 | 1.00 | -0.12 | 6.1×10^-4^ | 1.00 | -0.086 | 0.062 | 2.3×10^-7^ |
| rs34664138 | 78831624 | G | 0.22 | 1.00 | -0.041 | 0.0018 | 1.00 | -0.032 | 0.22 | 1.00 | -0.071 | 0.013 | 1.00 | -0.12 | 6.1×10^-4^ | 1.00 | -0.086 | 0.062 | 2.3×10^-7^ |
| rs71448806 | 78913353 | C | 0.45 | NA | NA | NA | 0.99 | 0.057 | 0.0043 | 0.99 | 0.056 | 0.012 | 0.99 | 0.093 | 7.3×10^-4^ | 0.99 | 0.057 | 0.13 | 2.3×10^-7^ |
| rs1711731 | 78861918 | A | 0.21 | 1.00 | -0.041 | 0.0023 | 0.94 | -0.044 | 0.11 | 0.98 | -0.072 | 0.013 | 0.97 | -0.11 | 0.0028 | 0.97 | -0.097 | 0.042 | 2.3×10^-7^ |
| rs7173514 | 78849918 | T | 0.23 | 1.00 | -0.040 | 0.0019 | 0.95 | -0.029 | 0.27 | 0.98 | -0.063 | 0.025 | 0.98 | -0.13 | 1.9×10^-4^ | 0.97 | -0.097 | 0.038 | 2.5×10^-7^ |
| rs503464 | 78857896 | A | 0.22 | 0.98 | -0.041 | 0.0019 | 0.96 | -0.030 | 0.25 | 0.98 | -0.062 | 0.029 | 0.98 | -0.13 | 1.3×10^-4^ | 0.97 | -0.082 | 0.074 | 2.8×10^-7^ |
| rs113352275 | 78840567 | T | 0.21 | 1.00 | -0.042 | 0.0018 | 0.94 | -0.025 | 0.36 | 0.99 | -0.076 | 0.0089 | 0.97 | -0.12 | 9.6×10^-4^ | 0.98 | -0.10 | 0.029 | 2.8×10^-7^ |
| rs201432274 | 78910461 | C | 0.25 | NA | NA | NA | 0.96 | -0.052 | 0.028 | 0.97 | -0.066 | 0.0096 | 0.97 | -0.14 | 6.6×10^-6^ | 0.97 | -0.037 | 0.40 | 2.9×10^-7^ |
| rs2869032 | 78714561 | C | 0.20 | 1.00 | -0.043 | 0.0018 | 0.99 | -0.040 | 0.14 | 1.00 | -0.085 | 0.0029 | 1.00 | -0.077 | 0.026 | 1.00 | -0.089 | 0.059 | 3.1×10^-7^ |
| rs28395178 | 78850558 | A | 0.21 | 1.00 | -0.042 | 0.0017 | 0.94 | -0.025 | 0.36 | 0.98 | -0.075 | 0.010 | 0.97 | -0.12 | 0.0012 | 0.97 | -0.10 | 0.030 | 3.2×10^-7^ |
| rs6495309 | 78915245 | T | 0.21 | 1.00 | -0.041 | 0.0023 | 1.00 | -0.045 | 0.081 | 0.99 | -0.068 | 0.017 | 0.99 | -0.11 | 0.0013 | 1.00 | -0.062 | 0.18 | 3.2×10^-7^ |
| rs13329271 | 78914230 | C | 0.21 | 1.00 | -0.040 | 0.0027 | 1.00 | -0.046 | 0.078 | 0.99 | -0.067 | 0.018 | 0.99 | -0.11 | 0.0012 | 1.00 | -0.062 | 0.18 | 3.7×10^-7^ |
| rs2869045 | 78718899 | T | 0.20 | 0.99 | -0.042 | 0.0022 | 0.99 | -0.041 | 0.13 | 1.00 | -0.085 | 0.0030 | 1.00 | -0.077 | 0.027 | 1.00 | -0.089 | 0.059 | 3.9×10^-7^ |
| rs4887077 | 78978364 | T | 0.41 | 1.00 | 0.046 | 6.0×10^-5^ | 1.00 | 0.027 | 0.20 | 0.99 | 0.048 | 0.047 | 0.99 | 0.059 | 0.035 | 1.00 | 0.032 | 0.41 | 3.9×10^-7^ |
| rs684513 | 78858400 | G | 0.21 | 1.00 | -0.041 | 0.0020 | 0.94 | -0.025 | 0.36 | 0.97 | -0.075 | 0.011 | 0.97 | -0.11 | 0.0012 | 0.97 | -0.10 | 0.032 | 4.1×10^-7^ |
| rs28681284 | 78908565 | T | 0.21 | 1.00 | -0.040 | 0.0027 | 0.95 | -0.039 | 0.16 | 0.99 | -0.074 | 0.010 | 0.97 | -0.10 | 0.0048 | 0.97 | -0.095 | 0.043 | 4.6×10^-7^ |
| rs4887053 | 78712699 | A | 0.20 | 1.00 | -0.042 | 0.0021 | 1.00 | -0.037 | 0.17 | 1.00 | -0.090 | 0.0017 | 1.00 | -0.072 | 0.039 | 1.00 | -0.089 | 0.062 | 4.7×10^-7^ |
| rs200422183 | 78719501 | AT | 0.38 | NA | NA | NA | 0.98 | 0.068 | 8.9×10^-4^ | 0.99 | 0.049 | 0.038 | 0.98 | 0.085 | 0.0027 | 0.98 | 0.056 | 0.16 | 4.9×10^-7^ |
| rs72743199 | 78975855 | T | 0.40 | 1.00 | 0.046 | 7.2×10^-5^ | 1.00 | 0.026 | 0.21 | 0.99 | 0.048 | 0.048 | 0.99 | 0.060 | 0.033 | 0.99 | 0.032 | 0.42 | 5.0×10^-7^ |
| rs3825845 | 78910258 | T | 0.22 | 1.00 | -0.037 | 0.0052 | 0.99 | -0.046 | 0.072 | 1.00 | -0.067 | 0.017 | 0.99 | -0.11 | 7.4×10^-4^ | 1.00 | -0.065 | 0.16 | 5.3×10^-7^ |
| rs12901913 | 78981348 | T | 0.41 | 1.00 | 0.043 | 1.6×10^-4^ | 1.00 | 0.029 | 0.17 | 0.99 | 0.049 | 0.043 | 0.99 | 0.059 | 0.037 | 1.00 | 0.041 | 0.30 | 6.3×10^-7^ |
| rs146474671 | 78774982 | A | 0.09 | 0.99 | 0.083 | 1.8×10^-5^ | 0.81 | 0.042 | 0.26 | 0.83 | 0.10 | 0.025 | 0.83 | 0.008 | 0.88 | 0.82 | 0.15 | 0.055 | 7.1×10^-7^ |
| rs1996371 | 78956806 | C | 0.41 | 1.00 | 0.044 | 1.4×10^-4^ | 1.00 | 0.026 | 0.22 | 1.00 | 0.049 | 0.040 | 1.00 | 0.059 | 0.034 | 1.00 | 0.038 | 0.33 | 7.5×10^-7^ |
| rs11853054 | 79054919 | A | 0.37 | 1.00 | 0.040 | 3.8×10^-4^ | 0.77 | 0.043 | 0.093 | 0.81 | 0.066 | 0.019 | 0.81 | 0.053 | 0.11 | 0.81 | 0.064 | 0.17 | 7.8×10^-7^ |
| rs35238224 | 78945980 | A | 0.41 | 1.00 | 0.041 | 2.5×10^-4^ | 0.98 | 0.024 | 0.23 | 0.98 | 0.048 | 0.036 | 0.98 | 0.060 | 0.030 | 0.99 | 0.049 | 0.21 | 8.6×10^-7^ |
| rs56136424 | 79023805 | CAAAGT | 0.40 | 1.00 | 0.043 | 1.5×10^-4^ | 0.92 | 0.026 | 0.23 | 0.94 | 0.054 | 0.022 | 0.91 | 0.065 | 0.027 | 0.92 | 0.015 | 0.70 | 8.7×10^-7^ |
| rs11072790 | 78992025 | T | 0.42 | 1.00 | 0.045 | 8.5×10^-5^ | 0.98 | 0.025 | 0.24 | 0.98 | 0.048 | 0.047 | 0.99 | 0.056 | 0.046 | 0.99 | 0.029 | 0.47 | 8.9×10^-7^ |
| rs922692 | 78984214 | A | 0.41 | 1.00 | 0.043 | 1.6×10^-4^ | 1.00 | 0.028 | 0.19 | 0.99 | 0.048 | 0.045 | 0.99 | 0.060 | 0.035 | 1.00 | 0.032 | 0.41 | 8.9×10^-7^ |
| rs11638372 | 78983559 | T | 0.41 | 1.00 | 0.043 | 1.6×10^-4^ | 1.00 | 0.028 | 0.19 | 0.99 | 0.048 | 0.046 | 0.99 | 0.060 | 0.035 | 1.00 | 0.032 | 0.41 | 9.0×10^-7^ |
| rs35186448 | 78888234 | ACCCC | 0.22 | 1.00 | -1.44 | 0.45 | 1.00 | -0.050 | 0.037 | 1.00 | -0.070 | 0.0078 | 1.00 | -0.11 | 4.4×10^-4^ | 1.00 | -0.072 | 0.097 | 9.2×10^-7^ |
| rs11853608 | 79030693 | C | 0.41 | 1.00 | 0.045 | 9.7×10^-5^ | 0.96 | 0.025 | 0.24 | 0.96 | 0.046 | 0.059 | 0.94 | 0.059 | 0.042 | 0.94 | 0.037 | 0.36 | 9.2×10^-7^ |
| rs11638830 | 78948319 | C | 0.41 | 1.00 | 0.043 | 1.6×10^-4^ | 1.00 | 0.024 | 0.25 | 1.00 | 0.049 | 0.039 | 1.00 | 0.058 | 0.040 | 1.00 | 0.043 | 0.27 | 9.3×10^-7^ |
| rs71148554 | 78966167 | GT | 0.41 | 1.00 | 0.042 | 2.0×10^-4^ | 1.00 | 0.024 | 0.24 | 1.00 | 0.050 | 0.028 | 1.00 | 0.060 | 0.030 | 1.00 | 0.033 | 0.38 | 9.3×10^-7^ |
| rs11072801 | 79054932 | C | 0.37 | 0.99 | 0.040 | 3.8×10^-4^ | 0.77 | 0.042 | 0.096 | 0.81 | 0.064 | 0.022 | 0.81 | 0.051 | 0.12 | 0.81 | 0.064 | 0.17 | 9.3×10^-7^ |
| rs4886584 | 79021464 | A | 0.40 | 1.00 | 0.043 | 2.1×10^-4^ | 0.94 | 0.030 | 0.18 | 0.95 | 0.048 | 0.050 | 0.92 | 0.067 | 0.024 | 0.93 | 0.033 | 0.42 | 9.3×10^-7^ |
| rs4887069 | 78909070 | G | 0.23 | 1.00 | -0.038 | 0.0032 | 0.98 | -0.040 | 0.11 | 0.98 | -0.060 | 0.031 | 0.98 | -0.11 | 0.0015 | 0.98 | -0.065 | 0.15 | 9.4×10^-7^ |
| rs4887084 | 79025593 | C | 0.41 | 1.00 | 0.043 | 1.7×10^-4^ | 0.99 | 0.026 | 0.22 | 1.00 | 0.052 | 0.031 | 0.97 | 0.058 | 0.041 | 0.97 | 0.031 | 0.43 | 9.5×10^-7^ |
| rs11072791 | 78997076 | A | 0.41 | 1.00 | 0.043 | 1.6×10^-4^ | 0.99 | 0.024 | 0.25 | 0.99 | 0.052 | 0.032 | 0.99 | 0.061 | 0.031 | 0.99 | 0.029 | 0.46 | 1.0×10^-6^ |
| rs2170311 | 78947611 | A | 0.41 | 1.00 | 0.043 | 1.9×10^-4^ | 0.99 | 0.025 | 0.24 | 0.99 | 0.049 | 0.044 | 0.99 | 0.059 | 0.037 | 0.99 | 0.045 | 0.25 | 1.0×10^-6^ |
| rs4886583 | 79021445 | A | 0.40 | 1.00 | 0.042 | 2.4×10^-4^ | 0.93 | 0.031 | 0.16 | 0.94 | 0.047 | 0.058 | 0.91 | 0.067 | 0.024 | 0.92 | 0.034 | 0.41 | 1.1×10^-6^ |
| rs28590060 | 79052312 | A | 0.40 | 0.98 | 0.046 | 8.0×10^-5^ | 0.92 | 0.017 | 0.44 | 0.94 | 0.052 | 0.032 | 0.94 | 0.062 | 0.033 | 0.94 | 0.031 | 0.45 | 1.1×10^-6^ |
| rs4886582 | 79021441 | G | 0.40 | 1.00 | 0.042 | 2.5×10^-4^ | 0.94 | 0.030 | 0.18 | 0.95 | 0.048 | 0.050 | 0.92 | 0.067 | 0.024 | 0.93 | 0.033 | 0.42 | 1.1×10^-6^ |
| rs2869566 | 79026496 | T | 0.41 | 1.00 | 0.043 | 1.6×10^-4^ | 0.99 | 0.025 | 0.23 | 1.00 | 0.050 | 0.037 | 0.97 | 0.057 | 0.044 | 0.97 | 0.033 | 0.40 | 1.1×10^-6^ |
| rs4887087 | 79026541 | T | 0.41 | 1.00 | 0.043 | 1.6×10^-4^ | 0.99 | 0.025 | 0.23 | 1.00 | 0.050 | 0.037 | 0.97 | 0.057 | 0.044 | 0.97 | 0.033 | 0.40 | 1.1×10^-6^ |
| rs6495314 | 78960529 | C | 0.41 | 1.00 | 0.042 | 2.4×10^-4^ | 1.00 | 0.029 | 0.16 | 1.00 | 0.050 | 0.038 | 1.00 | 0.054 | 0.054 | 1.00 | 0.034 | 0.39 | 1.2×10^-6^ |
| rs3894347 | 79026001 | A | 0.41 | 1.00 | 0.043 | 2.1×10^-4^ | 0.98 | 0.029 | 0.18 | 0.99 | 0.049 | 0.042 | 0.97 | 0.054 | 0.058 | 0.97 | 0.038 | 0.34 | 1.2×10^-6^ |
| rs12899135 | 78954379 | G | 0.41 | 1.00 | 0.042 | 2.4×10^-4^ | 1.00 | 0.025 | 0.24 | 1.00 | 0.049 | 0.040 | 1.00 | 0.059 | 0.036 | 1.00 | 0.041 | 0.29 | 1.3×10^-6^ |
| rs1825084 | 79023577 | T | 0.40 | 1.00 | 0.042 | 2.5×10^-4^ | 0.93 | 0.025 | 0.26 | 0.94 | 0.055 | 0.028 | 0.91 | 0.072 | 0.015 | 0.92 | 0.020 | 0.63 | 1.3×10^-6^ |
| rs1825085 | 79023578 | G | 0.40 | 1.00 | 0.042 | 2.5×10^-4^ | 0.93 | 0.025 | 0.26 | 0.94 | 0.055 | 0.028 | 0.91 | 0.072 | 0.015 | 0.92 | 0.020 | 0.63 | 1.3×10^-6^ |
| rs11852980 | 79054911 | G | 0.37 | 0.99 | 0.041 | 3.3×10^-4^ | 0.78 | 0.041 | 0.11 | 0.82 | 0.061 | 0.029 | 0.81 | 0.049 | 0.14 | 0.81 | 0.054 | 0.25 | 1.4×10^-6^ |
| rs189218934 | 78903987 | T | 0.26 | 0.99 | -0.040 | 0.0020 | 0.86 | -0.045 | 0.070 | 0.86 | -0.052 | 0.053 | 0.86 | -0.085 | 0.0093 | 0.86 | -0.056 | 0.22 | 1.5×10^-6^ |
| rs4886585 | 79026315 | A | 0.39 | 1.00 | 0.043 | 1.7×10^-4^ | 0.88 | 0.021 | 0.36 | 0.90 | 0.064 | 0.014 | 0.88 | 0.069 | 0.025 | 0.88 | 0.008 | 0.85 | 1.5×10^-6^ |
| rs80162468 | 79026316 | C | 0.39 | 1.00 | 0.043 | 1.8×10^-4^ | 0.88 | 0.021 | 0.36 | 0.90 | 0.064 | 0.014 | 0.88 | 0.069 | 0.025 | 0.88 | 0.008 | 0.85 | 1.5×10^-6^ |
| rs11634351 | 78944718 | A | 0.41 | 1.00 | 0.042 | 3.0×10^-4^ | 0.98 | 0.023 | 0.28 | 0.98 | 0.049 | 0.042 | 0.98 | 0.060 | 0.034 | 0.99 | 0.049 | 0.22 | 1.6×10^-6^ |
| rs12910627 | 78994933 | C | 0.41 | 1.00 | 0.043 | 1.8×10^-4^ | 0.98 | 0.024 | 0.25 | 0.98 | 0.049 | 0.041 | 0.99 | 0.057 | 0.042 | 0.99 | 0.030 | 0.45 | 1.6×10^-6^ |
| rs3971860 | 79026278 | G | 0.34 | 0.96 | 0.043 | 2.3×10^-4^ | 0.78 | 0.039 | 0.14 | 0.81 | 0.066 | 0.022 | 0.79 | 0.080 | 0.019 | 0.80 | -0.024 | 0.61 | 1.7×10^-6^ |
| rs12902602 | 78967401 | G | 0.41 | 1.00 | 0.042 | 2.4×10^-4^ | 1.00 | 0.024 | 0.26 | 1.00 | 0.050 | 0.038 | 1.00 | 0.060 | 0.034 | 1.00 | 0.033 | 0.40 | 1.7×10^-6^ |
| rs4886586 | 79026674 | A | 0.41 | 1.00 | 0.042 | 2.2×10^-4^ | 0.95 | 0.027 | 0.21 | 0.95 | 0.052 | 0.033 | 0.94 | 0.051 | 0.080 | 0.93 | 0.036 | 0.37 | 1.7×10^-6^ |
| rs11633178 | 78944538 | G | 0.41 | 1.00 | 0.041 | 3.0×10^-4^ | 0.98 | 0.023 | 0.28 | 0.98 | 0.048 | 0.047 | 0.98 | 0.060 | 0.034 | 0.99 | 0.049 | 0.22 | 1.7×10^-6^ |
| rs1021071 | 78968179 | C | 0.41 | 1.00 | 0.042 | 2.5×10^-4^ | 1.00 | 0.024 | 0.26 | 1.00 | 0.050 | 0.038 | 1.00 | 0.060 | 0.034 | 1.00 | 0.033 | 0.40 | 1.7×10^-6^ |
| rs4887083 | 79025577 | T | 0.42 | 1.00 | 0.043 | 1.7×10^-4^ | 0.99 | 0.027 | 0.20 | 1.00 | 0.047 | 0.051 | 0.97 | 0.050 | 0.078 | 0.97 | 0.034 | 0.39 | 1.8×10^-6^ |
| rs8038543 | 79017685 | T | 0.41 | 1.00 | 0.042 | 2.1×10^-4^ | 0.98 | 0.022 | 0.30 | 0.98 | 0.047 | 0.050 | 0.96 | 0.062 | 0.031 | 0.96 | 0.037 | 0.35 | 1.8×10^-6^ |
| rs7170068 | 78912943 | A | 0.21 | 0.99 | -0.036 | 0.0084 | 0.99 | -0.048 | 0.065 | 0.98 | -0.065 | 0.025 | 0.98 | -0.11 | 0.0012 | 0.99 | -0.058 | 0.22 | 1.8×10^-6^ |
| rs200246180 | 79018854 | TA | 0.33 | 0.84 | 0.043 | 1.8×10^-4^ | 0.99 | 0.025 | 0.21 | 1.00 | 0.046 | 0.044 | 0.97 | 0.050 | 0.072 | 0.97 | 0.032 | 0.41 | 1.8×10^-6^ |
| rs11072785 | 78968229 | T | 0.41 | 1.00 | 0.042 | 2.5×10^-4^ | 1.00 | 0.024 | 0.26 | 1.00 | 0.049 | 0.043 | 1.00 | 0.060 | 0.034 | 1.00 | 0.033 | 0.41 | 1.9×10^-6^ |
| rs12908207 | 79025963 | T | 0.42 | 0.99 | 0.043 | 1.8×10^-4^ | 0.99 | 0.027 | 0.20 | 1.00 | 0.046 | 0.052 | 0.97 | 0.050 | 0.078 | 0.97 | 0.034 | 0.39 | 1.9×10^-6^ |
| rs4886579 | 78969256 | T | 0.41 | 1.00 | 0.042 | 2.4×10^-4^ | 0.99 | 0.024 | 0.26 | 1.00 | 0.048 | 0.047 | 1.00 | 0.060 | 0.033 | 1.00 | 0.032 | 0.41 | 1.9×10^-6^ |
| rs12593950 | 78920935 | C | 0.22 | 1.00 | -0.035 | 0.0082 | 0.97 | -0.042 | 0.11 | 0.97 | -0.076 | 0.0076 | 0.97 | -0.10 | 0.0028 | 0.97 | -0.055 | 0.24 | 2.0×10^-6^ |
| rs11639372 | 78966655 | T | 0.41 | 1.00 | 0.042 | 2.6×10^-4^ | 0.99 | 0.023 | 0.28 | 1.00 | 0.049 | 0.039 | 1.00 | 0.059 | 0.035 | 1.00 | 0.033 | 0.40 | 2.0×10^-6^ |
| rs11634543 | 79005504 | T | 0.41 | 1.00 | 0.043 | 2.0×10^-4^ | 0.99 | 0.024 | 0.26 | 0.99 | 0.048 | 0.047 | 1.00 | 0.056 | 0.047 | 1.00 | 0.030 | 0.44 | 2.0×10^-6^ |
| rs62010337 | 78924542 | T | 0.46 | 0.86 | -0.040 | 8.9×10^-4^ | 0.77 | -0.017 | 0.50 | 0.79 | -0.065 | 0.013 | 0.78 | -0.090 | 0.0041 | 0.78 | -0.038 | 0.39 | 2.1×10^-6^ |
| rs61496709 | 79022432 | G | 0.40 | 0.97 | 0.041 | 4.3×10^-4^ | 0.98 | 0.025 | 0.22 | 0.99 | 0.049 | 0.032 | 0.96 | 0.057 | 0.040 | 0.95 | 0.034 | 0.39 | 2.2×10^-6^ |
| rs28669908 | 78910267 | A | 0.20 | 1.00 | -0.037 | 0.0068 | 0.95 | -0.037 | 0.18 | 0.99 | -0.075 | 0.0098 | 0.97 | -0.10 | 0.0066 | 0.97 | -0.089 | 0.062 | 2.2×10^-6^ |
| rs4886580 | 78969385 | G | 0.41 | 1.00 | 0.042 | 2.4×10^-4^ | 0.99 | 0.025 | 0.24 | 1.00 | 0.048 | 0.047 | 1.00 | 0.055 | 0.048 | 1.00 | 0.032 | 0.41 | 2.2×10^-6^ |
| rs4887086 | 79026529 | T | 0.42 | 1.00 | 0.042 | 2.2×10^-4^ | 0.99 | 0.028 | 0.19 | 1.00 | 0.046 | 0.052 | 0.97 | 0.050 | 0.080 | 0.97 | 0.034 | 0.38 | 2.2×10^-6^ |
| rs2869561 | 79025516 | C | 0.42 | 1.00 | 0.042 | 2.2×10^-4^ | 0.99 | 0.027 | 0.20 | 1.00 | 0.047 | 0.051 | 0.97 | 0.050 | 0.078 | 0.97 | 0.034 | 0.39 | 2.2×10^-6^ |
| rs28534575 | 78923845 | G | 0.22 | 1.00 | -0.035 | 0.0085 | 0.97 | -0.041 | 0.12 | 0.97 | -0.078 | 0.0063 | 0.97 | -0.10 | 0.0037 | 0.96 | -0.054 | 0.24 | 2.3×10^-6^ |
| rs3971859 | 79026277 | T | 0.33 | 0.95 | 0.043 | 3.4×10^-4^ | 0.78 | 0.039 | 0.14 | 0.81 | 0.066 | 0.022 | 0.79 | 0.080 | 0.019 | 0.80 | -0.024 | 0.61 | 2.4×10^-6^ |
| rs4886587 | 79026822 | A | 0.42 | 1.00 | 0.042 | 2.2×10^-4^ | 0.99 | 0.027 | 0.20 | 1.00 | 0.045 | 0.058 | 0.97 | 0.050 | 0.079 | 0.97 | 0.034 | 0.39 | 2.5×10^-6^ |
| rs56354501 | 79033520 | G | 0.42 | 1.00 | 0.040 | 4.1×10^-4^ | 0.97 | 0.030 | 0.16 | 0.98 | 0.047 | 0.049 | 0.96 | 0.059 | 0.039 | 0.96 | 0.026 | 0.52 | 2.5×10^-6^ |
| rs4887072 | 78925435 | G | 0.22 | 1.00 | -0.035 | 0.0082 | 0.96 | -0.039 | 0.13 | 0.97 | -0.078 | 0.0062 | 0.97 | -0.10 | 0.0042 | 0.97 | -0.053 | 0.25 | 2.8×10^-6^ |
| rs8031513 | 79034276 | C | 0.42 | 1.00 | 0.040 | 4.4×10^-4^ | 0.97 | 0.029 | 0.17 | 0.98 | 0.048 | 0.047 | 0.96 | 0.059 | 0.038 | 0.96 | 0.026 | 0.52 | 2.8×10^-6^ |
| rs11638490 | 79007950 | T | 0.42 | 1.00 | 0.041 | 3.2×10^-4^ | 0.99 | 0.025 | 0.24 | 0.99 | 0.048 | 0.044 | 1.00 | 0.055 | 0.050 | 1.00 | 0.030 | 0.44 | 2.9×10^-6^ |
| rs11629637 | 79019024 | T | 0.42 | 1.00 | 0.042 | 2.4×10^-4^ | 1.00 | 0.025 | 0.24 | 1.00 | 0.047 | 0.047 | 0.97 | 0.050 | 0.079 | 0.97 | 0.034 | 0.39 | 2.9×10^-6^ |
| rs7182642 | 79052277 | T | 0.41 | 0.99 | 0.042 | 2.4×10^-4^ | 0.94 | 0.021 | 0.34 | 0.96 | 0.047 | 0.052 | 0.96 | 0.064 | 0.026 | 0.95 | 0.027 | 0.50 | 3.0×10^-6^ |
| rs11639375 | 79024214 | A | 0.42 | 1.00 | 0.042 | 2.3×10^-4^ | 0.99 | 0.025 | 0.24 | 1.00 | 0.047 | 0.051 | 0.97 | 0.049 | 0.081 | 0.97 | 0.034 | 0.39 | 3.0×10^-6^ |
| rs11639382 | 79024268 | A | 0.42 | 1.00 | 0.042 | 2.3×10^-4^ | 0.99 | 0.025 | 0.24 | 1.00 | 0.047 | 0.051 | 0.97 | 0.049 | 0.081 | 0.97 | 0.034 | 0.39 | 3.0×10^-6^ |
| rs11639049 | 79023730 | G | 0.42 | 1.00 | 0.042 | 2.4×10^-4^ | 0.99 | 0.025 | 0.24 | 1.00 | 0.047 | 0.050 | 0.97 | 0.050 | 0.081 | 0.97 | 0.034 | 0.39 | 3.1×10^-6^ |
| rs11639347 | 79024350 | T | 0.42 | 1.00 | 0.042 | 2.4×10^-4^ | 0.99 | 0.025 | 0.24 | 1.00 | 0.047 | 0.051 | 0.97 | 0.049 | 0.081 | 0.97 | 0.034 | 0.39 | 3.1×10^-6^ |
| rs11639181 | 79024016 | G | 0.42 | 1.00 | 0.042 | 2.5×10^-4^ | 0.99 | 0.025 | 0.24 | 1.00 | 0.047 | 0.051 | 0.97 | 0.049 | 0.081 | 0.97 | 0.034 | 0.39 | 3.2×10^-6^ |
| rs11639166 | 79023766 | C | 0.42 | 1.00 | 0.042 | 2.5×10^-4^ | 0.99 | 0.025 | 0.24 | 1.00 | 0.047 | 0.050 | 0.97 | 0.050 | 0.081 | 0.97 | 0.034 | 0.39 | 3.2×10^-6^ |
| rs34563625 | 79020278 | C | 0.42 | 1.00 | 0.042 | 2.5×10^-4^ | 1.00 | 0.025 | 0.24 | 1.00 | 0.047 | 0.051 | 0.97 | 0.050 | 0.080 | 0.97 | 0.034 | 0.38 | 3.2×10^-6^ |
| rs35583595 | 79020152 | G | 0.42 | 1.00 | 0.042 | 2.5×10^-4^ | 1.00 | 0.025 | 0.24 | 1.00 | 0.047 | 0.051 | 0.97 | 0.050 | 0.080 | 0.97 | 0.034 | 0.38 | 3.2×10^-6^ |
| rs1825083 | 79023467 | C | 0.42 | 1.00 | 0.042 | 2.5×10^-4^ | 1.00 | 0.025 | 0.24 | 1.00 | 0.047 | 0.050 | 0.97 | 0.050 | 0.080 | 0.97 | 0.034 | 0.39 | 3.2×10^-6^ |
| rs2869554 | 79021037 | C | 0.42 | 1.00 | 0.042 | 2.5×10^-4^ | 1.00 | 0.025 | 0.24 | 1.00 | 0.047 | 0.051 | 0.97 | 0.050 | 0.080 | 0.97 | 0.034 | 0.38 | 3.2×10^-6^ |
| rs1825082 | 79023433 | A | 0.42 | 1.00 | 0.042 | 2.5×10^-4^ | 1.00 | 0.025 | 0.24 | 1.00 | 0.047 | 0.050 | 0.97 | 0.050 | 0.080 | 0.97 | 0.034 | 0.39 | 3.2×10^-6^ |
| rs8027972 | 79022615 | C | 0.41 | 1.00 | 0.041 | 3.1×10^-4^ | 0.96 | 0.025 | 0.24 | 0.97 | 0.045 | 0.065 | 0.95 | 0.062 | 0.031 | 0.95 | 0.026 | 0.52 | 3.3×10^-6^ |
| rs34225855 | 79022136 | G | 0.41 | 0.99 | 0.041 | 3.9×10^-4^ | 0.99 | 0.026 | 0.22 | 0.99 | 0.048 | 0.046 | 0.96 | 0.054 | 0.056 | 0.97 | 0.034 | 0.39 | 3.3×10^-6^ |
| rs80229697 | 79022616 | A | 0.41 | 1.00 | 0.041 | 3.2×10^-4^ | 0.96 | 0.025 | 0.24 | 0.97 | 0.045 | 0.065 | 0.95 | 0.062 | 0.031 | 0.95 | 0.026 | 0.52 | 3.4×10^-6^ |
| rs76919723 | 79017281 | C | 0.41 | 1.00 | 0.042 | 2.6×10^-4^ | 0.99 | 0.022 | 0.30 | 0.99 | 0.047 | 0.049 | 0.97 | 0.054 | 0.057 | 0.97 | 0.035 | 0.38 | 3.4×10^-6^ |
| rs112236733 | 78783832 | GTATGTATTTATT | 0.38 | 0.99 | 0.37 | 0.66 | 0.99 | -0.049 | 0.018 | 0.99 | -0.063 | 0.0050 | 0.99 | -0.068 | 0.014 | 0.99 | -0.062 | 0.12 | 3.5×10^-6^ |
| rs11072792 | 78999911 | G | 0.42 | 1.00 | 0.042 | 2.6×10^-4^ | 0.98 | 0.017 | 0.42 | 0.99 | 0.053 | 0.027 | 0.99 | 0.056 | 0.047 | 1.00 | 0.029 | 0.46 | 3.6×10^-6^ |
| rs3829786 | 79019546 | A | 0.42 | 1.00 | 0.042 | 2.8×10^-4^ | 1.00 | 0.025 | 0.24 | 1.00 | 0.047 | 0.051 | 0.97 | 0.050 | 0.079 | 0.97 | 0.034 | 0.38 | 3.6×10^-6^ |
| rs1807007 | 79062102 | G | 0.41 | 1.00 | 0.040 | 3.9×10^-4^ | 0.87 | 0.022 | 0.33 | 0.89 | 0.049 | 0.054 | 0.89 | 0.063 | 0.034 | 0.89 | 0.043 | 0.30 | 3.7×10^-6^ |
| rs113134286 | 79012888 | T | 0.41 | 1.00 | 0.041 | 3.2×10^-4^ | 0.99 | 0.023 | 0.27 | 0.99 | 0.047 | 0.048 | 0.99 | 0.055 | 0.052 | 0.99 | 0.031 | 0.43 | 3.7×10^-6^ |
| rs3813565 | 79019610 | T | 0.41 | 1.00 | 0.040 | 5.6×10^-4^ | 1.00 | 0.023 | 0.27 | 1.00 | 0.054 | 0.024 | 0.97 | 0.052 | 0.065 | 0.97 | 0.035 | 0.37 | 4.0×10^-6^ |
| rs11630236 | 79058298 | C | 0.42 | 0.98 | 0.041 | 3.0×10^-4^ | 0.95 | 0.022 | 0.31 | 0.97 | 0.047 | 0.049 | 0.96 | 0.058 | 0.042 | 0.97 | 0.024 | 0.55 | 4.3×10^-6^ |
| rs4887090 | 79043402 | A | 0.42 | 1.00 | 0.041 | 3.2×10^-4^ | 0.96 | 0.022 | 0.31 | 0.98 | 0.048 | 0.046 | 0.97 | 0.057 | 0.043 | 0.97 | 0.024 | 0.54 | 4.5×10^-6^ |
| rs56195905 | 79040317 | C | 0.42 | 1.00 | 0.040 | 3.9×10^-4^ | 0.96 | 0.022 | 0.31 | 0.98 | 0.049 | 0.041 | 0.97 | 0.059 | 0.037 | 0.97 | 0.022 | 0.57 | 4.7×10^-6^ |
| rs4887101 | 79067449 | C | 0.29 | 1.00 | 0.038 | 8.3×10^-4^ | 0.64 | 0.059 | 0.10 | 0.68 | 0.087 | 0.022 | 0.66 | 0.070 | 0.13 | 0.66 | 0.089 | 0.17 | 5.3×10^-6^ |
| rs4887100 | 79067448 | C | 0.29 | 1.00 | 0.038 | 8.3×10^-4^ | 0.64 | 0.059 | 0.10 | 0.68 | 0.087 | 0.022 | 0.66 | 0.070 | 0.13 | 0.66 | 0.089 | 0.17 | 5.3×10^-6^ |
| rs200227748 | 78835913 | TTTG | 0.21 | NA | NA | NA | 0.97 | -0.033 | 0.19 | 0.98 | -0.074 | 0.0070 | 0.98 | -0.12 | 3.7×10^-4^ | 0.98 | -0.086 | 0.059 | 5.3×10^-6^ |
| rs201677370 | 78835914 | TTG | 0.21 | NA | NA | NA | 0.97 | -0.033 | 0.19 | 0.98 | -0.074 | 0.0070 | 0.98 | -0.12 | 3.7×10^-4^ | 0.98 | -0.086 | 0.059 | 5.3×10^-6^ |
| rs55834964 | 79049766 | A | 0.42 | 1.00 | 0.040 | 4.5×10^-4^ | 0.95 | 0.022 | 0.30 | 0.98 | 0.049 | 0.043 | 0.97 | 0.058 | 0.042 | 0.97 | 0.023 | 0.56 | 5.6×10^-6^ |
| rs141502139 | 78747916 | AAAAAAG | 0.37 | NA | NA | NA | 1.00 | -0.051 | 0.015 | 1.00 | -0.060 | 0.0075 | 1.00 | -0.069 | 0.013 | 1.00 | -0.050 | 0.20 | 5.6×10^-6^ |
| rs12906653 | 79052580 | A | 0.42 | 0.99 | 0.042 | 2.7×10^-4^ | 0.95 | 0.022 | 0.31 | 0.98 | 0.043 | 0.073 | 0.97 | 0.057 | 0.046 | 0.97 | 0.023 | 0.56 | 5.9×10^-6^ |
| rs899984 | 79042736 | C | 0.42 | 1.00 | 0.040 | 4.8×10^-4^ | 0.96 | 0.022 | 0.31 | 0.98 | 0.048 | 0.045 | 0.97 | 0.057 | 0.043 | 0.97 | 0.024 | 0.54 | 6.5×10^-6^ |
| rs11633351 | 79056815 | T | 0.43 | 1.00 | 0.040 | 4.0×10^-4^ | 0.95 | 0.022 | 0.31 | 0.97 | 0.045 | 0.063 | 0.96 | 0.056 | 0.049 | 0.97 | 0.026 | 0.52 | 7.1×10^-6^ |
| rs8043119 | 79061002 | A | 0.43 | 1.00 | 0.040 | 3.9×10^-4^ | 0.94 | 0.022 | 0.31 | 0.97 | 0.045 | 0.064 | 0.96 | 0.056 | 0.050 | 0.96 | 0.025 | 0.52 | 7.1×10^-6^ |
| rs12286 | 79051759 | A | 0.42 | 0.99 | 0.039 | 7.4×10^-4^ | 0.94 | 0.024 | 0.27 | 0.97 | 0.048 | 0.048 | 0.96 | 0.059 | 0.038 | 0.96 | 0.028 | 0.48 | 7.4×10^-6^ |
| rs1809419 | 79053814 | A | 0.42 | 1.00 | 0.040 | 3.9×10^-4^ | 0.95 | 0.021 | 0.33 | 0.97 | 0.045 | 0.062 | 0.97 | 0.057 | 0.047 | 0.97 | 0.024 | 0.55 | 7.5×10^-6^ |
| rs11635931 | 79064080 | A | 0.43 | 1.00 | 0.040 | 4.0×10^-4^ | 0.94 | 0.022 | 0.30 | 0.97 | 0.044 | 0.066 | 0.96 | 0.055 | 0.052 | 0.96 | 0.025 | 0.53 | 7.5×10^-6^ |
| rs1809409 | 79063474 | T | 0.43 | 1.00 | 0.040 | 3.9×10^-4^ | 0.94 | 0.023 | 0.29 | 0.96 | 0.045 | 0.063 | 0.96 | 0.055 | 0.056 | 0.96 | 0.021 | 0.59 | 7.7×10^-6^ |
| rs12916648 | 79054129 | T | 0.42 | 0.99 | 0.040 | 4.0×10^-4^ | 0.95 | 0.023 | 0.29 | 0.98 | 0.043 | 0.073 | 0.97 | 0.057 | 0.046 | 0.97 | 0.023 | 0.56 | 7.7×10^-6^ |
| rs4887095 | 79053207 | A | 0.42 | 1.00 | 0.040 | 3.9×10^-4^ | 0.95 | 0.022 | 0.31 | 0.97 | 0.045 | 0.064 | 0.97 | 0.056 | 0.048 | 0.97 | 0.023 | 0.57 | 7.7×10^-6^ |
| rs12898346 | 79054117 | C | 0.42 | 1.00 | 0.039 | 5.2×10^-4^ | 0.95 | 0.022 | 0.31 | 0.97 | 0.049 | 0.044 | 0.96 | 0.055 | 0.052 | 0.97 | 0.022 | 0.57 | 8.0×10^-6^ |
| rs1807006 | 79062340 | G | 0.43 | 1.00 | 0.040 | 4.4×10^-4^ | 0.94 | 0.022 | 0.30 | 0.97 | 0.045 | 0.065 | 0.96 | 0.056 | 0.051 | 0.96 | 0.025 | 0.52 | 8.1×10^-6^ |
| rs200692991 | 79018174 | A | 0.36 | 0.99 | 0.040 | 4.7×10^-4^ | 0.82 | 0.014 | 0.54 | 0.85 | 0.053 | 0.040 | 0.83 | 0.082 | 0.0094 | 0.84 | 0.009 | 0.84 | 8.1×10^-6^ |
| rs11635870 | 79064143 | G | 0.43 | 1.00 | 0.040 | 4.3×10^-4^ | 0.94 | 0.022 | 0.30 | 0.97 | 0.044 | 0.066 | 0.96 | 0.055 | 0.052 | 0.96 | 0.025 | 0.53 | 8.1×10^-6^ |
| rs4420500 | 78962964 | T | 0.43 | 1.00 | 0.039 | 6.4×10^-4^ | 0.99 | 0.017 | 0.43 | 0.99 | 0.057 | 0.017 | 0.99 | 0.047 | 0.090 | 1.00 | 0.034 | 0.38 | 8.4×10^-6^ |
| rs12898323 | 79054084 | C | 0.42 | 1.00 | 0.039 | 5.4×10^-4^ | 0.95 | 0.024 | 0.27 | 0.97 | 0.044 | 0.070 | 0.97 | 0.057 | 0.045 | 0.97 | 0.024 | 0.54 | 8.5×10^-6^ |
| rs4887096 | 79053284 | C | 0.43 | 1.00 | 0.040 | 4.4×10^-4^ | 0.95 | 0.022 | 0.31 | 0.98 | 0.043 | 0.073 | 0.97 | 0.057 | 0.046 | 0.97 | 0.023 | 0.56 | 8.9×10^-6^ |
| rs1809420 | 79056769 | C | 0.43 | 1.00 | 0.039 | 5.1×10^-4^ | 0.95 | 0.022 | 0.31 | 0.97 | 0.045 | 0.063 | 0.96 | 0.056 | 0.049 | 0.97 | 0.026 | 0.52 | 8.9×10^-6^ |
| rs6495267 | 79057093 | A | 0.42 | 1.00 | 0.040 | 3.9×10^-4^ | 0.92 | 0.019 | 0.38 | 0.95 | 0.047 | 0.054 | 0.95 | 0.059 | 0.040 | 0.94 | 0.016 | 0.69 | 9.0×10^-6^ |
| rs144134168 | 78712101 | T | 0.33 | NA | NA | NA | 0.92 | 0.060 | 0.0056 | 0.94 | 0.045 | 0.071 | 0.93 | 0.10 | 0.0013 | 0.94 | 0.035 | 0.41 | 9.2×10^-6^ |
| rs12916326 | 79054011 | G | 0.43 | 1.00 | 0.039 | 6.1×10^-4^ | 0.95 | 0.024 | 0.27 | 0.98 | 0.044 | 0.068 | 0.97 | 0.056 | 0.049 | 0.97 | 0.023 | 0.56 | 1.0×10^-5^ |
| rs3894352 | 79059526 | G | 0.43 | 0.99 | 0.041 | 2.9×10^-4^ | 0.94 | 0.021 | 0.34 | 0.96 | 0.040 | 0.098 | 0.95 | 0.052 | 0.065 | 0.96 | 0.025 | 0.54 | 1.0×10^-5^ |
| rs2904223 | 79059691 | A | 0.43 | 1.00 | 0.041 | 3.4×10^-4^ | 0.94 | 0.020 | 0.35 | 0.97 | 0.040 | 0.098 | 0.96 | 0.055 | 0.053 | 0.96 | 0.025 | 0.52 | 1.0×10^-5^ |
| rs3894351 | 79059523 | A | 0.43 | 1.00 | 0.041 | 3.5×10^-4^ | 0.94 | 0.020 | 0.35 | 0.97 | 0.040 | 0.098 | 0.96 | 0.054 | 0.055 | 0.96 | 0.026 | 0.51 | 1.1×10^-5^ |
| rs3743063 | 79065171 | A | 0.43 | 1.00 | 0.039 | 5.1×10^-4^ | 0.93 | 0.026 | 0.24 | 0.96 | 0.043 | 0.079 | 0.95 | 0.048 | 0.089 | 0.96 | 0.023 | 0.57 | 1.2×10^-5^ |
| rs4887102 | 79067495 | G | 0.35 | 0.99 | 0.036 | 0.0016 | 0.76 | 0.025 | 0.35 | 0.80 | 0.066 | 0.020 | 0.78 | 0.071 | 0.038 | 0.79 | 0.037 | 0.43 | 1.3×10^-5^ |
| rs4887099 | 79059547 | C | 0.43 | 1.00 | 0.040 | 4.4×10^-4^ | 0.94 | 0.020 | 0.35 | 0.97 | 0.040 | 0.098 | 0.96 | 0.054 | 0.055 | 0.96 | 0.026 | 0.51 | 1.4×10^-5^ |
| rs1809423 | 79059670 | C | 0.43 | 1.00 | 0.040 | 4.6×10^-4^ | 0.94 | 0.020 | 0.35 | 0.97 | 0.040 | 0.098 | 0.96 | 0.055 | 0.054 | 0.96 | 0.025 | 0.52 | 1.4×10^-5^ |
| rs7168391 | 79055163 | T | 0.43 | 1.00 | 0.040 | 4.9×10^-4^ | 0.95 | 0.025 | 0.25 | 0.97 | 0.042 | 0.081 | 0.96 | 0.047 | 0.10 | 0.97 | 0.023 | 0.56 | 1.4×10^-5^ |
| rs4887098 | 79057950 | C | 0.42 | 1.00 | 0.041 | 2.9×10^-4^ | 0.92 | 0.018 | 0.40 | 0.95 | 0.042 | 0.084 | 0.94 | 0.050 | 0.081 | 0.95 | 0.019 | 0.64 | 1.4×10^-5^ |
| rs4887097 | 79057949 | C | 0.42 | 0.99 | 0.041 | 2.9×10^-4^ | 0.92 | 0.018 | 0.40 | 0.95 | 0.042 | 0.084 | 0.94 | 0.050 | 0.081 | 0.95 | 0.019 | 0.64 | 1.5×10^-5^ |
| rs35934157 | 79067385 | A | 0.41 | 1.00 | 0.038 | 9.7×10^-4^ | 0.93 | 0.025 | 0.26 | 0.96 | 0.048 | 0.046 | 0.95 | 0.051 | 0.076 | 0.96 | 0.023 | 0.57 | 1.5×10^-5^ |
| rs1810165 | 79059449 | G | 0.43 | 1.00 | 0.039 | 5.5×10^-4^ | 0.94 | 0.021 | 0.34 | 0.96 | 0.039 | 0.10 | 0.96 | 0.055 | 0.051 | 0.96 | 0.027 | 0.50 | 1.5×10^-5^ |
| rs12909179 | 79047632 | A | 0.39 | 0.90 | 0.041 | 9.9×10^-4^ | 0.95 | 0.023 | 0.30 | 0.97 | 0.046 | 0.058 | 0.97 | 0.059 | 0.037 | 0.96 | 0.019 | 0.62 | 1.5×10^-5^ |
| rs201710748 | 78712114 | T | 0.31 | NA | NA | NA | 0.94 | 0.058 | 0.0077 | 0.96 | 0.039 | 0.11 | 0.95 | 0.10 | 6.1×10^-4^ | 0.96 | 0.031 | 0.46 | 1.6×10^-5^ |
| rs7174367 | 79064667 | G | 0.42 | 0.98 | 0.040 | 5.6×10^-4^ | 0.91 | 0.012 | 0.59 | 0.95 | 0.050 | 0.044 | 0.93 | 0.059 | 0.041 | 0.94 | 0.026 | 0.53 | 1.6×10^-5^ |
| rs12440014 | 78926726 | G | 0.23 | 1.00 | -0.030 | 0.023 | 0.96 | -0.035 | 0.18 | 0.97 | -0.067 | 0.019 | 0.97 | -0.11 | 0.0014 | 0.96 | -0.054 | 0.24 | 1.7×10^-5^ |
| rs7161774 | 79069734 | G | 0.41 | 0.99 | 0.037 | 0.0011 | 0.92 | 0.028 | 0.21 | 0.95 | 0.046 | 0.061 | 0.94 | 0.049 | 0.092 | 0.95 | 0.021 | 0.60 | 1.9×10^-5^ |
| rs11636753 | 78928946 | T | 0.39 | 1.00 | -0.038 | 7.0×10^-4^ | 1.00 | -0.056 | 0.012 | 0.99 | -0.021 | 0.40 | 1.00 | -0.026 | 0.34 | 1.00 | -0.021 | 0.60 | 2.0×10^-5^ |
| rs28544432 | 78924538 | G | 0.19 | 0.96 | -0.034 | 0.015 | 0.89 | -0.053 | 0.075 | 0.91 | -0.047 | 0.13 | 0.91 | -0.11 | 0.0024 | 0.91 | -0.071 | 0.18 | 2.0×10^-5^ |
| rs12442456 | 78751962 | T | 0.13 | 1.00 | -0.041 | 0.011 | 0.88 | -0.061 | 0.078 | 0.95 | -0.094 | 0.0054 | 0.93 | -0.060 | 0.16 | 0.93 | -0.048 | 0.41 | 2.5×10^-5^ |
| rs7171916 | 79067951 | G | 0.41 | 1.00 | 0.038 | 9.5×10^-4^ | 0.92 | 0.026 | 0.24 | 0.95 | 0.044 | 0.069 | 0.94 | 0.043 | 0.13 | 0.95 | 0.015 | 0.71 | 3.2×10^-5^ |
| rs2277545 | 79083591 | C | 0.43 | 0.99 | 0.040 | 4.6×10^-4^ | 0.97 | 0.008 | 0.72 | 0.99 | 0.047 | 0.044 | 0.99 | 0.045 | 0.11 | 1.00 | 0.028 | 0.48 | 3.3×10^-5^ |
| rs7171578 | 79067922 | T | 0.41 | 1.00 | 0.038 | 9.1×10^-4^ | 0.93 | 0.023 | 0.28 | 0.95 | 0.043 | 0.075 | 0.94 | 0.043 | 0.14 | 0.95 | 0.018 | 0.66 | 3.7×10^-5^ |
| rs11631955 | 79085915 | G | 0.42 | 1.00 | 0.037 | 0.0011 | 0.89 | 0.006 | 0.81 | 0.92 | 0.050 | 0.043 | 0.92 | 0.054 | 0.066 | 0.93 | 0.045 | 0.28 | 4.2×10^-5^ |
| rs17487514 | 78953785 | T | 0.31 | 0.99 | 0.044 | 3.4×10^-4^ | 1.00 | 0.017 | 0.44 | 1.00 | 0.042 | 0.099 | 1.00 | 0.045 | 0.13 | 1.00 | 0.010 | 0.82 | 4.3×10^-5^ |
| rs11639044 | 79083814 | T | 0.44 | 1.00 | 0.038 | 6.9×10^-4^ | 0.97 | 0.008 | 0.69 | 0.99 | 0.047 | 0.044 | 0.98 | 0.045 | 0.11 | 1.00 | 0.025 | 0.53 | 4.5×10^-5^ |
| rs28699256 | 79058951 | C | 0.45 | 0.97 | 0.033 | 0.0033 | 0.91 | 0.028 | 0.20 | 0.94 | 0.036 | 0.14 | 0.94 | 0.062 | 0.030 | 0.95 | 0.029 | 0.47 | 4.6×10^-5^ |
| rs11854507 | 79069121 | G | 0.41 | 0.99 | 0.038 | 9.6×10^-4^ | 0.90 | 0.027 | 0.23 | 0.93 | 0.040 | 0.10 | 0.92 | 0.043 | 0.14 | 0.93 | 0.010 | 0.81 | 4.7×10^-5^ |

MAF, minor allele frequency; NA, not available.

^1^MAF was weighted by sample size across the five samples.

**Supplementary Table 3.** Chromosome 8q11 SNP and indel associations with nicotine dependence at meta-analysis *P*<5×10^-5^. Results were taken from a linear regression model in each sample with categorical nicotine dependence (mild, moderate, and severe) as the outcome, SNP/indel genotype dosage as the predictor, and age, sex, principal component eigenvectors (if applicable), and other sample-specific variables (if applicable) as covariates. The sample-specific results following correction for genomic control are shown. SNPs/indels are sorted by the meta-analysis *P* values.

| **SNP \ indel** | **Base pair position (NCBI build 37)** | **Minor allele** | **MAF^1^** | **deCODE (N=9,090)** | | | **EAGLE (N=3,006)** | | | **COPDGene (N=2,211)** | | | **COGEND (N=1,935)** | | | **SAGE (N=832)** | | | **Meta-analysis *P*** |
| --- | --- | --- | --- | --- | --- | --- | --- | --- | --- | --- | --- | --- | --- | --- | --- | --- | --- | --- | --- |
|  |  |  |  | **info** | **β** | ***P*** | **info** | **β** | ***P*** | **info** | **β** | ***P*** | **info** | **β** | ***P*** | **info** | **β** | ***P*** |  |
| rs55828312 | 42589602 | G | 0.22 | 0.99 | -0.034 | 0.0092 | 0.96 | -0.077 | 0.0028 | 0.98 | -0.0092 | 0.75 | 0.97 | -0.10 | 0.0029 | 0.98 | -0.18 | 5.0×10^-4^ | 1.2×10^-6^ |
| rs1530848 | 42552908 | G | 0.23 | 1.00 | -0.034 | 0.0083 | 0.99 | -0.072 | 0.0042 | 1.00 | -0.015 | 0.59 | 1.00 | -0.096 | 0.0037 | 1.00 | -0.15 | 0.0028 | 1.6×10^-6^ |
| rs35116962 | 42545662 | GT | 0.22 | 1.00 | -0.030 | 0.021 | 1.00 | -0.072 | 0.0026 | 1.00 | -0.012 | 0.66 | 1.00 | -0.098 | 0.0023 | 1.00 | -0.16 | 6.7×10^-4^ | 2.0×10^-6^ |
| rs34708855 | 42541592 | AT | 0.22 | 1.00 | -0.030 | 0.023 | 1.00 | -0.071 | 0.0029 | 1.00 | -0.015 | 0.59 | 1.00 | -0.093 | 0.0041 | 1.00 | -0.16 | 6.7×10^-4^ | 2.6×10^-6^ |
| rs4236926 | 42578059 | T | 0.22 | 1.00 | -0.032 | 0.013 | 0.98 | -0.071 | 0.0050 | 0.98 | -0.0093 | 0.74 | 0.98 | -0.10 | 0.0029 | 0.99 | -0.17 | 8.2×10^-4^ | 2.9×10^-6^ |
| rs16891561 | 42579739 | T | 0.22 | 1.00 | -0.033 | 0.013 | 0.97 | -0.071 | 0.0051 | 0.98 | -0.0072 | 0.80 | 0.98 | -0.10 | 0.0025 | 0.98 | -0.17 | 7.6×10^-4^ | 3.0×10^-6^ |
| rs58379124 | 42579203 | T | 0.22 | 1.00 | -0.032 | 0.014 | 0.97 | -0.072 | 0.0048 | 0.98 | -0.0089 | 0.75 | 0.98 | -0.10 | 0.0030 | 0.99 | -0.17 | 7.8×10^-4^ | 3.1×10^-6^ |
| rs7459838 | 42584279 | G | 0.22 | 1.00 | -0.032 | 0.013 | 0.97 | -0.071 | 0.0052 | 0.98 | -0.0081 | 0.77 | 0.98 | -0.10 | 0.0031 | 0.98 | -0.17 | 6.9×10^-4^ | 3.2×10^-6^ |
| rs9693825 | 42545177 | C | 0.22 | 1.00 | -0.031 | 0.018 | 1.00 | -0.072 | 0.0039 | 1.00 | -0.012 | 0.67 | 1.00 | -0.099 | 0.0033 | 1.00 | -0.16 | 0.0013 | 3.8×10^-6^ |
| rs57645595 | 42579025 | T | 0.22 | 1.00 | -0.032 | 0.016 | 0.97 | -0.071 | 0.0050 | 0.98 | -0.0088 | 0.75 | 0.98 | -0.10 | 0.0030 | 0.99 | -0.17 | 8.0×10^-4^ | 3.8×10^-6^ |
| rs7004381 | 42551161 | A | 0.22 | 1.00 | -0.030 | 0.021 | 1.00 | -0.072 | 0.0042 | 1.00 | -0.015 | 0.60 | 1.00 | -0.099 | 0.0032 | 1.00 | -0.16 | 0.0011 | 4.0×10^-6^ |
| rs4951 | 42563557 | C | 0.22 | 1.00 | -0.030 | 0.021 | 0.99 | -0.071 | 0.0044 | 0.99 | -0.015 | 0.58 | 0.99 | -0.098 | 0.0034 | 1.00 | -0.16 | 0.0013 | 4.2×10^-6^ |
| rs6474412 | 42550498 | C | 0.22 | 1.00 | -0.030 | 0.020 | 1.00 | -0.072 | 0.0042 | 1.00 | -0.012 | 0.66 | 1.00 | -0.099 | 0.0032 | 1.00 | -0.16 | 0.0013 | 4.3×10^-6^ |
| rs6474413 | 42551064 | C | 0.22 | 1.00 | -0.030 | 0.021 | 1.00 | -0.072 | 0.0039 | 1.00 | -0.013 | 0.65 | 1.00 | -0.099 | 0.0031 | 1.00 | -0.16 | 0.0011 | 4.3×10^-6^ |
| rs1955186 | 42549491 | G | 0.22 | 1.00 | -0.030 | 0.021 | 1.00 | -0.072 | 0.0042 | 1.00 | -0.013 | 0.64 | 1.00 | -0.099 | 0.0032 | 1.00 | -0.16 | 0.0013 | 4.6×10^-6^ |
| rs1955185 | 42549647 | C | 0.22 | 1.00 | -0.030 | 0.021 | 1.00 | -0.072 | 0.0042 | 1.00 | -0.013 | 0.64 | 1.00 | -0.099 | 0.0032 | 1.00 | -0.16 | 0.0013 | 4.7×10^-6^ |
| rs13277524 | 42550057 | G | 0.22 | 1.00 | -0.030 | 0.021 | 1.00 | -0.072 | 0.0042 | 1.00 | -0.013 | 0.65 | 1.00 | -0.099 | 0.0032 | 1.00 | -0.16 | 0.0013 | 4.7×10^-6^ |
| rs9643853 | 42556652 | A | 0.22 | 1.00 | -0.030 | 0.020 | 1.00 | -0.071 | 0.0047 | 1.00 | -0.014 | 0.62 | 1.00 | -0.096 | 0.0041 | 1.00 | -0.16 | 0.0013 | 4.7×10^-6^ |
| rs13273442 | 42544017 | A | 0.22 | 1.00 | -0.030 | 0.021 | 1.00 | -0.072 | 0.0042 | 1.00 | -0.013 | 0.63 | 1.00 | -0.097 | 0.0038 | 1.00 | -0.16 | 0.0013 | 4.8×10^-6^ |
| rs6985052 | 42551319 | C | 0.22 | 1.00 | -0.030 | 0.021 | 1.00 | -0.072 | 0.0043 | 1.00 | -0.013 | 0.65 | 1.00 | -0.099 | 0.0032 | 1.00 | -0.16 | 0.0013 | 4.8×10^-6^ |
| rs13254578 | 42545846 | G | 0.22 | 1.00 | -0.029 | 0.024 | 1.00 | -0.072 | 0.0041 | 1.00 | -0.014 | 0.61 | 1.00 | -0.099 | 0.0034 | 1.00 | -0.16 | 0.0013 | 5.0×10^-6^ |
| rs9792277 | 42545827 | G | 0.22 | 1.00 | -0.030 | 0.021 | 1.00 | -0.072 | 0.0041 | 1.00 | -0.012 | 0.67 | 1.00 | -0.098 | 0.0034 | 1.00 | -0.16 | 0.0013 | 5.0×10^-6^ |
| rs9792257 | 42545551 | C | 0.22 | 1.00 | -0.030 | 0.021 | 1.00 | -0.072 | 0.0042 | 1.00 | -0.012 | 0.67 | 1.00 | -0.098 | 0.0035 | 1.00 | -0.16 | 0.0013 | 5.0×10^-6^ |
| rs1901281 | 42546888 | G | 0.22 | 1.00 | -0.030 | 0.022 | 1.00 | -0.072 | 0.0041 | 1.00 | -0.013 | 0.65 | 1.00 | -0.099 | 0.0034 | 1.00 | -0.16 | 0.0013 | 5.0×10^-6^ |
| rs9693858 | 42545357 | C | 0.22 | 1.00 | -0.030 | 0.021 | 1.00 | -0.072 | 0.0042 | 1.00 | -0.012 | 0.67 | 1.00 | -0.098 | 0.0035 | 1.00 | -0.16 | 0.0013 | 5.0×10^-6^ |
| rs4736835 | 42547033 | T | 0.22 | 1.00 | -0.030 | 0.022 | 1.00 | -0.072 | 0.0041 | 1.00 | -0.012 | 0.66 | 1.00 | -0.099 | 0.0033 | 1.00 | -0.16 | 0.0013 | 5.1×10^-6^ |
| rs4736838 | 42547333 | T | 0.22 | 1.00 | -0.030 | 0.021 | 0.99 | -0.073 | 0.0037 | 1.00 | -0.0093 | 0.74 | 0.99 | -0.099 | 0.0031 | 1.00 | -0.16 | 0.0013 | 5.1×10^-6^ |
| rs4950 | 42552633 | G | 0.22 | 1.00 | -0.030 | 0.021 | 1.00 | -0.071 | 0.0045 | 1.00 | -0.014 | 0.62 | 1.00 | -0.095 | 0.0045 | 1.00 | -0.16 | 0.0013 | 5.2×10^-6^ |
| rs1451239 | 42546542 | G | 0.22 | 1.00 | -0.030 | 0.023 | 1.00 | -0.072 | 0.0041 | 1.00 | -0.012 | 0.66 | 1.00 | -0.099 | 0.0034 | 1.00 | -0.16 | 0.0013 | 5.3×10^-6^ |
| rs13280604 | 42559586 | G | 0.22 | 1.00 | -0.030 | 0.021 | 1.00 | -0.071 | 0.0047 | 1.00 | -0.013 | 0.63 | 1.00 | -0.096 | 0.0041 | 1.00 | -0.16 | 0.0013 | 5.3×10^-6^ |
| rs9643891 | 42556597 | C | 0.22 | 1.00 | -0.030 | 0.022 | 1.00 | -0.071 | 0.0047 | 1.00 | -0.014 | 0.62 | 1.00 | -0.096 | 0.0041 | 1.00 | -0.16 | 0.0013 | 5.4×10^-6^ |
| rs6474415 | 42562938 | G | 0.22 | 1.00 | -0.029 | 0.027 | 0.99 | -0.071 | 0.0044 | 0.99 | -0.016 | 0.58 | 0.99 | -0.098 | 0.0034 | 1.00 | -0.16 | 0.0011 | 5.4×10^-6^ |
| rs10958727 | 42554763 | C | 0.22 | 1.00 | -0.030 | 0.021 | 1.00 | -0.070 | 0.0050 | 1.00 | -0.014 | 0.60 | 1.00 | -0.095 | 0.0048 | 1.00 | -0.16 | 0.0012 | 5.4×10^-6^ |
| rs6474414 | 42560336 | A | 0.22 | 1.00 | -0.030 | 0.021 | 1.00 | -0.071 | 0.0048 | 1.00 | -0.014 | 0.62 | 1.00 | -0.096 | 0.0041 | 1.00 | -0.16 | 0.0013 | 5.4×10^-6^ |
| rs6997909 | 42560249 | A | 0.22 | 1.00 | -0.030 | 0.021 | 1.00 | -0.070 | 0.0049 | 1.00 | -0.014 | 0.62 | 1.00 | -0.096 | 0.0041 | 1.00 | -0.16 | 0.0013 | 5.5×10^-6^ |
| rs4295650 | 42537811 | G | 0.22 | 1.00 | -0.030 | 0.021 | 1.00 | -0.070 | 0.0053 | 1.00 | -0.015 | 0.60 | 1.00 | -0.095 | 0.0049 | 1.00 | -0.17 | 9.7×10^-4^ | 5.5×10^-6^ |
| rs13277254 | 42549982 | G | 0.22 | 1.00 | -0.030 | 0.021 | 1.00 | -0.071 | 0.0045 | 1.00 | -0.015 | 0.60 | 1.00 | -0.096 | 0.0044 | 1.00 | -0.16 | 0.0018 | 5.5×10^-6^ |
| rs6987704 | 42547623 | T | 0.20 | 1.00 | -0.032 | 0.018 | 1.00 | -0.074 | 0.0043 | 1.00 | -0.00035 | 0.99 | 1.00 | -0.11 | 0.0013 | 1.00 | -0.16 | 0.0017 | 5.5×10^-6^ |
| rs10958726 | 42535909 | G | 0.22 | 1.00 | -0.030 | 0.022 | 1.00 | -0.070 | 0.0054 | 1.00 | -0.015 | 0.60 | 1.00 | -0.095 | 0.0050 | 1.00 | -0.17 | 8.7×10^-4^ | 5.6×10^-6^ |
| rs7842601 | 42537055 | C | 0.22 | 1.00 | -0.030 | 0.022 | 1.00 | -0.070 | 0.0053 | 1.00 | -0.014 | 0.61 | 1.00 | -0.095 | 0.0050 | 1.00 | -0.17 | 9.3×10^-4^ | 5.6×10^-6^ |
| rs4736837 | 42547183 | C | 0.23 | 1.00 | -0.030 | 0.019 | 0.94 | -0.078 | 0.0018 | 0.94 | -0.0084 | 0.77 | 0.93 | -0.094 | 0.0054 | 0.95 | -0.15 | 0.0037 | 5.6×10^-6^ |
| rs11783507 | 42534395 | C | 0.22 | 1.00 | -0.030 | 0.022 | 0.99 | -0.070 | 0.0054 | 1.00 | -0.015 | 0.59 | 0.99 | -0.094 | 0.0053 | 1.00 | -0.17 | 9.6×10^-4^ | 5.8×10^-6^ |
| rs6474411 | 42541446 | A | 0.22 | 1.00 | -0.030 | 0.021 | 1.00 | -0.070 | 0.0050 | 1.00 | -0.014 | 0.62 | 1.00 | -0.095 | 0.0048 | 1.00 | -0.16 | 0.0012 | 5.8×10^-6^ |
| rs4736836 | 42547176 | C | 0.23 | 1.00 | -0.030 | 0.020 | 0.94 | -0.078 | 0.0018 | 0.94 | -0.0084 | 0.77 | 0.93 | -0.094 | 0.0054 | 0.95 | -0.15 | 0.0037 | 5.9×10^-6^ |
| rs7816726 | 42535437 | A | 0.22 | 1.00 | -0.030 | 0.021 | 1.00 | -0.070 | 0.0054 | 1.00 | -0.012 | 0.66 | 1.00 | -0.095 | 0.0051 | 1.00 | -0.17 | 8.7×10^-4^ | 6.3×10^-6^ |
| rs36011715 | 42530981 | C | 0.22 | 1.00 | -0.50 | 0.66 | 1.00 | -0.069 | 0.0038 | 1.00 | -0.013 | 0.63 | 1.00 | -0.094 | 0.0036 | 1.00 | -0.17 | 4.4×10^-4^ | 6.4×10^-6^ |
| rs78258002 | 42542494 | T | 0.22 | 1.00 | -0.030 | 0.021 | 1.00 | -0.070 | 0.0052 | 1.00 | -0.013 | 0.64 | 1.00 | -0.094 | 0.0053 | 0.99 | -0.16 | 0.0014 | 6.5×10^-6^ |
| rs77232073 | 42542356 | G | 0.22 | 1.00 | -0.030 | 0.021 | 1.00 | -0.070 | 0.0050 | 1.00 | -0.012 | 0.67 | 1.00 | -0.095 | 0.0048 | 1.00 | -0.16 | 0.0013 | 6.7×10^-6^ |
| rs1451240 | 42546711 | A | 0.22 | 1.00 | -0.029 | 0.027 | 1.00 | -0.072 | 0.0041 | 1.00 | -0.012 | 0.66 | 1.00 | -0.099 | 0.0034 | 1.00 | -0.16 | 0.0013 | 6.8×10^-6^ |
| rs1979140 | 42530836 | T | 0.22 | 1.00 | -0.030 | 0.023 | 1.00 | -0.069 | 0.0058 | 1.00 | -0.013 | 0.64 | 1.00 | -0.094 | 0.0053 | 1.00 | -0.17 | 8.7×10^-4^ | 6.9×10^-6^ |
| rs35599391 | 42540066 | C | 0.22 | 1.00 | -0.030 | 0.022 | 1.00 | -0.070 | 0.0051 | 1.00 | -0.011 | 0.69 | 0.99 | -0.095 | 0.0049 | 1.00 | -0.16 | 0.0012 | 7.3×10^-6^ |
| rs200716987 | 42603348 | T | 0.24 | 0.99 | -0.030 | 0.018 | 0.91 | -0.040 | 0.099 | 0.95 | -0.011 | 0.68 | 0.94 | -0.091 | 0.0044 | 0.94 | -0.22 | 2.4×10^-6^ | 8.1×10^-6^ |
| rs7017612 | 42599245 | C | 0.18 | 0.99 | -0.038 | 0.0053 | 0.93 | -0.060 | 0.048 | 0.96 | -0.014 | 0.66 | 0.95 | -0.10 | 0.0067 | 0.96 | -0.16 | 0.0041 | 1.0×10^-5^ |
| rs10958725 | 42524584 | T | 0.22 | 1.00 | -0.029 | 0.024 | 1.00 | -0.067 | 0.0077 | 1.00 | -0.012 | 0.66 | 1.00 | -0.088 | 0.0092 | 1.00 | -0.16 | 0.0011 | 1.3×10^-5^ |
| rs34456987 | 42523329 | G | 0.22 | 1.00 | -0.029 | 0.028 | 1.00 | -0.069 | 0.0060 | 1.00 | -0.011 | 0.68 | 1.00 | -0.088 | 0.0091 | 1.00 | -0.17 | 8.9×10^-4^ | 1.4×10^-5^ |
| rs138569594 | 42538848 | GGGAA | 0.22 | NA | NA | NA | 0.99 | -0.065 | 0.0058 | 0.99 | -0.011 | 0.68 | 0.99 | -0.086 | 0.0075 | 0.99 | -0.17 | 4.3×10^-4^ | 1.8×10^-5^ |
| rs9692914 | 42545296 | T | 0.23 | 1.00 | -0.030 | 0.022 | 0.94 | -0.069 | 0.0056 | 0.96 | 0.0040 | 0.88 | 0.95 | -0.095 | 0.0043 | 0.96 | -0.15 | 0.0029 | 2.1×10^-5^ |
| rs10958724 | 42517127 | G | 0.18 | 1.00 | -0.026 | 0.058 | 0.95 | -0.079 | 0.0055 | 0.97 | -0.015 | 0.65 | 0.97 | -0.12 | 0.0022 | 0.97 | -0.16 | 0.0058 | 3.9×10^-5^ |
| rs201404634 | 42603349 | T | 0.25 | NA | NA | NA | 0.91 | -0.040 | 0.098 | 0.95 | -0.011 | 0.68 | 0.94 | -0.091 | 0.0044 | 0.94 | -0.22 | 2.4×10^-6^ | 4.6×10^-5^ |
| chr8: 42603350 | 42603350 | T | 0.25 | NA | NA | NA | 0.91 | -0.040 | 0.099 | 0.95 | -0.011 | 0.68 | 0.94 | -0.091 | 0.0044 | 0.94 | -0.22 | 2.4×10^-6^ | 4.6×10^-5^ |

MAF, minor allele frequency; NA, not available.

^1^MAF was weighted by sample size across the five samples.

**Supplementary Table 4**. Interaction results between top nicotine dependence-associated chromosome 20q13 SNPs and indels (GWAS meta-analysis *P*<5×10^-5^) and known nicotine dependence-associated SNPs, rs16969968[^1-4^](#_ENREF_1) on chromosome 15q25 and rs6474413[^5^](#_ENREF_5)^,^ [^6^](#_ENREF_6) on chromosome 8p11. Linear regression models were run in each discovery sample with categorical nicotine dependence (mild, moderate, and severe) as the outcome, and *CHRNA4* SNP/indel genotype dosage, known SNP dosage (rs16969968-A or rs6474413-T as the minor coded allele), an interaction term between the *CHRNA4* SNP/indel and known SNP, age, sex, principal component eigenvectors (if applicable), and other sample-specific variables (if applicable) as the independent variables. The sample-specific regression coefficients for the interaction term were combined in an inverse variance-weighted meta-analysis. SNPs/indels are sorted by their chromosomal position.

| **SNP / indel** | **Base pair position**  **(NCBI build 37)** | **Minor allele** | **Meta-analysis for an interaction with rs16969968** | | **Meta-analysis for an interaction with rs6474413** | |
| --- | --- | --- | --- | --- | --- | --- |
|  |  |  | **β** | ***P*** | **β** | ***P*** |
| rs45508092 | 61974731 | A | 0.0037 | 0.89 | 0.030 | 0.33 |
| rs45456294 | 61974832 | A | -0.0016 | 0.95 | 0.023 | 0.43 |
| rs45612034 | 61974970 | A | 0.0022 | 0.93 | 0.020 | 0.50 |
| rs199666656 | 61975634 | G | -0.0010 | 0.97 | 0.024 | 0.42 |
| rs4809292 | 61977506 | A | -0.0041 | 0.88 | 0.024 | 0.42 |
| rs4809293 | 61977640 | A | 0.0063 | 0.84 | 0.018 | 0.61 |
| rs4809294 | 61977723 | T | -0.015 | 0.60 | 0.023 | 0.48 |
| rs45470098 | 61979328 | G | 0.0045 | 0.90 | 0.068 | 0.11 |
| rs45618935 | 61979347 | C | -0.0046 | 0.86 | 0.023 | 0.45 |
| rs6062899 | 61979793 | G | -0.0095 | 0.54 | 0.028 | 0.12 |
| rs6062901 | 61980261 | G | -0.0053 | 0.74 | 0.029 | 0.11 |
| rs45461993 | 61983901 | G | 0.023 | 0.32 | 0.021 | 0.41 |
| rs45577732 | 61983934 | G | 0.025 | 0.28 | 0.0093 | 0.72 |
| rs6011779 | 61984317 | C | 0.0067 | 0.66 | 0.026 | 0.13 |
| rs144298540 | 61984931 | A | -0.017 | 0.58 | 0.022 | 0.54 |
| rs4809542 | 61986787 | A | 0.017 | 0.49 | 0.015 | 0.59 |
| rs2273500 | 61986949 | T | -0.0088 | 0.61 | 0.030 | 0.12 |
| rs4809543 | 61986950 | C | 0.018 | 0.43 | 0.020 | 0.45 |
| rs45449494 | 61987930 | T | 0.021 | 0.35 | 0.021 | 0.41 |
| rs201806007 | 61988398 | C | -0.0052 | 0.77 | 0.023 | 0.26 |
| rs45623037 | 61989658 | G | 0.023 | 0.30 | 0.020 | 0.42 |
| rs45497800 | 61991833 | G | 0.019 | 0.40 | 0.021 | 0.40 |
| rs151176846 | 61997500 | AT | 0.017 | 0.48 | 0.0047 | 0.86 |

**Supplementary Table 5**. *CHRNA4* SNPs and indels associated with nicotine dependence at genome-wide association study meta-analysis *P*<5×10^-5^ and tested for independent replication. Unless otherwise stated, results were taken from a linear regression model in each replication sample with categorical nicotine dependence (mild, moderate, and severe) as the outcome, SNP/indel genotype dosage as the predictor, and age, sex, and principal component eigenvectors as covariates. SNPs and indels are sorted by the meta-analysis *P* values.

| **SNP / indel** | **Minor allele** | **FTC^1^**  **(N=2,374)** | | | **Yale-Penn**  **(N=2,116)** | | | **UW-TTURC (N=1,534)** | | | **GAIN**  **(N=774)** | | | **nonGAIN**  **(N=671)** | | | **Meta-analysis**  ***P*** |
| --- | --- | --- | --- | --- | --- | --- | --- | --- | --- | --- | --- | --- | --- | --- | --- | --- | --- |
|  |  | **info** | **β** | ***P*** | **info** | **β** | ***P*** | **info** | **β** | ***P*** | **info** | **β** | ***P*** | **info** | **β** | ***P*** |  |
| rs6011779 | C | 0.95 | 0.11 | 0.014 | 0.85 | 0.055 | 0.050 | 1.00 | 0.064 | 0.038 | 0.77 | 0.052 | 0.37 | 0.78 | -0.029 | 0.62 | 5.5×10^-4^ |
| rs2273500 | C | 0.94 | 0.12 | 0.013 | 0.98 | 0.066 | 0.025 | 1.00 | 0.054 | 0.12 | 0.80 | 0.048 | 0.46 | 0.82 | -0.036 | 0.58 | 9.2×10^-4^ |
| rs6062901 | G | 0.95 | 0.090 | 0.11 | 0.85 | 0.060 | 0.039 | 0.99 | 0.053 | 0.10 | 0.80 | 0.050 | 0.41 | 0.79 | -0.019 | 0.76 | 2.1×10^-3^ |
| rs6062899 | G | 0.95 | 0.090 | 0.061 | 0.85 | 0.058 | 0.047 | 0.99 | 0.053 | 0.11 | 0.80 | 0.050 | 0.41 | 0.79 | -0.018 | 0.76 | 2.4×10^-3^ |
| rs201806007 | AT | 0.92 | 0.12 | 0.011 | 0.74 | 0.044 | 0.18 | 0.96 | 0.061 | 0.082 | 0.77 | 0.040 | 0.54 | 0.78 | -0.045 | 0.49 | 5.3×10^-3^ |
| rs45449494 | G | 0.93 | 0.11 | 0.12 | 0.95 | 0.027 | 0.51 | 1.00 | 0.059 | 0.20 | 0.69 | 0.047 | 0.62 | 0.69 | -0.056 | 0.56 | 0.059 |
| rs4809543 | A | 0.93 | 0.11 | 0.048 | 0.95 | 0.028 | 0.50 | 1.00 | 0.055 | 0.23 | 0.69 | 0.053 | 0.58 | 0.70 | -0.069 | 0.48 | 0.067 |
| rs45577732 | G | 0.92 | 0.099 | 0.11 | 0.83 | 0.024 | 0.57 | 1.00 | 0.059 | 0.20 | 0.69 | 0.055 | 0.57 | 0.70 | -0.058 | 0.54 | 0.074 |
| rs45461993 | A | 0.9. | 0.11 | 0.061 | 0.84 | 0.016 | 0.70 | 1.00 | 0.058 | 0.21 | 0.69 | 0.036 | 0.70 | 0.70 | -0.053 | 0.58 | 0.088 |
| rs45623037 | C | 0.93 | 0.11 | 0.062 | 0.92 | 0.011 | 0.79 | 0.99 | 0.059 | 0.20 | 0.68 | 0.049 | 0.61 | 0.69 | -0.058 | 0.55 | 0.092 |
| rs151176846 | C | 0.91 | 0.12 | 0.078 | 0.80 | 0.00047 | 0.99 | 0.99 | 0.062 | 0.17 | 0.67 | 0.058 | 0.55 | 0.66 | -0.075 | 0.45 | 0.12 |
| rs45497800 | T | 0.93 | 0.093 | 0.15 | 0.88 | 0.0093 | 0.83 | 0.98 | 0.057 | 0.21 | 0.68 | 0.017 | 0.86 | 0.68 | -0.068 | 0.48 | 0.16 |
| rs4809542 | G | 0.91 | 0.088 | 0.26 | 0.92 | 0.036 | 0.42 | 0.99 | 0.048 | 0.31 | 0.70 | -0.059 | 0.57 | 0.72 | -0.068 | 0.49 | 0.19 |
| rs4809292 | G | 0.93 | 0.082 | 0.38 | 0.79 | 0.029 | 0.58 | 1.00 | 0.037 | 0.51 | 0.75 | -0.090 | 0.43 | 0.76 | -0.045 | 0.66 | 0.35 |
| rs45618935 | A | 0.93 | 0.083 | 0.18 | 0.79 | 0.029 | 0.57 | 0.99 | 0.035 | 0.54 | 0.75 | -0.088 | 0.44 | 0.76 | -0.047 | 0.65 | 0.35 |
| rs4809294 | A | 0.94 | 0.081 | 0.21 | 0.79 | 0.030 | 0.57 | 1.00 | 0.031 | 0.59 | 0.77 | -0.079 | 0.49 | 0.78 | -0.058 | 0.57 | 0.38 |
| rs199666656 | T | 0.94 | 0.069 | 0.063 | 0.78 | 0.034 | 0.51 | 0.99 | 0.033 | 0.56 | 0.76 | -0.095 | 0.40 | 0.77 | -0.044 | 0.67 | 0.39 |
| rs45508092 | G | 0.94 | 0.068 | 0.22 | 0.77 | 0.032 | 0.53 | 1.00 | 0.036 | 0.54 | 0.78 | -0.098 | 0.38 | 0.78 | -0.047 | 0.65 | 0.40 |
| rs45612034 | A | 0.94 | 0.069 | 0.37 | 0.78 | 0.033 | 0.51 | 1.00 | 0.029 | 0.61 | 0.77 | -0.096 | 0.39 | 0.78 | -0.043 | 0.67 | 0.42 |
| rs45456294 | G | 0.94 | 0.069 | 0.86 | 0.78 | 0.033 | 0.52 | 1.00 | 0.028 | 0.62 | 0.77 | -0.097 | 0.39 | 0.78 | -0.043 | 0.67 | 0.43 |
| rs4809293 | A | 0.92 | 0.056 | 0.41 | 0.74 | 0.0057 | 0.93 | 0.99 | 0.029 | 0.62 | 0.75 | -0.13 | 0.26 | 0.76 | -0.042 | 0.69 | 0.75 |
| rs45470098 | A | 0.91 | 0.064 | 0.43 | 0.76 | -0.0084 | 0.90 | 0.94 | 0.037 | 0.58 | 0.78 | -0.20 | 0.13 | 0.79 | -0.050 | 0.66 | 0.82 |
| rs144298540 | T | 0.90 | 0.013 | 0.67 | 0.79 | 0.0041 | 0.93 | 0.90 | 0.050 | 0.43 | 0.67 | -0.046 | 0.72 | 0.70 | -0.12 | 0.29 | 0.88 |

UTR, untranslated region

^1^Rather than implementing a regression model, standardized residuals of the categorical FTND phenotype, regressed against SNP dosages, age, sex, and the 10 first principal components (calculated from chromosome 20 genotype data), were used in QFAM association test in PLINK.

**Supplementary Table 6**. Heterogeneity tests for *CHRNA4* SNPs and indels associated with nicotine dependence at genome-wide association study (GWAS) meta-analysis *P*<5×10^-5^ and followed up for independent replication testing. SNPs and indels are sorted by meta-analysis *P* values across all samples. The *I^2^* index, which quantifies the degree of heterogeneity in a meta-analysis, and the corresponding *P* values were generated using METAL software.

| **SNP / indel** | **Meta-analysis *P* across all samples** | **Heterogeneity tests across GWAS samples** | | **Heterogeneity tests across replication samples** | | **Heterogeneity tests across all samples** | |
| --- | --- | --- | --- | --- | --- | --- | --- |
|  |  | ***I^2^*** | ***P*** | ***I^2^*** | ***P*** | ***I^2^*** | ***P*** |
| rs2273500 | 8.0×10^-9^ | 0 | 0.85 | 0 | 0.42 | 0 | 0.79 |
| rs6011779 | 1.4×10^-8^ | 0 | 0.45 | 0 | 0.43 | 0 | 0.55 |
| rs6062901 | 5.2×10^-8^ | 33 | 0.20 | 0 | 0.71 | 0 | 0.52 |
| rs6062899 | 8.6×10^-8^ | 33.6 | 0.20 | 0 | 0.72 | 0 | 0.51 |
| rs4809543 | 1.3×10^-7^ | 0 | 0.99 | 0 | 0.59 | 0 | 0.85 |
| rs45449494 | 1.7×10^-7^ | 0 | 0.20 | 0 | 0.63 | 0 | 0.92 |
| rs45577732 | 1.9×10^-7^ | 0 | 0.54 | 0 | 0.67 | 0 | 0.88 |
| rs201806007 | 2.1×10^-7^ | 0 | 0.49 | 13.0 | 0.33 | 0 | 0.53 |
| rs151176846 | 3.4×10^-7^ | 0 | 0.72 | 0 | 0.42 | 0 | 0.69 |
| rs45623037 | 3.5×10^-7^ | 0 | 0.16 | 0 | 0.52 | 0 | 0.85 |
| rs4809294 | 3.8×10^-7^ | 0 | 0.70 | 0 | 0.67 | 0 | 0.49 |
| rs4809542 | 7.0×10^-7^ | 0 | 0.57 | 0 | 0.61 | 0 | 0.73 |
| rs45618935 | 7.3×10^-7^ | 0 | 0.64 | 0 | 0.68 | 0 | 0.46 |
| rs45461993 | 8.4×10^-7^ | 0 | 1.00 | 0 | 0.59 | 0 | 0.90 |
| rs4809292 | 8.9×10^-6^ | 0 | 0.64 | 0 | 0.67 | 0 | 0.49 |
| rs199666656 | 1.6×10^-6^ | 0 | 0.61 | 0 | 0.71 | 0 | 0.48 |
| rs45497800 | 2.0×10^-6^ | 0 | 0.99 | 0 | 0.59 | 0 | 0.85 |
| rs45456294 | 3.2×10^-6^ | 0 | 0.65 | 0 | 0.71 | 0 | 0.49 |
| rs45508092 | 3.2×10^-6^ | 0 | 0.61 | 0 | 0.69 | 0 | 0.45 |
| rs4809293 | 3.4×10^-6^ | 0 | 0.61 | 0 | 0.67 | 28.6 | 0.18 |
| rs45612034 | 5.0×10^-6^ | 0 | 0.65 | 0 | 0.71 | 0 | 0.53 |
| rs45470098 | 2.1×10^-5^ | 0 | 0.63 | 0 | 0.45 | 39.7 | 0.093 |
| rs144298540 | 1.3×10^-4^ | 0 | 0.90 | 0 | 0.75 | 6.3 | 0.38 |

**Supplementary Table 7**. Sample-specific associations of the rs2273500-C allele with each of the six specific Fagerström Test for Nicotine Dependence (FTND) items. The items were tested separately in regression models with rs2273500 genotype dosage as the predictor and age, sex, principal component eigenvectors (if applicable), and other sample-specific variables (if applicable) as covariates.

| **Study sample^1^** | **Item 1:**  **How soon after you wake up do/did you smoke your first cigarette?** | | **Item 2:**  **Do/Did you find it difficult to refrain from smoking in places where it is forbidden?** | | **Item 3:**  **Which cigarette would you hate most to give up?** | | **Item 4:**  **How many cigarettes per day do/did you smoke?** | | **Item 5:**  **Do/did you smoke more frequently during the first hours after waking than during the rest of the day?** | | **Item 6:**  **Do/did you smoke if you are so ill that you are in bed most of the day?** | |
| --- | --- | --- | --- | --- | --- | --- | --- | --- | --- | --- | --- | --- |
|  | **β** | ***P*** | **β** | ***P*** | **β** | ***P*** | **β** | ***P*** | **β** | ***P*** | **β** | ***P*** |
| *GWAS meta-analysis samples* | | | | | | | | | | | | |
| deCODE^2^ | 0.11 | 5.7×10^-4^ | 0.060 | 0.43 | 0.26 | 5.6×10^-4^ | 0.089 | 9.6×10^-4^ | 0.053 | 0.47 | 0.25 | 1.9×10^-4^ |
| EAGLE | 0.044 | 0.29 | 0.098 | 0.23 | 0.075 | 0.32 | 0.015 | 0.69 | 0.10 | 0.31 | 0.058 | 0.49 |
| COPDGene | 0.092 | 0.053 | 0.066 | 0.531 | 0.11 | 0.27 | 0.033 | 0.35 | 0.12 | 0.20 | -0.076 | 0.46 |
| COGEND | 0.16 | 0.0065 | -0.024 | 0.82 | 0.12 | 0.25 | 0.15 | 0.0038 | 0.19 | 0.070 | 0.12 | 0.24 |
| SAGE* | 0.13 | 0.073 | 0.19 | 0.22 | -0.051 | 0.75 | 0.094 | 0.18 | 0.42 | 0.0066 | 0.32 | 0.042 |
| *Independent replication samples* | | | | | | | | | | | | |
| FTC^3^ | 0.059 | 0.32 | 0.070 | 0.18 | 0.064 | 0.25 | 0.12 | 0.019 | 0.049 | 0.32 | -0.012 | 0.73 |
| Yale-Penn | 0.078 | 0.040 | -0.016 | 0.85 | 0.087 | 0.32 | 0.054 | 0.13 | 0.18 | 0.042 | 0.11 | 0.25 |
| UW-TTURC | 0.065 | 0.13 | 0.18 | 0.12 | 0.030 | 0.77 | 0.017 | 0.65 | 0.18 | 0.067 | 0.11 | 0.28 |
| GAIN | 0.051 | 0.59 | -0.098 | 0.65 | 0.30 | 0.079 | 0.021 | 0.81 | 0.54 | 0.010 | 0.29 | 0.11 |
| nonGAIN | 0.043 | 0.64 | -0.22 | 0.30 | 0.057 | 0.82 | 0.0069 | 0.94 | NA^4^ | NA^4^ | -0.22 | 0.24 |
| Meta-analysis | 0.085 | 2.3×10^-8^ | 0.059 | 0.032 | 0.10 | 2.4×10^-4^ | 0.061 | 5.1×10^-6^ | 0.12 | 4.2×10^-5^ | 0.077 | 5.1×10^-3^ |

NA, not available

^1^For deCODE, only the 4,683 participants with both genotype and specific FTND data available were used for these analyses. Otherwise, all participants from the primary analyses of the discovery and replication samples were used. ^2^Genomic control was applied to each specific FTND item analysis in deCODE to correct for any inflation due to relatedness among sample participants. Genomic control was not applied in the specific FTND item analyses for the other samples, which were all comprised of unrelated participants. ^3^Rather than implementing a regression model, standardized residuals of the categorical FTND phenotype, regressed against SNP dosages, age, sex, and the 10 first principal components (calculated from chromosome 20 genotype data), were used in QFAM association test in PLINK. ^4^The regression model did not converge due to a high number of missing “No” responses. The meta-analysis results for this FTND item reflect the cohorts with available results.

**Supplementary Table 8.** Sample-specific associations of the rs16969968-A allele with each of the six specific Fagerström Test for Nicotine Dependence (FTND) items. The items were tested separately in regression models with rs16969968 genotype dosage as the predictor and age, sex, principal component eigenvectors (if applicable), and other sample-specific variables (if applicable) as covariates.

| **Study sample^1^** | **Item 1:**  **How soon after you wake up do/did you smoke your first cigarette?** | | **Item 2:**  **Do/Did you find it difficult to refrain from smoking in places where it is forbidden?** | | **Item 3:**  **Which cigarette would you hate most to give up?** | | **Item 4:**  **How many cigarettes per day do/did you smoke?** | | **Item 5:**  **Do/did you smoke more frequently during the first hours after waking than during the rest of the day?** | | **Item 6:**  **Do/did you smoke if you are so ill that you are in bed most of the day?** | |
| --- | --- | --- | --- | --- | --- | --- | --- | --- | --- | --- | --- | --- |
|  | **β** | ***P*** | **β** | ***P*** | **β** | ***P*** | **β** | ***P*** | **β** | ***P*** | **β** | ***P*** |
| *GWAS meta-analysis samples* | | | | | | | | | | | | |
| deCODE^2^ | 0.092 | 2.3×10^-5^ | 0.043 | 0.41 | 0.091 | 0.061 | 0.10 | 1.2×10^-7^ | 0.023 | 0.64 | 0.23 | 1.8×10^-7^ |
| EAGLE | 0.13 | 3.4×10^-5^ | 0.048 | 0.43 | 0.066 | 0.24 | 0.12 | 7.6×10^-6^ | -0.011 | 0.88 | 0.16 | 7.8×10^-3^ |
| COPDGene | 0.066 | 0.066 | 0.089 | 0.26 | 0.20 | 9.3×10^-3^ | 0.10 | 1.3×10^-4^ | 0.13 | 0.079 | 0.11 | 0.17 |
| COGEND | 0.17 | 7.0×10^-5^ | 0.19 | 0.012 | 0.28 | 7.9×10^-5^ | 0.20 | 1.5×10^-7^ | 0.25 | 1.3×10^-3^ | 0.17 | 0.018 |
| SAGE* | 0.051 | 0.32 | -0.030 | 0.78 | 0.24 | 0.028 | 0.069 | 0.16 | 0.17 | 0.12 | 0.15 | 0.16 |
| *Independent replication samples* | | | | | | | | | | | | |
| FTC^3^ | 0.092 | 0.039 | 0.064 | 0.13 | 0.088 | 0.068 | 0.062 | 0.16 | 0.065 | 0.11 | 0.082 | 0.039 |
| Yale-Penn | 0.045 | 0.13 | 0.017 | 0.79 | 0.17 | 8.7×10^-3^ | 0.029 | 0.26 | 0.22 | 1.5×10^-3^ | 0.025 | 0.72 |
| UW-TTURC | 0.060 | 0.069 | 0.054 | 0.56 | 0.069 | 0.39 | 0.088 | 2.2×10^-3^ | -0.063 | 0.42 | -0.039 | 0.62 |
| GAIN | 0.098 | 0.095 | 0.18 | 0.16 | -0.014 | 0.90 | 0.17 | 1.7×10^-3^ | 0.087 | 0.54 | 0.21 | 0.073 |
| nonGAIN | 0.061 | 0.32 | 0.037 | 0.78 | 0.073 | 0.66 | 0.11 | 0.073 | NA^4^ | NA^4^ | 0.29 | 0.016 |
| Meta-analysis | 0.086 | 7.8×10^-15^ | 0.063 | 2.4×10^-3^ | 0.12 | 1.3×10^-8^ | 0.10 | 9.4×10^-24^ | 0.081 | 2.2×10^-4^ | 0.13 | 1.8×10^-10^ |

NA, not available

^1^For deCODE, only the 4,833 participants with specific FTND data available were used for these analyses. All COPDGene, COGEND, and SAGE* participants were used. ^2^Genomic control was applied to each specific FTND item analysis in deCODE to correct for any inflation due to relatedness among sample participants. Genomic control was not applied in the specific FTND item analyses for the other samples, which were all comprised of unrelated participants. ^3^Rather than implementing a regression model, standardized residuals of the categorical FTND phenotype, regressed against SNP dosages, age, sex, and the 10 first principal components (calculated from chromosome 20 genotype data), were used in QFAM association test in PLINK. ^4^The regression model did not converge due to a high number of missing “No” responses. The meta-analysis results for this FTND item reflect the cohorts with available results.

**Supplementary Table 9**. SNP association results for cigarettes per day from the Tobacco and Genetics Consortium meta-analysis (available at http://www.med.unc.edu/pgc/downloads). The 57 HapMap phase II-imputed SNPs located from 50kb upstream to 50kb downstream of the *CHRNA4* gene are sorted by the meta-analysis *P* value. Linkage disequilibrium with our lead SNP rs2273500 in the 1000 Genomes EUR reference panel is also presented.

| **SNP** | **Base pair position**  **(NCBI build 37)** | **Distance to rs2273500** | **D’ with rs2273500** | **r^2^ with rs2273500** | **Allele1** | **Allele2** | **Allele1 frequency** | **β** | **SE** | **Meta-analysis *P*** |
| --- | --- | --- | --- | --- | --- | --- | --- | --- | --- | --- |
| rs4809528 | 61943661 | 43288 | 0.67 | 0.19 | T | C | 0.065 | 0.88 | 0.31 | 4.8×10^-3^ |
| rs6090392 | 62008975 | 22026 | 0.24 | 0.015 | T | G | 0.38 | -0.23 | 0.10 | 0.027 |
| rs6089900 | 62005638 | 6824 | 0.54 | 0.23 | C | G | 0.58 | 0.29 | 0.13 | 0.027 |
| rs3746364 | 62038757 | 51808 | 1.00 | 0.016 | T | C | 0.12 | -0.36 | 0.17 | 0.031 |
| rs911053 | 62012775 | 25826 | 0.091 | 0.002 | C | G | 0.46 | -0.24 | 0.11 | 0.035 |
| rs6122435 | 62022805 | 35856 | 0.69 | 0.022 | T | C | 0.77 | 0.24 | 0.12 | 0.042 |
| rs4522666 | 61974480 | 12469 | 0.26 | 0.021 | A | G | 0.59 | -0.26 | 0.13 | 0.046 |
| rs6011747 | 61964667 | 22282 | 0.74 | 0.22 | A | G | 0.95 | -0.77 | 0.39 | 0.049 |
| rs6089899 | 61999308 | 7156 | 0.88 | 0.61 | A | G | 0.59 | -0.29 | 0.15 | 0.065 |
| rs6122434 | 62014997 | 28048 | 0.72 | 0.024 | A | G | 0.21 | -0.21 | 0.12 | 0.066 |
| rs13040963 | 62027516 | 40567 | 0.49 | 0.011 | A | T | 0.78 | 0.22 | 0.12 | 0.070 |
| rs6090403 | 62032935 | 45986 | 0.26 | 0.011 | T | C | 0.45 | 0.44 | 0.25 | 0.076 |
| rs6011730 | 61947082 | 39867 | 0.67 | 0.19 | T | C | 0.94 | -0.34 | 0.20 | 0.083 |
| rs6011731 | 61948489 | 38460 | 0.67 | 0.19 | A | G | 0.94 | -0.34 | 0.20 | 0.087 |
| rs3746380 | 61947830 | 39119 | 0.67 | 0.19 | T | C | 0.061 | 0.33 | 0.20 | 0.090 |
| rs6090378 | 61975378 | 11571 | 0.98 | 0.010 | A | G | 0.94 | -0.62 | 0.36 | 0.090 |
| rs7347209 | 61941586 | 45363 | 0.67 | 0.19 | T | C | 0.061 | 0.35 | 0.20 | 0.091 |
| rs12481469 | 62031217 | 44268 | 0.061 | 0 | C | G | 0.94 | -0.64 | 0.39 | 0.10 |
| rs3746383 | 61957750 | 29199 | 0.34 | 0.005 | T | G | 0.22 | -0.23 | 0.16 | 0.14 |
| rs2038442 | 62028210 | 41261 | 0.037 | 0 | A | G | 0.51 | -0.15 | 0.11 | 0.15 |
| rs755203 | 61994264 | 7315 | 0.98 | 0.19 | A | G | 0.55 | -0.15 | 0.11 | 0.15 |
| rs6062894 | 61954675 | 32274 | 0.39 | 0.12 | T | C | 0.89 | -0.19 | 0.14 | 0.19 |
| rs735501 | 62000202 | 13253 | 0.61 | 0.012 | T | C | 0.85 | -0.31 | 0.24 | 0.19 |
| rs2038441 | 62028063 | 41114 | 0.025 | 0 | A | C | 0.48 | 0.14 | 0.11 | 0.20 |
| rs4603829 | 61968892 | 18057 | 0.28 | 0.024 | T | C | 0.64 | -0.10 | 0.09 | 0.25 |
| rs6010927 | 62029711 | 42762 | 0.053 | 0.001 | A | G | 0.28 | -0.17 | 0.15 | 0.28 |
| rs9680065 | 61967037 | 19912 | 0.27 | 0.022 | A | G | 0.64 | -0.10 | 0.09 | 0.28 |
| rs2180614 | 61960671 | 26278 | 0.29 | 0.025 | T | C | 0.35 | 0.093 | 0.09 | 0.32 |
| rs4809552 | 62028308 | 41359 | 0.17 | 0.024 | T | C | 0.17 | 0.13 | 0.13 | 0.33 |
| rs6010918 | 61989501 | 2552 | 1.00 | 0.009 | A | G | 0.045 | 0.24 | 0.27 | 0.38 |
| rs3746369 | 62034258 | 47309 | 0.032 | 0 | A | C | 0.046 | 0.46 | 0.54 | 0.40 |
| rs6062897 | 61963973 | 22976 | 0.32 | 0.034 | T | G | 0.65 | -0.081 | 0.10 | 0.40 |
| rs3746381 | 61950792 | 36157 | 0.27 | 0.003 | T | C | 0.20 | -0.12 | 0.15 | 0.42 |
| rs3787138 | 61979224 | 7725 | 0.54 | 0.23 | A | G | 0.88 | -0.12 | 0.15 | 0.44 |
| rs6011776 | 61983756 | 3193 | 0.49 | 0.17 | T | C | 0.88 | -0.12 | 0.16 | 0.44 |
| rs4809525 | 61926823 | 60126 | 0.41 | 0.091 | A | G | 0.091 | 0.12 | 0.17 | 0.46 |
| rs6011805 | 62018747 | 31798 | 0.13 | 0.005 | A | G | 0.37 | 0.069 | 0.10 | 0.50 |
| rs8119606 | 62028353 | 41404 | 0.20 | 0.029 | T | C | 0.089 | -0.15 | 0.24 | 0.51 |
| rs1044394 | 61982085 | 4864 | 1.00 | 0.012 | A | G | 0.060 | 0.40 | 0.64 | 0.53 |
| rs6011770 | 61977431 | 9518 | 1.00 | 0.006 | T | C | 0.030 | 1.34 | 2.50 | 0.59 |
| rs1535243 | 62024275 | 37326 | 0.13 | 0.005 | A | G | 0.37 | 0.052 | 0.10 | 0.61 |
| rs6122438 | 62032671 | 45722 | 0.21 | 0.004 | A | G | 0.36 | 0.12 | 0.24 | 0.63 |
| rs2093107 | 61993942 | 6993 | 1.00 | 0.014 | A | G | 0.071 | -0.10 | 0.21 | 0.64 |
| rs6122425 | 61963292 | 23657 | 0.37 | 0.008 | A | C | 0.24 | -0.050 | 0.11 | 0.65 |
| rs4809538 | 61970176 | 16773 | 0.36 | 0.007 | A | G | 0.22 | -0.050 | 0.11 | 0.66 |
| rs964099 | 62026990 | 40041 | 0.23 | 0.038 | A | G | 0.12 | 0.074 | 0.17 | 0.67 |
| rs12625977 | 62029070 | 42121 | 0.19 | 0.020 | T | C | 0.77 | -0.051 | 0.13 | 0.69 |
| rs6010903 | 61960080 | 26869 | 0.090 | 0.003 | A | G | 0.95 | 0.086 | 0.22 | 0.70 |
| rs755041 | 61945759 | 41190 | 0.29 | 0.039 | A | G | 0.73 | -0.044 | 0.12 | 0.70 |
| rs3746367 | 62035103 | 48154 | 0.080 | 0.002 | T | C | 0.045 | -0.10 | 0.28 | 0.71 |
| rs12625971 | 62029016 | 42067 | 0.16 | 0.012 | T | C | 0.76 | -0.047 | 0.13 | 0.71 |
| rs6011817 | 62039549 | 52600 | 0.013 | 0 | A | G | 0.88 | -0.25 | 0.68 | 0.71 |
| rs6090404 | 62033164 | 46215 | 0.010 | 0 | T | C | 0.93 | 0.053 | 0.26 | 0.84 |
| rs1044396 | 61981134 | 5815 | 1.00 | 0.20 | A | G | 0.55 | -0.030 | 0.18 | 0.87 |
| rs4809526 | 61930017 | 56932 | 0.26 | 0.009 | A | G | 0.43 | -0.015 | 0.10 | 0.88 |
| rs755204 | 61994165 | 7216 | 0.97 | 0.36 | A | G | 0.072 | 0.021 | 0.21 | 0.92 |
| rs7265034 | 61950145 | 36804 | 0.29 | 0.036 | A | C | 0.27 | 0.00090 | 0.18 | 1.00 |

SE, standard error

**Supplementary Table 10**. Counts of split reads in liver and brain tissue for splices originating from exon 4 by rs2273500 genotype. For the liver tissue that showed a significant splicing effect by rs2273500, the percentages of counts inferred for the unobserved C/C genotype are presented.

| **Exon 4 to:** | **Base pair position**  **(NCBI build 37)** | **Liver** | | | | | | **Brain** | | | | |
| --- | --- | --- | --- | --- | --- | --- | --- | --- | --- | --- | --- | --- |
|  |  | **T/T (N=25)** | | **T/C (N=7)** | | **C/C (inferred)** | ***P*** | **T/T (N=256)** | | **T/C (N=136)** | | ***P*** |
|  |  | **Counts** | **%** | **Counts** | **%** | **%** |  | **Counts** | **%** | **Counts** | **%** |  |
| Other | - | 8 | 0.4% | 14 | 1.7% | 3% | 5.4×10^-58^ | 46 | 1.7% | 33 | 2.4% | 0.30 |
| 5 | 61982379 | 1,032 | 52.9% | 374 | 46.2% | 39.4% |  | 2,406 | 88.6% | 1,193 | 87.8% |  |
| 4.2^1^ | 61986106 | 237 | 12.1% | 190 | 23.5% | 34.8% |  | 198 | 7.3% | 107 | 7.9% |  |
| 4.1 + 6bp^2^ | 61986941 | 7 | 0.4% | 79 | 9.8% | 19.1% |  | 1 | 0.0% | 1 | 0.1% |  |
| 4.1 | 61986947 | 667 | 34.2% | 153 | 18.9% | 3.6% |  | 65 | 2.4% | 24 | 1.8% |  |

^1^Exon 4.2 is previously unannotated.

^2^This cryptic splice acceptor in exon 4.1 occurs 6 base pairs downstream of the regular 4.1 splice acceptor

**Supplementary Table 11**. Associations of the two top nicotine dependence-associated *CHRNA4* SNPs with overall lung cancer and two lung cancer sub-types in meta-analyses across six case-control samples (IARC, ICR, MDACC, NCI, SLRI, and HGF). SNP associations were adjusted for age, sex, and principal components.

| **SNP** | **Minor allele** | **MAF^1^** | **Meta-analysis of**  **all lung cancer**  **(total N=28,998)** | | **Meta-analysis of**  **lung adenocarcinoma**  **(total N=19,589)** | | **Meta-analysis of**  **squamous lung cancer**  **(total N=19,437)** | |
| --- | --- | --- | --- | --- | --- | --- | --- | --- |
|  |  |  | **OR (95% CI)** | ***P*** | **OR (95% CI)** | ***P*** | **OR (95% CI)** | ***P*** |
| rs6011779 | C | 0.21 | 1.06 (1.01-1.11) | 0.013 | 1.02 (0.95-1.10) | 0.52 | 1.09 (1.01-1.17) | 0.025 |
| rs2273500 | C | 0.16 | 1.06 (1.00-1.12) | 0.036 | 1.02 (0.94-1.11) | 0.66 | 1.09 (1.00-1.19) | 0.046 |

CI, confidence interval; HGF, Helmholtz-Gemeinschaft Deutscher Forschungszentren in Germany; IARC, International Agency for Research on Cancer; ICR, Institute of Cancer Research; MAF, minor allele frequency; MDACC, MD Anderson Cancer Center; NCI, MD Anderson Cancer Center; OR, odds ratio; SLRI, Samuel Lunenfeld Research Institute in Toronto

^1^MAF was weighted by sample size across the six lung cancer case-control samples.

**Supplementary Table 12**. Associations of the two top nicotine dependence-associated *CHRNA4* SNPs with overall lung cancer in meta-analyses across a subset of three case-control samples (IARC, MDACC, and NCI). SNP associations were all adjusted for age, sex, and principal components, and follow-up testing included additional adjustment for smoking history (ever vs. never) and pack-years of smoking among ever smokers (0 for never smokers).

| **SNP** | **Minor allele** | **Meta-analysis of all lung cancer without adjustment for smoking**  **(total N=12,084)** | | **Meta-analysis of all lung cancer with adjustment for smoking**  **(total N=11,785)** | |
| --- | --- | --- | --- | --- | --- |
|  |  | **OR (95% CI)** | ***P*** | **OR (95% CI)** | ***P*** |
| rs6011779 | C | 1.07 (1.00-1.15) | 0.040 | 1.04 (0.97-1.12) | 0.30 |
| rs2273500 | C | 1.07 (0.99-1.17) | 0.090 | 1.03 (0.94-1.13) | 0.51 |

CI, confidence interval; IARC, International Agency for Research on Cancer; MAF, minor allele frequency; MDACC, MD Anderson Cancer Center; NCI, MD Anderson Cancer Center; OR, odds ratio

^1^MAF was weighted by sample size across the six lung cancer case-control samples.

**Supplementary Figure 1.** Known *CHRNA4* gene transcripts from the UCSC Genome Browser and median read coverage (normalized by total number of paired reads genome-wide) per liver sample from the Genotype-Tissue Expression (GTEx) project. Exon 4.1 designates the ancillary exon containing rs2273500 at the splice site acceptor, and exon 4.2 designates a previously unannotated exon that starts at base pair 61,986,106 (NCBI build 37).


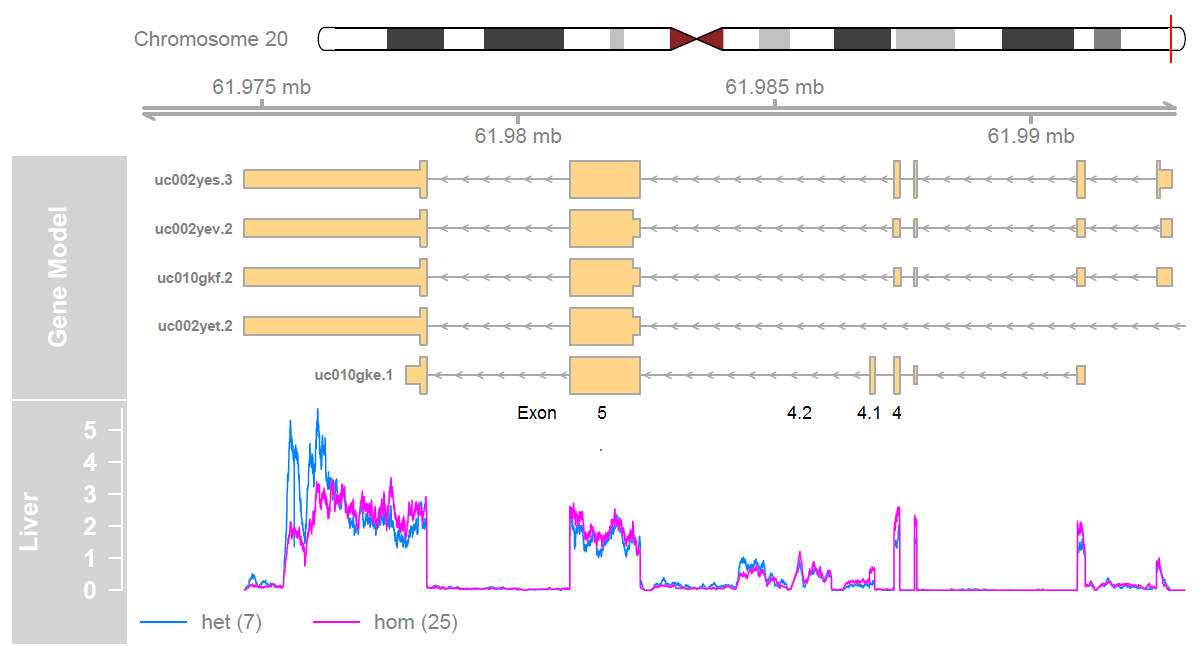


**Supplementary Figure 2**. Quantile-quantile plot for the genome-wide association meta-analysis of 9.9 million SNPs and indels with nicotine dependence. The plots compare the observed vs. expected *P* values for the meta-analysis of five samples, totaling 17,079 participants of European-ancestry. Genomic control was applied to the sample-specific association results prior to combining in the meta-analysis.


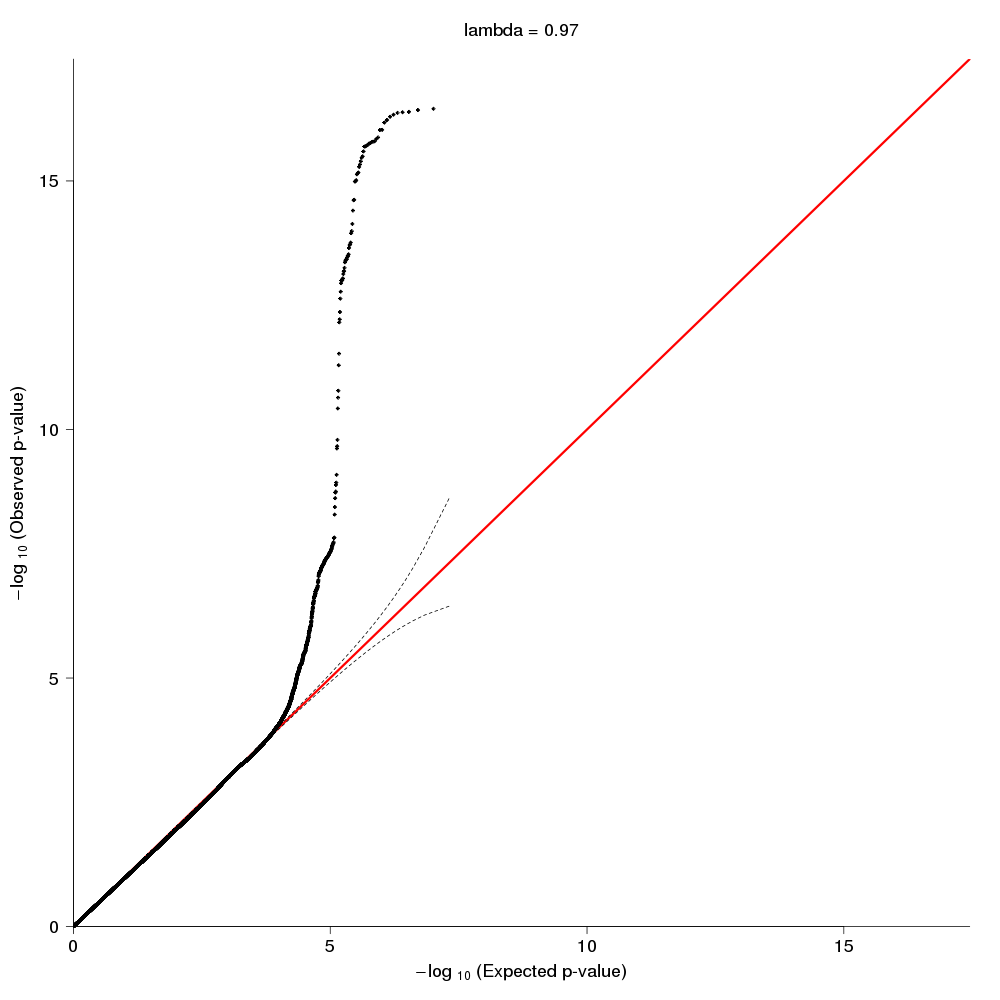


λ_gc_=0.97

**Supplementary Figure 3.** Linkage disequilibrium structure among our newly identified SNPs (marked by asterisks [*]) and other *CHRNA4* SNPs previously reported for association with nicotine dependence and other smoking behaviors. (A) D’ values and (B) r^2^ values are shown with reference to the 1000 Genomes populations of European-ancestry (denoted EUR). Pink to red shading for D’ and grey to black shading for r^2^ indicate lower to higher correlation values. For D’ only, purple shading indicates little statistical support for the observed value.

(A)


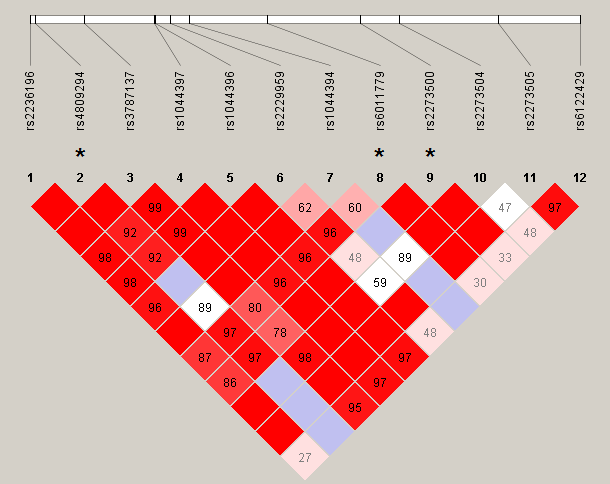


(B)


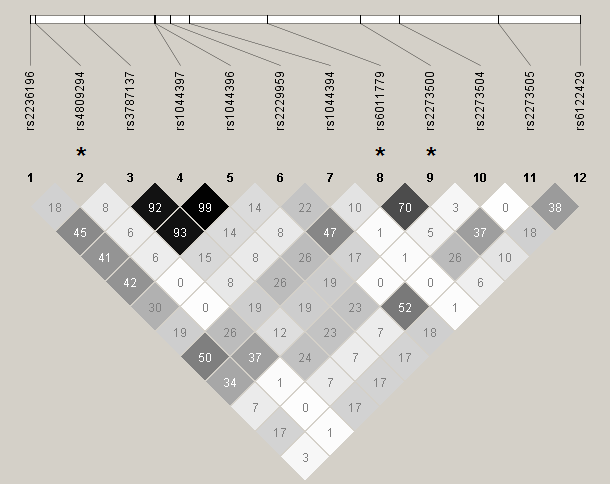


**Supplementary Figure 4**. Associations of the two top nicotine dependence-associated SNPs with transcript-level *CHRNA4* mRNA expression across 10 different human brain regions taken from 134 European-ancestry participants in the UK Brain Expression Consortium.


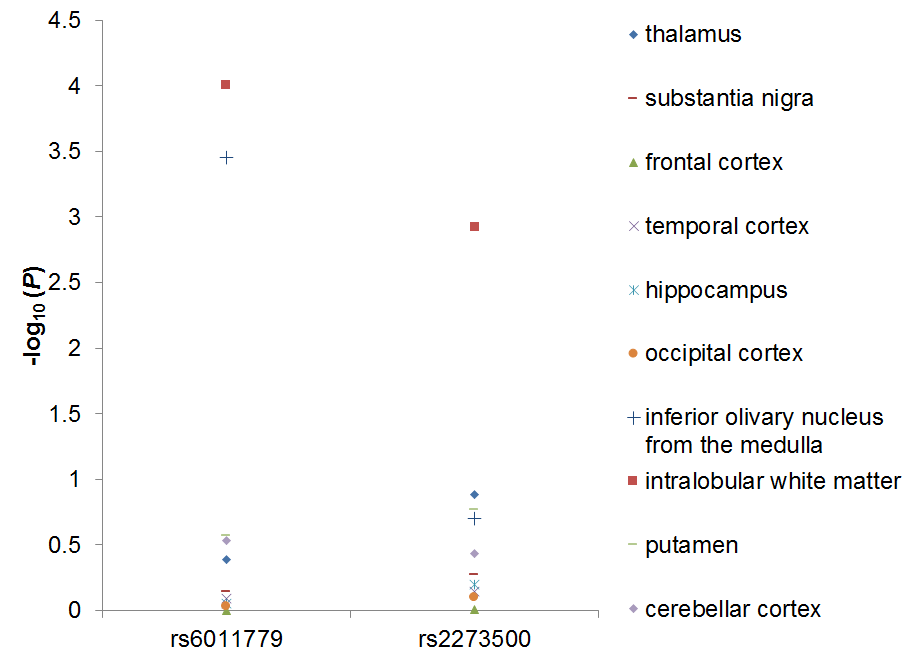


**Acknowledgements**

The deCODE work was supported in part by NIDA by grants R01 DA035825 (Principal Investigator [PI]: Dana Hancock) and R01 DA017932 (PI: Kari Stefansson). deCODE thanks the participants in the genetic studies whose contributions made this work possible.

Funding support for the Environment and Genetics in Lung Cancer Etiology (EAGLE) study was provided through the NIH GEI (Z01 CP 010200). The human participants participating in this study and its companion “Prostate, Lung Colon and Ovary Screening Trial” are supported by intramural resources of the NCI. Assistance with phenotype harmonization and genotype cleaning, as well as with general study coordination, was provided by the GENEVA Coordinating Center (U01 HG004446). Assistance with data cleaning was provided by the National Center for Biotechnology Information. Funding support for genotyping, which was performed at The Johns Hopkins University’s Center for Inherited Disease Research (CIDR), was provided by the NIH GEI (U01 HG004438). The datasets used for the analyses described in this manuscript were obtained from the database of Genotypes and Phenotypes (dbGaP, http://www.ncbi.nlm.nih.gov/gap) through accession number phs000093.vs.p2.

The COPDGene^®^ project was supported by award numbers R01 HL089897 and R01 HL089856 from the National Heart, Lung, and Blood Institute (NHLBI). Research reported in this publication was also supported by the NHLBI award number K01 HL125858. The content is solely the responsibility of the authors and does not necessarily represent the official views of the National Institutes of Health. The content is solely the responsibility of the authors and does not necessarily represent the official views of NHLBI or NIH. The COPDGene^®^ project is also supported by the COPD Foundation through contributions made to an Industry Advisory Board comprised of AstraZeneca, Boehringer Ingelheim, Novartis, Pfizer, Siemens, Sunovion, and GlaxoSmithKline. The authors acknowledge investigators of the COPDGene^®^ project core units: Administrative (James Crapo [Principal Investigator] and Edwin Silverman [Principal Investigator]), Barry Make, and Elizabeth Regan); Genetic Analysis (Terri Beaty, Nan Laird, Christoph Lange, Michael Cho, Stephanie Santorico, Dawn DeMeo, Nadia Hansel, Craig Hersh, Peter Castaldi, Merry-Lynn McDonald, Emily Wan, Megan Hardin, Jacqueline Hetmanski, Margaret Parker, Marilyn Foreman, Brian Hobbs, Robert Busch, Adel El-Bouiez, Megan Hardin, Dandi Qiao, Elizabeth Regan, Eitan Halper-Stromberg, Ferdouse Begum, Sungho Won); Imaging (David Lynch, Harvey Coxson, MeiLan Han, Eric Hoffman, Stephen Humphries Francine Jacobson, Philip Judy, Ella Kazerooni, John Newell, Jr., Elizabeth Regan, James Ross, Raul San Jose Estepar, Berend Stoel, Juerg Tschirren, Eva van Rikxoort, Bram van Ginneken, George Washko, Carla Wilson, Mustafa Al Qaisi, Teresa Gray, Alex Kluiber, Tanya Mann, Jered Sieren, Douglas Stinson, Joyce Schroeder, Edwin Van Beek); Pulmonary Function Testing Quality Assurance (Robert Jensen); Data Coordinating Center and Biostatistics (Douglas Everett, Anna Faino, Matt Strand, Carla Wilson); and Epidemiology (Jennifer Black-Shinn, Gregory Kinney, Katherine Pratte). The authors also acknowledge the clinical center investigators: Jeffrey Curtis, Carlos Martinez, Perry G. Pernicano, Nicola Hanania, Philip Alapat, Venkata Bandi, Mustafa Atik, Aladin Boriek, Kalpatha Guntupalli, Elizabeth Guy, Amit Parulekar, Arun Nachiappan, Dawn DeMeo, Craig Hersh, George Washko, Francine Jacobson, R. Graham Barr, Byron Thomashow, John Austin, Belinda D’Souza, Gregory D.N. Pearson, Anna Rozenshtein, Neil MacIntyre, Jr., Lacey Washington, H. Page McAdams, Charlene McEvoy, Joseph Tashjian, Robert Wise, Nadia Hansel, Robert Brown, Karen Horton, Nirupama Putcha, Richard Casaburi, Alessandra Adami, Janos Porszasz, Hans Fischer, Matthew Budoff, Dan Cannon, Harry Rossiter, Amir Sharafkhaneh, Charlie Lan, Christine Wendt, Brian Bell, Marilyn Foreman, Gloria Westney, Eugene Berkowitz, Russell Bowler, David Lynch, Richard Rosiello, David Pace, Gerard Criner, David Ciccolella, Francis Cordova, Chandra Dass, Robert D’Alonzo, Parag Desai, Michael Jacobs, Steven Kelsen, Victor Kim, A. James Mamary, Nathaniel Marchetti, Aditti Satti, Kartik Shenoy, Robert M. Steiner, Alex Swift, Irene Swift, Gloria Vega-Sanchez, Mark Dransfield, William Bailey, J. Michael Wells, Surya Bhatt, Hrudaya Nath, Joe Ramsdell, Paul Friedman, Xavier Soler, Andrew Yen, Alejandro Cornellas, John Newell, Jr., Brad Thompson, MeiLan Han, Ella Kazerooni, Fernando Martinez, Joanne Billings, Tadashi Allen, Frank Sciurba, Divay Chandra, Joel Weissfeld, Carl Fuhrman, Jessica Bon, Antonio Anzueto, Sandra Adams, Diego Maselli-Caceres, and Mario Ruiz.

COGEND was supported by grants from the National Cancer Institute (NCI; grant number P01 CA089392, PI: Laura Bierut) and NIDA (R01 DA036583 and R01 DA025888, PI: Laura Bierut), both of NIH. The COGEND genotype data are available via dbGaP as part of the “Genetic Architecture of Smoking and Smoking Cessation” (accession number phs000404.v1.p1) and “Study of Addiction: Genetics and Environment (SAGE)” (accession number phs000092.v1.p1). Funding support for genotyping, which was performed at CIDR, was provided by 1 X01 HG005274-01 and by the NIH Genes, Environment and Health Initiative [GEI] (U01 HG004422). CIDR is fully funded through a federal contract from the NIH to The Johns Hopkins University, contract number HHSN268200782096C. Assistance with genotype cleaning, as well as with general study coordination, was provided by the GENEVA Coordinating Center (U01 HG004446).

Funding support for SAGE was further provided through the NIH GEI (U01 HG004422). Assistance with phenotype harmonization and genotype cleaning, as well as with general study coordination, was provided by the GENEVA Coordinating Center (U01 HG004446). Assistance with data cleaning was provided by the National Center for Biotechnology Information. Support for collection of datasets and samples was provided by the Collaborative Study on the Genetics of Alcoholism (COGA; U10 AA008401) and the Family Study of Cocaine Dependence (FSCD; R01 DA013423, PI: Laura Bierut). Funding support for genotyping, which was performed at CIDR, was provided by the NIH GEI (U01HG004438), the National Institute on Alcohol Abuse and Alcoholism (NIAAA), NIDA, and the NIH contract "High throughput genotyping for studying the genetic contributions to human disease" (HHSN268200782096C). The datasets used for the analyses described in this manuscript were obtained via dbGaP accession number phs000092.v1.p1.

The authors warmly thank the participating twin pairs and their family members of the Finnish Twin Cohort Study for their contribution. We would like to express our appreciation to the skilled study interviewers A-M Iivonen, K Karhu, H-M Kuha, U Kulmala-Gråhn, M Mantere, K Saanakorpi, M Saarinen, R Sipilä, L Viljanen, and E Voipio. Anja Häppölä and Kauko Heikkilä are acknowledged for their valuable contribution in recruitment, data collection, and data management. Phenotyping and genotyping of the Finnish twin cohorts was supported by the Academy of Finland Center of Excellence in Complex Disease Genetics (grants 213506, 129680), the Academy of Finland (grants 100499, 205585, 118555, 141054, 265240, 263278 and 264146 to Jaakko Kaprio), NIH/NIAAA (grant numbers R37 AA012502, K05 AA000145, and R01 AA009203 to R. J. Rose and R01 AA015416 and K02 AA018755 to D. M. Dick), NIH/NIDA (grant number R01 DA12854, PI: Pamela A. Madden), Sigrid Juselius Foundation (to Jaakko Kaprio), Global Research Award for Nicotine Dependence, Pfizer Inc. (to Jaakko Kaprio), and the Welcome Trust Sanger Institute, UK. Antti-Pekka Sarin and Samuli Ripatti are acknowledged for genotype data quality controls and imputation. Association analyses were run at the ELIXIR Finland node hosted at CSC – IT Center for Science for ICT resources.

Funding support for the Genetic Association Information Network (GAIN) Genome-Wide Association of Schizophrenia Study was provided by the National Institute of Mental Health (NIMH) (R01 MH67257, R01 MH59588, R01 MH59571, R01 MH59565, R01 MH59587, R01 MH60870, R01 MH59566, R01 MH59586, R01 MH61675, R01 MH60879, R01 MH81800, U01 MH46276, U01 MH46289 U01 MH46318, U01 MH79469, and U01 MH79470) and the genotyping of samples was provided through GAIN. The datasets used for the analyses described in this manuscript were obtained from dbGaP through accession number phs000021.v3.p2. Samples and associated phenotype data for the Genome-Wide Association of Schizophrenia Study were provided by the Molecular Genetics of Schizophrenia Collaboration (PI: Pablo V. Gejman, Evanston Northwestern Healthcare (ENH) and Northwestern University, Evanston, IL, USA).

Funding support for the Molecular Genetics of Schizophrenia - nonGAIN Sample, was provided by Genomics Research Branch at NIMH, and the genotyping and analysis of samples was also provided through GAIN and under the MGS U01s: MH79469 and MH79470. Assistance with data cleaning was provided by NCBI. The dataset used for the analyses described in this manuscript were obtained via dbGaP accession number phs000167.v1.p1. Samples and associated phenotype data for the nonGAIN study were collected under the following grants: NIMH Schizophrenia Genetics Initiative U01s: MH46276 (CR Cloninger), MH46289 (C Kaufmann), and MH46318 (MT Tsuang); and MGS Part 1 (MGS1) and Part 2 (MGS2) R01s: MH67257 (NG Buccola), MH59588 (BJ Mowry), MH59571 (PV Gejman), MH59565 (Robert Freedman), MH59587 (F Amin), MH60870 (WF Byerley), MH59566 (DW Black), MH59586 (JM Silverman), MH61675 (DF Levinson), and MH60879 (CR Cloninger). Further details of collection sites, individuals, and institutions may be found in data supplement Table 1 of Sanders et al. (2008; PMID: 18198266) and at the study dbGaP pages.

The University of Wisconsin-Transdisciplinary Tobacco Use Research Center (UW-TTURC) sample was accessed via dbGaP as part of “Genetic Architecture of Smoking and Smoking Cession” (accession number phs000404.v1.p1). Funding support for collection of the UW-TTURC dataset and samples was provided by P50 DA019706 and P50 CA084724. Funding support for genotyping, which was performed at CIDR, was provided by 1 X01 HG005274-01. CIDR is fully funded through a federal contract from NIH to The Johns Hopkins University, contract number HHSN268200782096C. Assistance with genotype cleaning, as well as with general study coordination, was provided by the GENEVA Coordinating Center (U01 HG004446).

The Yale-Penn study was supported by NIH grants RC2 DA028909 (PI: Joel Gelernter), R01 DA12690 (PI: Joel Gelernter), R01 DA12849 (PI: Joel Gelernter), R01 DA18432 (PI: Henry Kranzler), R01 AA11330 (PI: Joel Gelernter), R01 AA017535 (PI: Joel Gelernter), and the VA Connecticut and Philadelphia VA MIRECCs. Genotyping services for a part of the Yale-Penn GWAS were provided by CIDR and Yale University (Center for Genome Analysis). CIDR is fully funded through a federal contract from NIH to The Johns Hopkins University (contract number N01-HG-65403). We are grateful to Ann Marie Lacobelle, Catherine Aldi, and Christa Robinson for their excellent technical assistance, to the SSADDA interviewers, led by Yari Nuñez and Michelle Slivinsky, who devoted substantial time and effort to phenotype the study sample, and to John Farrell and Alexan Mardigan for database management assistance.

We are grateful to the Genotype-Tissue Expression (GTEx) Consortium for early-stage sharing of RNA-seq and genotypic data. The GTEx project was supported by the Common Fund of the Office of the NIH Director. Additional funds were provided by the NCI, NHGRI, NHLBI, NIDA, NIMH, and NINDS. Donors were enrolled at Biospecimen Source Sites funded by NCI\SAIC-Frederick, Inc. (SAIC-F) subcontracts to the National Disease Research Interchange (10XS170), Roswell Park Cancer Institute (10XS171), and Science Care, Inc. (X10S172). The Laboratory, Data Analysis, and Coordinating Center (LDACC) was funded through a contract (HHSN268201000029C) to The Broad Institute, Inc. Biorepository operations were funded through an SAIC-F subcontract to Van Andel Institute (10ST1035). Additional data repository and project management were provided by SAIC-F (HHSN261200800001E). The Brain Bank was supported by a supplements to University of Miami grants DA006227 and DA033684 and to contract N01 MH000028. Statistical Methods development grants were made to the University of Geneva (MH090941 and MH101814), the University of Chicago (MH090951, MH090937, MH101820, and MH101825), the University of North Carolina - Chapel Hill (MH090936 and MH101819), Harvard University (MH090948), Stanford University (MH101782), Washington University in St. Louis (MH101810), and the University of Pennsylvania (MH101822).

Funding for lung cancer studies was provided by NCI grant number U19 CA148127.

**References**

1. Thorgeirsson TE, Gudbjartsson DF, Surakka I, Vink JM, Amin N, Geller F *et al.* Sequence variants at CHRNB3-CHRNA6 and CYP2A6 affect smoking behavior. *Nature genetics* 2010; **42**(5)**:** 448-453.

2. Liu JZ, Tozzi F, Waterworth DM, Pillai SG, Muglia P, Middleton L *et al.* Meta-analysis and imputation refines the association of 15q25 with smoking quantity. *Nature genetics* 2010; **42**(5)**:** 436-440.

3. Genome-wide meta-analyses identify multiple loci associated with smoking behavior. *Nature genetics* 2010; **42**(5)**:** 441-447.

4. Saccone NL, Culverhouse RC, Schwantes-An TH, Cannon DS, Chen X, Cichon S *et al.* Multiple independent loci at chromosome 15q25.1 affect smoking quantity: a meta-analysis and comparison with lung cancer and COPD. *PLoS Genet* 2010; **6**(8).

5. Saccone SF, Hinrichs AL, Saccone NL, Chase GA, Konvicka K, Madden PA *et al.* Cholinergic nicotinic receptor genes implicated in a nicotine dependence association study targeting 348 candidate genes with 3713 SNPs. *Humn molecular genetics* 2007; **16**(1)**:** 36-49.

6. Rice JP, Hartz SM, Agrawal A, Almasy L, Bennett S, Breslau N *et al.* CHRNB3 is more strongly associated with Fagerstrom Test for Cigarette Dependence-based nicotine dependence than cigarettes per day: phenotype definition changes genome-wide association studies results. *Addiction* 2012.
